# Colorimetric Metal‐Free Detection of Carbon Monoxide: Reversible CO Uptake by a BNB Frustrated Lewis Pair

**DOI:** 10.1002/anie.202106413

**Published:** 2021-06-22

**Authors:** Xiongfei Zheng, Ili Zulkifly, Andreas Heilmann, Caitilín McManus, Simon Aldridge

**Affiliations:** ^1^ Inorganic Chemistry Laboratory Department of Chemistry University of Oxford South Parks Road Oxford OX1 3QR UK

**Keywords:** borane, carbon monoxide, frustrated Lewis pair, receptor, reversible binding

## Abstract

We report two BNB‐type frustrated Lewis pairs which feature an acceptor‐donor‐acceptor functionalized cavity, and which differ in the nature of the B‐bound fluoroaryl group (C_6_F_5_ vs. C_6_H_3_(CF_3_)_2_‐3,5, Ar^f^). These receptor systems are capable of capturing gaseous CO, and in the case of the ‐BAr^f^_2_ system this can be shown to occur in reversible fashion at/above room temperature. For both systems, the binding event is accompanied by migration of one of the aryl substituents to the electrophilic carbon of the CO guest. Experiments utilizing an additional equivalent of P^t^Bu_3_ allow the initially formed (non‐migrated) CO adduct to be identified and trapped (via demethylation), while also establishing the reversibility of the B‐to‐C migration process. When partnered with the slightly less Lewis acidic ‐BAr^f^_2_ substituent, this reversibility allows for release of the captured carbon monoxide in the temperature range 40–70 °C, and the possibility for CO sensing, making use of the associated colourless to orange/red colour change.

## Graphical abstract

Acridan‐derived receptors featuring an acceptor‐donor‐acceptor functionalized cavity offer a metal‐free approach for binding carbon monoxide. Reversibility can be tuned through borane Lewis acidity and the binding event signalled colorimetrically.

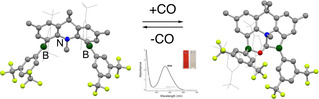

Unlike transition metals which have accessible σ‐ and π‐symmetry frontier orbitals, most p‐block element compounds do not form tractable complexes with CO, primarily due to the lack of suitable orbitals for π‐backbonding. Recently, a number of low‐valent main group compounds have been developed with a suitable orbital manifold to allow for “transition‐metal like” capabilities in the coordination of CO, including isolable carbenes and phosphinidenes, and transient species such as borylenes and silylenes.^[1]^ A number of strongly Lewis acidic boranes can also react with CO to form the corresponding borane–carbonyl complex. OC⋅BH_3_, for example, can be formed by combining B_2_H_6_ with a high pressure of CO, although this adduct dissociates when the CO pressure is reduced.^[2]^ Perfluoroalkylboranes,^[3]^ Piers’ borane,^[4]^ and electron‐deficient boroles^[5]^ have been shown more recently to form the analogous carbonyl complexes, OC⋅BR_3_, which have been structurally characterized by X‐ray crystallography (e.g. Figure 1 A). However, the relatively weak OC−B σ‐bonding and lack of π‐backbonding in these systems typically leads to low thermodynamic stability: OC⋅B(C_6_F_5_)_3_, OC⋅B(CF_3_)_3_, and OC⋅B(C_6_F_5_)_2_H all decompose at room temperature or below, with only the perfluoropentaphenylborole adduct retaining CO at elevated temperatures (up to 60 °C).^[6]^

**Figure 1:**
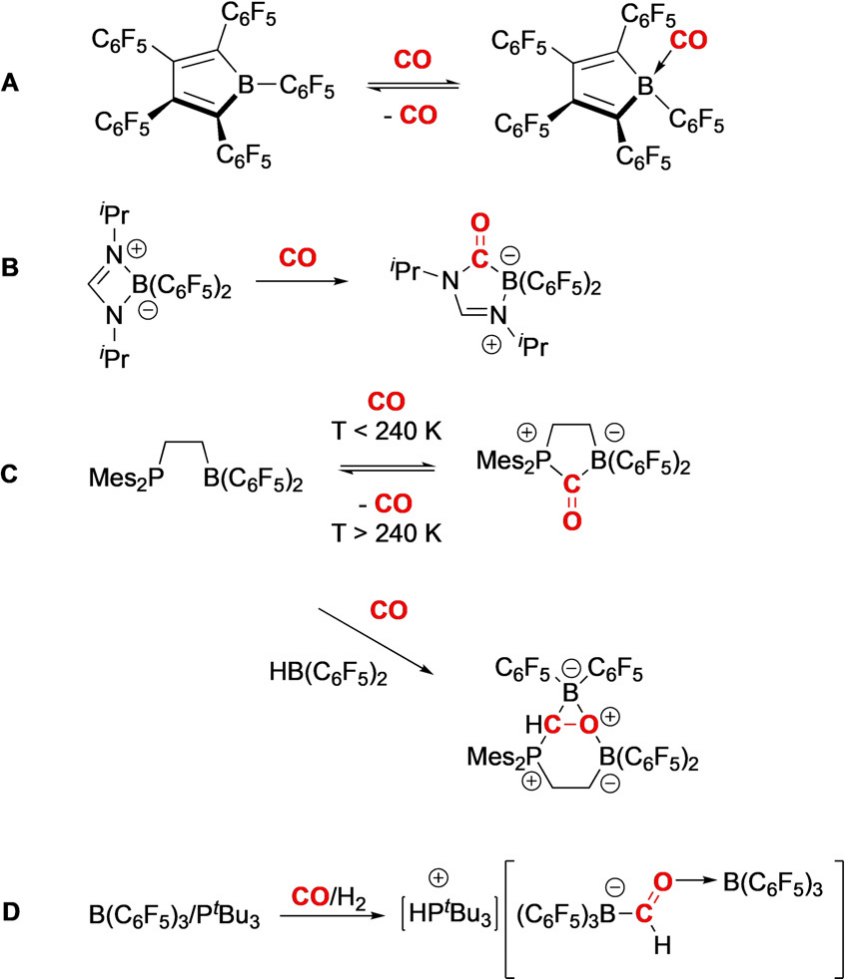
Examples of CO capture by boranes and frustrated Lewis pairs.

In an alternative to a single‐site approach, σ‐donation from CO to a Lewis acid and backbonding from a (separate) Lewis base into the CO π*‐orbital offers the potential for carbon monoxide capture by bifunctional systems.^[7]^ As such, CO uptake has been demonstrated by Stephan via ring‐opening of a boron amidinate complex which acts in cooperative donor–acceptor fashion through its transient “open chain” form (Figure 1 B).^[8]^ Erker et al. have reported single‐component borane/ phosphine frustrated Lewis pairs (FLPs) which react in solution to give CO adducts which are stable at low temperatures (e.g. Figure 1 C);^[9]^ CO capture by analogous borane/amine FLPs has not been reported. In the case of B/P systems, capture can also be combined with chemical modification of the C_1_ unit.[^4^, ^8^, ^9^, ^10^, ^11^, ^12^, ^13^, ^14^] For example, both inter‐ and intramolecular P/B FLPs have been shown to bind CO and transform it via hydrogenation/hydroelementation to yield formyl‐containing products (e.g. Figure 1 C,D).[^10^, ^11a^]

While these examples illustrate potential strategies for capturing carbon monoxide based on its ambiphilic nature, reversibility in CO uptake by FLPs is hard to achieve at room temperature due to the intrinsically weak nature of the interactions involved. We hypothesized that a structural modification to “simple” bifunctional FLPs involving the incorporation of an additional Lewis acid site to bind the O‐terminus of the CO molecule might allow for enhanced binding affinities (Figure 2).^[14]^ Accordingly, we report herein on the synthesis and mode of action of BNB bis‐borane FLPs, one of which can reversibly take up CO at room temperature. Interestingly, this process is assisted by reversible B‐to‐C aryl group migration—a process which has been fully established by spectroscopic, crystallographic, and DFT studies.

**Figure 2:**
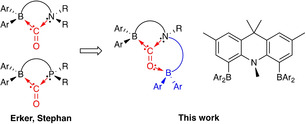
Molecular design strategy: enhancing CO binding by an FLP through the inclusion of a second borane function.

Frustrated Lewis pairs featuring a BNB arrangement of the acid/base functions can readily be synthesized from acridan dibromide **1** via lithiation followed by quenching with the respective chloroborane, ClBAr^*f*^_2_ or ClB(C_6_F_5_)_2_, yielding strongly coloured **2** (orange) or **3** (red) in 86–90 % yield (Scheme 1). The symmetrical substitution pattern at the 4‐ and 5‐positions in each compound is reflected in the observation of three singlets (6:6:3) for the backbone and N‐bound methyl groups, together with a single signal in the ^11^B NMR spectrum (at *δ*_B_ 60 and 61 ppm, respectively) and a ^19^F NMR spectrum consistent with only one type of CF_3_ or C_6_F_5_ group. In the case of **2**, the spectroscopic data could be corroborated by X‐ray crystallography (Scheme 1). By contrast, **3** is very soluble in apolar organic media, making crystallization difficult; proof of connectivity could be obtained, however, through the isolation of the 1:1 (B‐bound) adduct of **3** with ^*t*^Bu_3_PNNO (see Supporting Information).

**Scheme 1:**
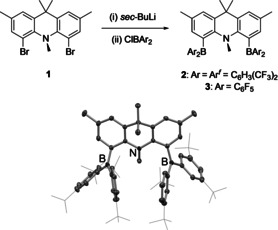
Top: Synthesis of BNB‐type frustrated Lewis pairs **2** and **3**; bottom: molecular structure of **2** in the solid state as determined by X‐ray crystallography. Hydrogen atoms and solvate molecules omitted, and CF_3_ groups shown in wireframe format for clarity.

When treated with CO (at room temperature and ca. 1 atm pressure), both BNB systems give similar products (**4** and **5**; Scheme 2). In the case of **2**, the orange colour of the toluene solution disappears on shaking under a CO atmosphere, and the colourless crystalline product **4** precipitates from the reaction mixture after a few minutes in 70–80 % isolated yield. **5** can be synthesized in a similar way (albeit in lower isolated yield due to its very high solubility), in this case using Me_4_Si to recrystallize the product. The ^1^H NMR spectra of the adducts (**4** and **5**) are very similar, both showing two singlets for the C(CH_3_)_2_ unit and two different aromatic CH_3_ resonances, together with a (shifted) lower‐field signal associated with the NMe function. The ^19^F NMR spectra are consistent with the presence of four distinct CF_3_ (for **4**) or C_6_F_5_ environments (for **5**), implying a significant reduction in symmetry accompanying the binding of CO (see Supporting Information). In the case of **4**, the nature of the product could be confirmed unambiguously by X‐ray crystallography (Figure 3), showing that it results not only from assimilation of carbon monoxide in the BNB FLP pocket, but also from 1,2 aryl group migration from one of the boron centres to the electrophilic carbon derived from the CO molecule.^[15]^ As such, the structure of **4** is based on an approximately tetrahedral carbon centre, featuring a significantly lengthened C−O distance (1.365(3) Å, cf. 1.128 Å for CO and ca. 1.23 Å for the C=O bonds in organic carbonyl compounds).^[16]^

**Figure 3:**
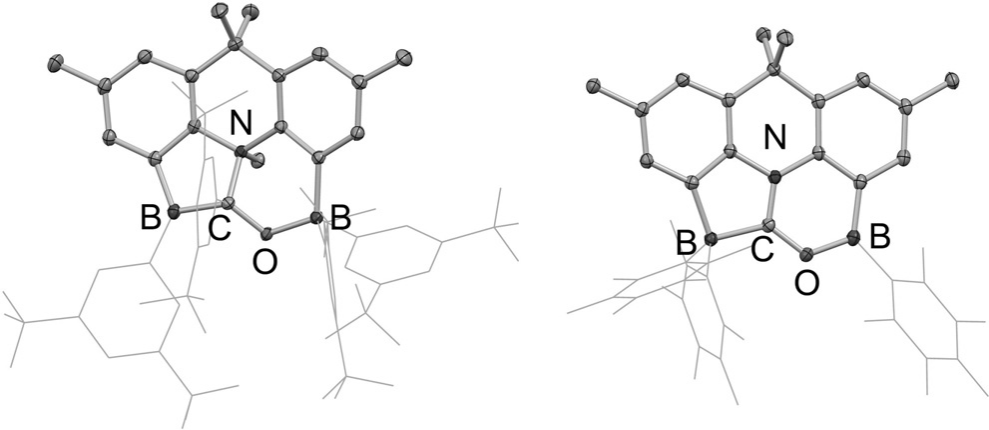
Molecular structures of **4** (left) and **6** (right) in the solid state as determined by X‐ray crystallography. Hydrogen atoms and solvate molecules omitted, and aryl groups shown in wireframe format for clarity. Thermal ellipsoids plotted at the 30 % probability level. Key bond lengths [Å]: (for **4**) B–C 1.614(3), C–O 1.365(2), C–N 1.569(2), B–O 1.544(2); (for **6**): B–C 1.634(2), C–O 1.326(2), C–N 1.318(2), B–O 1.397(2).

**Scheme 2:**
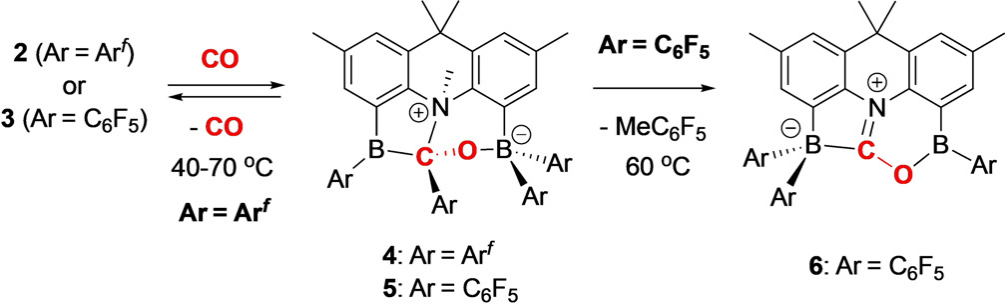
Reactions of BNB systems **2** and **3** with carbon monoxide: CO capture and aryl group migration.

Interestingly, **4** and **5** undergo different reactions when heated. In the case of the Ar^*f*^ derivative **4**, CO is re‐released at slightly elevated temperatures to regenerate **2**. The release of CO can be followed spectroscopically: in situ monitoring by ^1^H NMR in CDCl_3_ in the temperature range 40 to 70 °C allows for the thermodynamic parameters associated with the regeneration of **2** to be determined (Figure 4 and Supporting Information). A van't Hoff plot yields Δ*H* ^o^=87.1 kJ mol^−1^ and Δ*S* ^o^=257.5 J mol^−1^ K^−1^ for CO loss. The evolution of CO is also accompanied by regeneration of the orange colour characteristic of **2** (Figure 4), suggesting potential applications of such systems in the sensing of carbon monoxide.^[17]^

**Figure 4:**
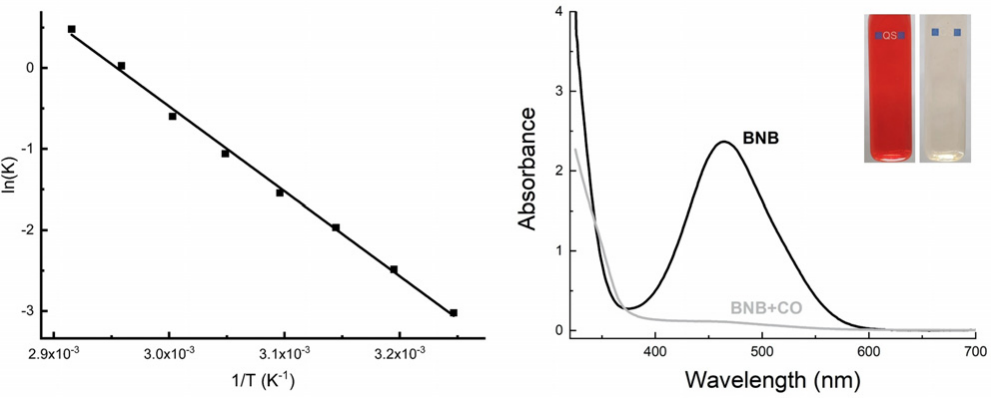
Left: van't Hoff plot relating to the release of CO from **4** in the temperature range 40–70 °C; right: UV/Vis spectra of **2** (black trace) and **4** (grey trace) in chloroform (ca. 0.8 mm concentration); inset: photographs of solutions of **2** and **4** in chloroform.

By contrast, heating **5** to 80 °C leads to the elimination of MeC_6_F_5_ (as shown by ^1^H and ^19^F NMR measurements) to generate zwitterion **6** (Scheme 2). The ^1^H NMR spectrum of **6** is consistent with the loss of the N‐bound Me group, and the ^19^F NMR spectrum shows two sets of signals integrating in the ratio 1:2; the associated signals for the *para*‐CF units (−146.6 and −158.6 ppm, respectively) are consistent with C_6_F_5_ units bound to three‐ and four‐coordinate boron centres, respectively. The structure of **6** was ultimately determined by X‐ray crystallography (Figure 3), and confirms that it is formed via formal loss of MeC_6_F_5_ across the C−N bond of **5**. The short C−N distance (1.318(2) Å) and planar arrangement of atoms around carbon (sum of angles=360.0°) are consistent with the formation of a C=N double bond, and consequent formulation of **6** as an iminium borate species.

We postulate that the differing behaviour of **4** and **5** at elevated temperatures reflects differences in the Lewis acidities of boranes bearing C_6_F_5_ and Ar^*f*^ functions. Aryl migration is shown to be reversible in both cases (see below), and the more modest Lewis acidity associated with the Ar^*f*^ substituent ultimately translates into weaker binding of the CO molecule, and the ability of **2** to reversibly capture carbon monoxide. **5**, by contrast, is prone to rearrangement before a comparable CO release can occur.

Although not directly observable in the reaction of either **2** or **3** with carbon monoxide, we hypothesize that the first‐formed species in the uptake of CO is a simple BNB⋅CO adduct of the form postulated in Figure 2 (see also Scheme 3). DFT calculations on the Ar^*f*^ systems indicate that the free energy of **4** is only marginally lower than that of this intermediate, **Int** (ca. 2 kJ mol^−1^ at the PBE1PBE/TZVP level). With this in mind, we attempted to trap **Int** via reactions with Lewis bases. While the addition of CO to a solution containing **2** and DMAP (*N*,*N*‐dimethylaminopyridine) simply leads to the isolation of the corresponding B‐bound adduct of **4** (i.e. **7**, Scheme 3; see Supporting Information for the X‐ray crystal structure), reactions with more sterically encumbered (and softer) phosphine bases such as P^*t*^Bu_3_ proceed via nucleophilic attack at the N−Me group of **Int**. This demethylation process generates the [MeP^*t*^Bu_3_]^+^ cation, together with the CO adduct of the corresponding [BNB]^−^ amido diboryl receptor. Thus **8**, and its C_6_F_5_ analogue **9**, can be accessed in good yield by adding CO to an equimolar mixture of the respective FLP (**2** or **3**) and P^*t*^Bu_3_.

**Scheme 3:**
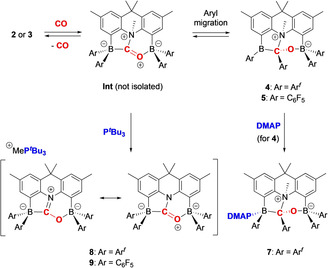
Trapping of the BNB⋅CO adduct intermediate for both **2** and **3** via reaction with P^*t*^Bu_3_.

Spectroscopically, both **8** and **9** are characterized by resonances corresponding to the [MeP^*t*^Bu_3_]^+^ cation (e.g. *δ*_P_ ca. 49 ppm), and each features two signals in the ^11^B NMR spectrum at *δ*_B_<0, indicative of O‐ and C‐ligated four‐coordinate boron centres (e.g. *δ*_B_ −1.1 and −14.9 ppm for **9**). The structures of both salts could also be confirmed crystallographically (see Figure 5 and Supporting Information).

**Figure 5:**
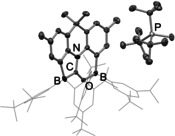
Molecular structure of **8** in the solid state as determined by X‐ray crystallography. Hydrogen atoms and solvate molecules omitted, and aryl groups shown in wireframe format for clarity. Thermal ellipsoids plotted at the 30 % probability level. Key bond lengths: B–C 1.619(8), C–O 1.268(8), C–N 1.346(7), B–O 1.592(8). (The X‐ray crystal structure of **9** is shown in the Supporting Information.)

From a mechanistic perspective it is important to note that phosphonium salts **8** and **9** can both also be accessed from the corresponding aryl migrated species (i.e. **4** or **5**) by the addition of P^*t*^Bu_3_. This observation is consistent with the idea that the B‐to‐C aryl migration process is chemically reversible in each case—a factor that, in the case of the Ar^*f*^ system, ultimately underpins the reversibility of CO uptake.

In summary, we report on two BNB‐type frustrated Lewis pairs featuring an acceptor‐donor‐acceptor functionalized cavity. These receptor systems are capable of capturing gaseous carbon monoxide; in the case of the ‐BAr^*f*^_2_ system, CO uptake can be shown by VT‐NMR studies to occur reversibly. For both systems, the binding event is accompanied by migration of one of the aryl substituents to the electrophilic carbon of the CO guest. Further experiments employing a bulky tertiary phosphine allow the initially formed (non‐migrated) CO adducts to be identified and trapped (via demethylation), while also establishing the reversibility of the B‐to‐C migration process. In conjunction with the slightly less Lewis acidic ‐BAr^*f*^_2_ substituent, this reversibility allows for release of the captured carbon monoxide in the temperature range 40–70 °C, with an associated colourless to orange/red colour change.

Deposition Numbers 2082376, 2082377, 2082378, 2082379, 2082380, 2082381, and 2082382 contain the supplementary crystallographic data for this paper. These data are provided free of charge by the joint Cambridge Crystallographic Data Centre and Fachinformationszentrum Karlsruhe Access Structures service www.ccdc.cam.ac.uk/structures.

## Conflict of interest

The authors declare no conflict of interest.

## Supporting information

As a service to our authors and readers, this journal provides supporting information supplied by the authors. Such materials are peer reviewed and may be re‐organized for online delivery, but are not copy‐edited or typeset. Technical support issues arising from supporting information (other than missing files) should be addressed to the authors.

Supplementary

Supplementary
