# Supplementary material for: Colorimetric Metal‐Free Detection of Carbon Monoxide: Reversible CO Uptake by a BNB Frustrated Lewis Pair
Source: Angew Chem Int Ed Engl. 2021 Jun 22;60(30):16416–9. doi: 10.1002/anie.202106413 (PMC8362209; doi:10.1002/anie.202106413)

checkCIF/PLATON report

Structure factors have been supplied for datablock(s) 150xzah21

THIS REPORT IS FOR GUIDANCE ONLY. IF USED AS PART OF A REVIEW PROCEDURE FOR PUBLICATION, IT SHOULD NOT REPLACE THE EXPERTISE OF AN EXPERIENCED CRYSTALLOGRAPHIC REFEREE.

No syntax errors found. CIF dictionary Interpreting this report

Datablock: 150xzah21

```
Bond precision:  C-C = 0.0026 A         Wavelength=1.54184

Cell:           a=16.4401(1)    b=13.1049(1)       c=25.4034(1)
                alpha=90        beta=103.870(1)    gamma=90
Temperature:    150 K

                Calculated                 Reported
Volume          5313.48(6)                 5313.47(6)
Space group     P 21/c                     P 1 21/c 1
Hall group      -P 2ybc                    -P 2ybc
Moiety formula  C50 H31 B2 F24 N, C6 H6    C50 H31 B2 F24 N, C6 H6
Sum formula     C56 H37 B2 F24 N           C56 H37 B2 F24 N
Mr              1201.49                    1201.48
Dx,g cm-3       1.502                      1.502
Z               4                          4
Mu (mm-1)       1.300                      1.300
F000            2424.0                     2424.0
F000'           2434.95
h,k,lmax        20,16,32                   20,16,32
Nref            11127                      11087
Tmin,Tmax       0.711,0.771                0.839,1.000
Tmin'           0.645

Correction method= # Reported T Limits: Tmin=0.839 Tmax=1.000
AbsCorr = MULTI-SCAN

Data completeness= 0.996          Theta(max)= 76.196

R(reflections)= 0.0547( 10268)    wR2(reflections)= 0.1481( 11087)

S = 1.046                  Npar= 893
```

The following ALERTS were generated. Each ALERT has the format
test-name_ALERT_alert-type_alert-level.
Click on the hyperlinks for more details of the test.

Alert level C

```
PLAT213_ALERT_2_C Atom F9              has ADP max/min Ratio .....        3.2 prolat
PLAT213_ALERT_2_C Atom F10             has ADP max/min Ratio .....        3.1 prolat
PLAT213_ALERT_2_C Atom F18             has ADP max/min Ratio .....        3.2 prolat
PLAT213_ALERT_2_C Atom F16A            has ADP max/min Ratio .....        3.1 prolat
PLAT213_ALERT_2_C Atom F19A            has ADP max/min Ratio .....        3.3 prolat
PLAT215_ALERT_3_C Disordered F22A      has ADP max/min Ratio .....        3.8 Note
PLAT331_ALERT_2_C Small Aver Phenyl C-C Dist C51      --C56      .       1.37 Ang.
PLAT906_ALERT_3_C Large K Value in the Analysis of Variance ......      2.081 Check
```

Alert level G

```
PLAT002_ALERT_2_G Number of Distance or Angle Restraints on AtSite         35 Note
PLAT003_ALERT_2_G Number of Uiso or Uij Restrained non-H Atoms ...         31 Report
PLAT142_ALERT_4_G s.u. on b - Axis Small or Missing .............     0.00010 Ang.
PLAT143_ALERT_4_G s.u. on c - Axis Small or Missing .............     0.00010 Ang.
PLAT153_ALERT_1_G The s.u.'s on the Cell Axes   are Equal ..(Note)     0.0001 Ang.
PLAT172_ALERT_4_G The CIF-Embedded .res File Contains DFIX Records          5 Report
PLAT173_ALERT_4_G The CIF-Embedded .res File Contains DANG Records         24 Report
PLAT178_ALERT_4_G The CIF-Embedded .res File Contains SIMU Records          6 Report
PLAT230_ALERT_2_G Hirshfeld Test Diff for    F3       --C25      .        9.0 s.u.
PLAT230_ALERT_2_G Hirshfeld Test Diff for    F18A     --C42      .        5.7 s.u.
PLAT242_ALERT_2_G Low    'MainMol' Ueq as Compared to Neighbors of        C25 Check
PLAT242_ALERT_2_G Low    'MainMol' Ueq as Compared to Neighbors of        C26 Check
PLAT242_ALERT_2_G Low    'MainMol' Ueq as Compared to Neighbors of        C33 Check
PLAT242_ALERT_2_G Low    'MainMol' Ueq as Compared to Neighbors of        C34 Check
PLAT242_ALERT_2_G Low    'MainMol' Ueq as Compared to Neighbors of        C41 Check
PLAT242_ALERT_2_G Low    'MainMol' Ueq as Compared to Neighbors of        C42 Check
PLAT242_ALERT_2_G Low    'MainMol' Ueq as Compared to Neighbors of        C49 Check
PLAT242_ALERT_2_G Low    'MainMol' Ueq as Compared to Neighbors of        C50 Check
PLAT301_ALERT_3_G Main Residue  Disorder ..............(Resd  1  )        19% Note
PLAT434_ALERT_2_G Short Inter HL..HL Contact F7       ..F18              2.83 Ang.
                                          1-x,-1/2+y,3/2-z  =      2_646 Check
PLAT860_ALERT_3_G Number of Least-Squares Restraints .............        522 Note
PLAT910_ALERT_3_G Missing # of FCF Reflection(s) Below Theta(Min).          1 Note
PLAT912_ALERT_4_G Missing # of FCF Reflections Above STh/L=  0.600         39 Note
PLAT913_ALERT_3_G Missing # of Very Strong Reflections in FCF ....          1 Note
PLAT978_ALERT_2_G Number C-C Bonds with Positive Residual Density.          1 Info
```

---

0 **ALERT level A** = Most likely a serious problem - resolve or explain
0 **ALERT level B** = A potentially serious problem, consider carefully
8 **ALERT level C** = Check. Ensure it is not caused by an omission or oversight
25 **ALERT level G** = General information/check it is not something unexpected

```
 1 ALERT type 1 CIF construction/syntax error, inconsistent or missing data
20 ALERT type 2 Indicator that the structure model may be wrong or deficient
 6 ALERT type 3 Indicator that the structure quality may be low
 6 ALERT type 4 Improvement, methodology, query or suggestion
 0 ALERT type 5 Informative message, check
```

It is advisable to attempt to resolve as many as possible of the alerts in all categories. Often the minor alerts point to easily fixed oversights, errors and omissions in your CIF or refinement strategy, so attention to these fine details can be worthwhile. In order to resolve some of the more serious problems it may be necessary to carry out additional measurements or structure refinements. However, the purpose of your study may justify the reported deviations and the more serious of these should normally be commented upon in the discussion or experimental section of a paper or in the "special_details" fields of the CIF. checkCIF was carefully designed to identify outliers and unusual parameters, but every test has its limitations and alerts that are not important in a particular case may appear. Conversely, the absence of alerts does not guarantee there are no aspects of the results needing attention. It is up to the individual to critically assess their own results and, if necessary, seek expert advice.

Publication of your CIF in IUCr journals

A basic structural check has been run on your CIF. These basic checks will be run on all CIFs submitted for publication in IUCr journals (*Acta Crystallographica*, *Journal of Applied Crystallography*, *Journal of Synchrotron Radiation*); however, if you intend to submit to *Acta Crystallographica Section C* or *E* or *IUCrData*, you should make sure that full publication checks are run on the final version of your CIF prior to submission.

Publication of your CIF in other journals

Please refer to the *Notes for Authors* of the relevant journal for any special instructions relating to CIF submission.

PLATON version of 22/03/2021; check.def file version of 19/03/2021

Datablock 150xzah21 - ellipsoid plot

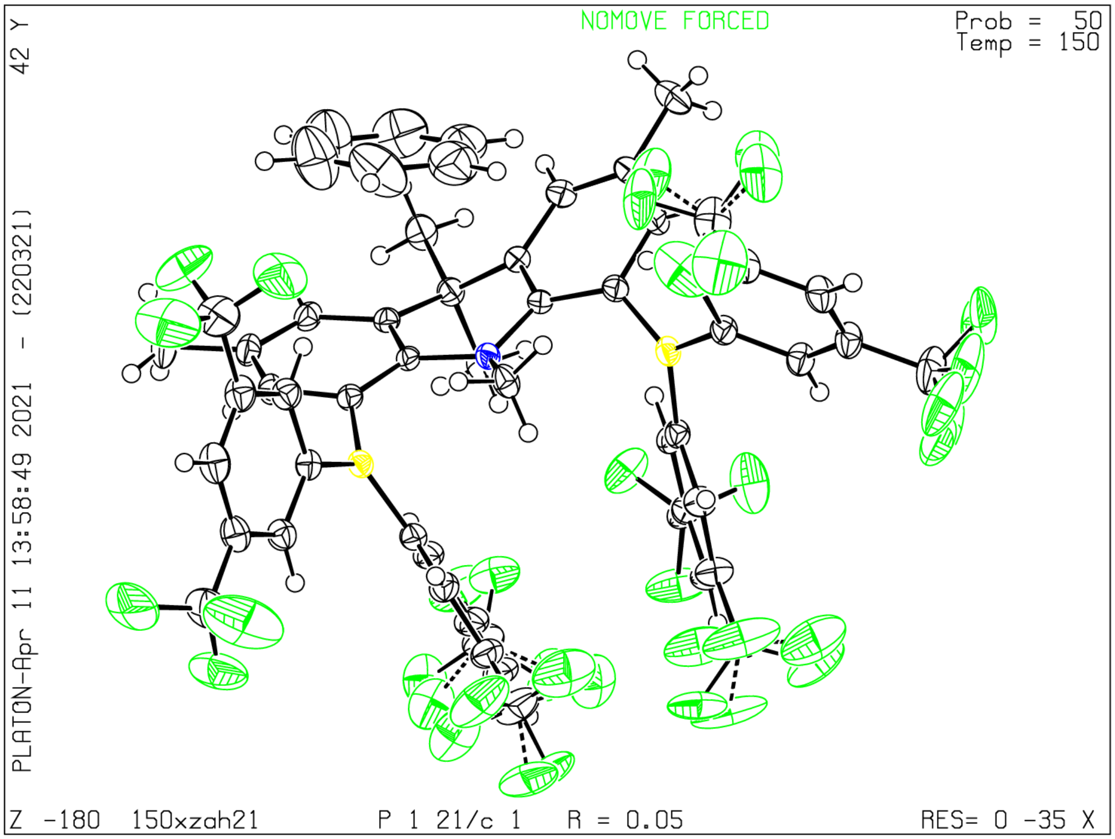

Supplement: Supplementary file 1 — Supplementary [file ANIE-60-16416-s001.zip › 2_checkcif.pdf]
