# Supplementary material for: Colorimetric Metal‐Free Detection of Carbon Monoxide: Reversible CO Uptake by a BNB Frustrated Lewis Pair
Source: Angew Chem Int Ed Engl. 2021 Jun 22;60(30):16416–9. doi: 10.1002/anie.202106413 (PMC8362209; doi:10.1002/anie.202106413)

checkCIF/PLATON report

Structure factors have been supplied for datablock(s) 122xfah20

THIS REPORT IS FOR GUIDANCE ONLY. IF USED AS PART OF A REVIEW PROCEDURE FOR PUBLICATION, IT SHOULD NOT REPLACE THE EXPERTISE OF AN EXPERIENCED CRYSTALLOGRAPHIC REFEREE.

No syntax errors found. CIF dictionary Interpreting this report

Datablock: 122xfah20

Bond precision: C-C = 0.0031 A Wavelength=1.54184

Cell:	a=12.8335(1)	b=23.3049(2)	c=17.5085(1)
	alpha=90	beta=92.485(1)	gamma=90
Temperature:	150 K		

	Calculated	Reported
Volume	5231.58(7)	5231.58(7)
Space group	P 21/n	P 1 21/n 1
Hall group	-P 2yn	-P 2yn
Moiety formula	2(C51 H31 B2 F24 N O), C7 H8	2(C51 H31 B2 F24 N O), C7 H8
Sum formula	C109 H70 B4 F48 N2 O2	C109 H70 B4 F48 N2 O2
Mr	2394.91	2394.91
Dx,g cm-3	1.520	1.520
Z	2	2
Mu (mm-1)	1.333	1.333
F000	2412.0	2412.0
F000’	2423.05	
h,k,lmax	16,29,22	16,29,22
Nref	11020	10981
Tmin,Tmax	0.763,0.936	0.602,1.000
Tmin’	0.638	

Correction method= # Reported T Limits: Tmin=0.602 Tmax=1.000
AbsCorr = MULTI-SCAN

Data completeness= 0.996 Theta(max)= 76.705

R(reflections)= 0.0613(9212) wR2(reflections)= 0.1712(10981)

S = 1.062 Npar= 932

```
The following ALERTS were generated. Each ALERT has the format
       test-name_ALERT_alert-type_alert-level.
Click on the hyperlinks for more details of the test.
```

Alert level C

```
PLAT213_ALERT_2_C Atom F22A           has ADP max/min Ratio .....        3.3 prolat
PLAT250_ALERT_2_C Large U3/U1 Ratio for Average U(i,j) Tensor ....       3.2 Note
PLAT250_ALERT_2_C Large U3/U1 Ratio for Average U(i,j) Tensor ....       3.4 Note
PLAT906_ALERT_3_C Large K Value in the Analysis of Variance ......     2.285 Check
```

Alert level G

```
PLAT002_ALERT_2_G Number of Distance or Angle Restraints on AtSite        37 Note
PLAT003_ALERT_2_G Number of Uiso or Uij Restrained non-H Atoms ...        40 Report
PLAT142_ALERT_4_G s.u. on b - Axis Small or Missing .............    0.00020 Ang.
PLAT143_ALERT_4_G s.u. on c - Axis Small or Missing .............    0.00010 Ang.
PLAT172_ALERT_4_G The CIF-Embedded .res File Contains DFIX Records         7 Report
PLAT173_ALERT_4_G The CIF-Embedded .res File Contains DANG Records         9 Report
PLAT178_ALERT_4_G The CIF-Embedded .res File Contains SIMU Records         5 Report
PLAT230_ALERT_2_G Hirshfeld Test Diff for    F15A     --C42      .       6.3 s.u.
PLAT230_ALERT_2_G Hirshfeld Test Diff for    F14      --C42      .       8.5 s.u.
PLAT230_ALERT_2_G Hirshfeld Test Diff for    F15      --C42      .       7.5 s.u.
PLAT242_ALERT_2_G Low    'MainMol' Ueq as Compared to Neighbors of       C25 Check
PLAT242_ALERT_2_G Low    'MainMol' Ueq as Compared to Neighbors of       C26 Check
PLAT242_ALERT_2_G Low    'MainMol' Ueq as Compared to Neighbors of       C34 Check
PLAT242_ALERT_2_G Low    'MainMol' Ueq as Compared to Neighbors of       C35 Check
PLAT242_ALERT_2_G Low    'MainMol' Ueq as Compared to Neighbors of       C42 Check
PLAT242_ALERT_2_G Low    'MainMol' Ueq as Compared to Neighbors of       C43 Check
PLAT242_ALERT_2_G Low    'MainMol' Ueq as Compared to Neighbors of       C51 Check
PLAT301_ALERT_3_G Main Residue  Disorder .............(Resd  1  )       16% Note
PLAT302_ALERT_4_G Anion/Solvent/Minor-Residue Disorder (Resd  2  )      100% Note
PLAT302_ALERT_4_G Anion/Solvent/Minor-Residue Disorder (Resd  3  )      100% Note
PLAT304_ALERT_4_G Non-Integer Number of Atoms in ..... (Resd  2  )      5.12 Check
PLAT304_ALERT_4_G Non-Integer Number of Atoms in ..... (Resd  3  )      2.38 Check
PLAT789_ALERT_4_G Atoms with Negative _atom_site_disorder_group  #        30 Check
PLAT793_ALERT_4_G Model has Chirality at N1          (Centro SPGR)         S Verify
PLAT793_ALERT_4_G Model has Chirality at C27         (Centro SPGR)         S Verify
PLAT860_ALERT_3_G Number of Least-Squares Restraints ............       723 Note
PLAT912_ALERT_4_G Missing # of FCF Reflections Above STh/L=  0.600        39 Note
PLAT978_ALERT_2_G Number C-C Bonds with Positive Residual Density.         4 Info
PLAT992_ALERT_5_G Repd & Actual _reflns_number_gt Values Differ by         2 Check
```

```
   0 ALERT level A = Most likely a serious problem - resolve or explain
   0 ALERT level B = A potentially serious problem, consider carefully
   4 ALERT level C = Check. Ensure it is not caused by an omission or oversight
  29 ALERT level G = General information/check it is not something unexpected

   0 ALERT type 1 CIF construction/syntax error, inconsistent or missing data
  16 ALERT type 2 Indicator that the structure model may be wrong or deficient
   3 ALERT type 3 Indicator that the structure quality may be low
  13 ALERT type 4 Improvement, methodology, query or suggestion
   1 ALERT type 5 Informative message, check
```

It is advisable to attempt to resolve as many as possible of the alerts in all categories. Often the minor alerts point to easily fixed oversights, errors and omissions in your CIF or refinement strategy, so attention to these fine details can be worthwhile. In order to resolve some of the more serious problems it may be necessary to carry out additional measurements or structure refinements. However, the purpose of your study may justify the reported deviations and the more serious of these should normally be commented upon in the discussion or experimental section of a paper or in the "special_details" fields of the CIF. checkCIF was carefully designed to identify outliers and unusual parameters, but every test has its limitations and alerts that are not important in a particular case may appear. Conversely, the absence of alerts does not guarantee there are no aspects of the results needing attention. It is up to the individual to critically assess their own results and, if necessary, seek expert advice.

Publication of your CIF in IUCr journals

A basic structural check has been run on your CIF. These basic checks will be run on all CIFs submitted for publication in IUCr journals (*Acta Crystallographica*, *Journal of Applied Crystallography*, *Journal of Synchrotron Radiation*); however, if you intend to submit to *Acta Crystallographica Section C* or *E* or *IUCrData*, you should make sure that full publication checks are run on the final version of your CIF prior to submission.

Publication of your CIF in other journals

Please refer to the *Notes for Authors* of the relevant journal for any special instructions relating to CIF submission.

PLATON version of 05/12/2020; check.def file version of 05/12/2020

Datablock 122xfah20 - ellipsoid plot

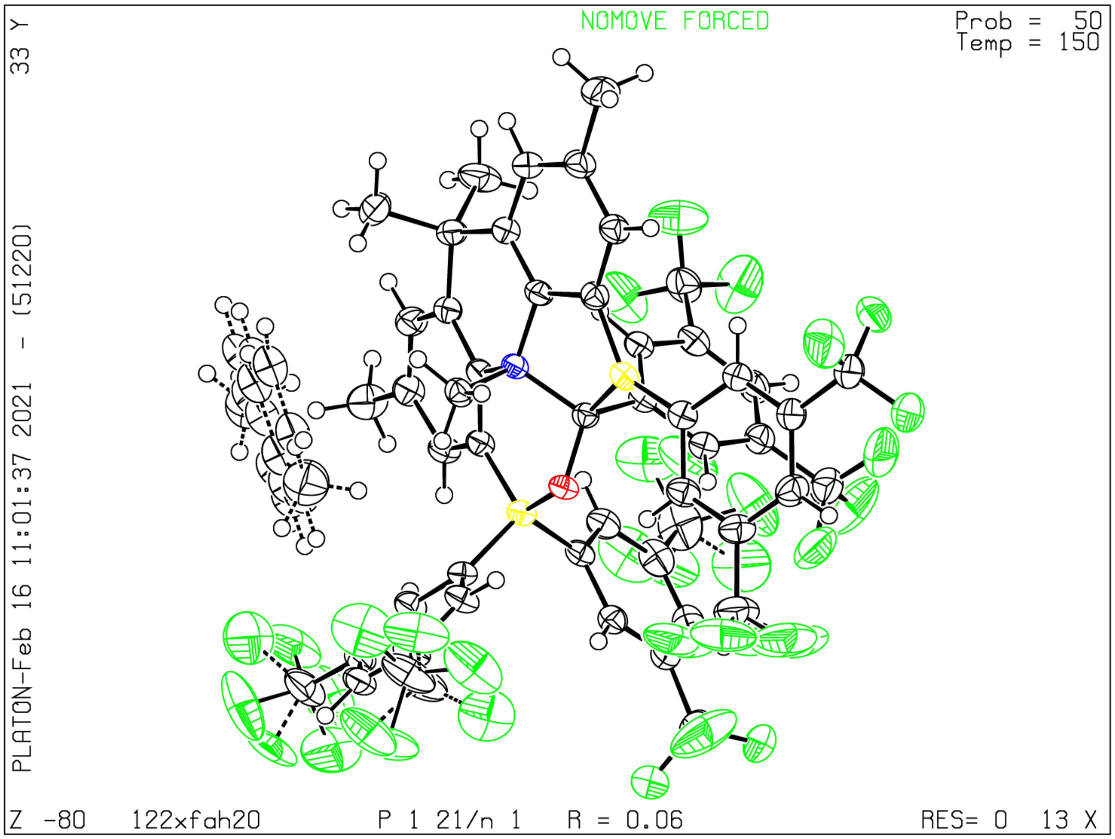

Supplement: Supplementary file 1 — Supplementary [file ANIE-60-16416-s001.zip › 4_checkcif.pdf]
