# Supplementary material for: Colorimetric Metal‐Free Detection of Carbon Monoxide: Reversible CO Uptake by a BNB Frustrated Lewis Pair
Source: Angew Chem Int Ed Engl. 2021 Jun 22;60(30):16416–9. doi: 10.1002/anie.202106413 (PMC8362209; doi:10.1002/anie.202106413)

checkCIF/PLATON report

Structure factors have been supplied for datablock(s) 98zxfcm21

THIS REPORT IS FOR GUIDANCE ONLY. IF USED AS PART OF A REVIEW PROCEDURE FOR PUBLICATION, IT SHOULD NOT REPLACE THE EXPERTISE OF AN EXPERIENCED CRYSTALLOGRAPHIC REFEREE.

No syntax errors found. CIF dictionary Interpreting this report

Datablock: 98zxfcm21

```
Bond precision:  C-C = 0.0030 A          Wavelength=1.54184

Cell:           a=12.2689(5)       b=13.2274(6)      c=13.2354(8)
                alpha=105.177(5)   beta=113.841(5)   gamma=93.386(4)
Temperature:    150 K
```

	Calculated	Reported
Volume	1862.74(19)	1862.75(17)
Space group	P -1	P -1
Hall group	-P 1	-P 1
Moiety formula	C36 H16 B2 F15 N O, C5 H12	C36 H16 B2 F15 N O, C5 H12
Sum formula	C41 H28 B2 F15 N O	C41 H28 B2 F15 N O
Mr	857.26	857.26
Dx,g cm-3	1.528	1.528
Z	2	2
Mu (mm-1)	1.260	1.260
F000	868.0	868.0
F000’	871.76	
h,k,lmax	15,16,16	15,16,16
Nref	7794	7706
Tmin,Tmax	0.900,0.939	0.977,1.000
Tmin’	0.882	

```
Correction method= # Reported T Limits: Tmin=0.977 Tmax=1.000
AbsCorr = MULTI-SCAN

Data completeness= 0.989          Theta(max)= 76.134

R(reflections)= 0.0480( 5945)     wR2(reflections)= 0.1379( 7706)

S = 1.022                  Npar= 595
```

The following ALERTS were generated. Each ALERT has the format
test-name_ALERT_alert-type_alert-level.
Click on the hyperlinks for more details of the test.

Alert level C

```
PLAT094_ALERT_2_C Ratio of Maximum / Minimum Residual Density ....       2.60 Report
PLAT911_ALERT_3_C Missing FCF Refl Between Thmin & STh/L=    0.600          4 Report
```

Alert level G

```
PLAT003_ALERT_2_G Number of Uiso or Uij Restrained non-H Atoms ...         65 Report
PLAT152_ALERT_1_G The Supplied and Calc. Volume s.u. Differ by ...          2 Units
PLAT178_ALERT_4_G The CIF-Embedded .res File Contains SIMU Records          3 Report
PLAT302_ALERT_4_G Anion/Solvent/Minor-Residue Disorder (Resd  2  )       100% Note
PLAT302_ALERT_4_G Anion/Solvent/Minor-Residue Disorder (Resd  3  )       100% Note
PLAT304_ALERT_4_G Non-Integer Number of Atoms in ..... (Resd  2  )      10.54 Check
PLAT304_ALERT_4_G Non-Integer Number of Atoms in ..... (Resd  3  )       6.46 Check
PLAT434_ALERT_2_G Short Inter HL..HL Contact F1       ..F1               2.81 Ang.
                                               1-x,1-y,1-z  =      2_666 Check
PLAT434_ALERT_2_G Short Inter HL..HL Contact F7       ..F15              2.69 Ang.
                                                 -x,1-y,-z  =      2_565 Check
PLAT720_ALERT_4_G Number of Unusual/Non-Standard Labels ..........         31 Note
PLAT860_ALERT_3_G Number of Least-Squares Restraints .............        498 Note
PLAT912_ALERT_4_G Missing # of FCF Reflections Above STh/L=  0.600         84 Note
PLAT941_ALERT_3_G Average HKL Measurement Multiplicity ...........        2.4 Low
PLAT978_ALERT_2_G Number C-C Bonds with Positive Residual Density.         11 Info
PLAT992_ALERT_5_G Repd & Actual _reflns_number_gt Values Differ by          2 Check
```

```
   0 ALERT level A = Most likely a serious problem - resolve or explain
   0 ALERT level B = A potentially serious problem, consider carefully
   2 ALERT level C = Check. Ensure it is not caused by an omission or oversight
  15 ALERT level G = General information/check it is not something unexpected

   1 ALERT type 1 CIF construction/syntax error, inconsistent or missing data
   5 ALERT type 2 Indicator that the structure model may be wrong or deficient
   3 ALERT type 3 Indicator that the structure quality may be low
   7 ALERT type 4 Improvement, methodology, query or suggestion
   1 ALERT type 5 Informative message, check
```

It is advisable to attempt to resolve as many as possible of the alerts in all categories. Often the minor alerts point to easily fixed oversights, errors and omissions in your CIF or refinement strategy, so attention to these fine details can be worthwhile. In order to resolve some of the more serious problems it may be necessary to carry out additional measurements or structure refinements. However, the purpose of your study may justify the reported deviations and the more serious of these should normally be commented upon in the discussion or experimental section of a paper or in the "special_details" fields of the CIF. checkCIF was carefully designed to identify outliers and unusual parameters, but every test has its limitations and alerts that are not important in a particular case may appear. Conversely, the absence of alerts does not guarantee there are no aspects of the results needing attention. It is up to the individual to critically assess their own results and, if necessary, seek expert advice.

Publication of your CIF in IUCr journals

A basic structural check has been run on your CIF. These basic checks will be run on all CIFs submitted for publication in IUCr journals (*Acta Crystallographica*, *Journal of Applied Crystallography*, *Journal of Synchrotron Radiation*); however, if you intend to submit to *Acta Crystallographica Section C* or *E* or *IUCrData*, you should make sure that full publication checks are run on the final version of your CIF prior to submission.

Publication of your CIF in other journals

Please refer to the *Notes for Authors* of the relevant journal for any special instructions relating to CIF submission.

PLATON version of 05/12/2020; check.def file version of 05/12/2020

Datablock 98zxfcm21 - ellipsoid plot

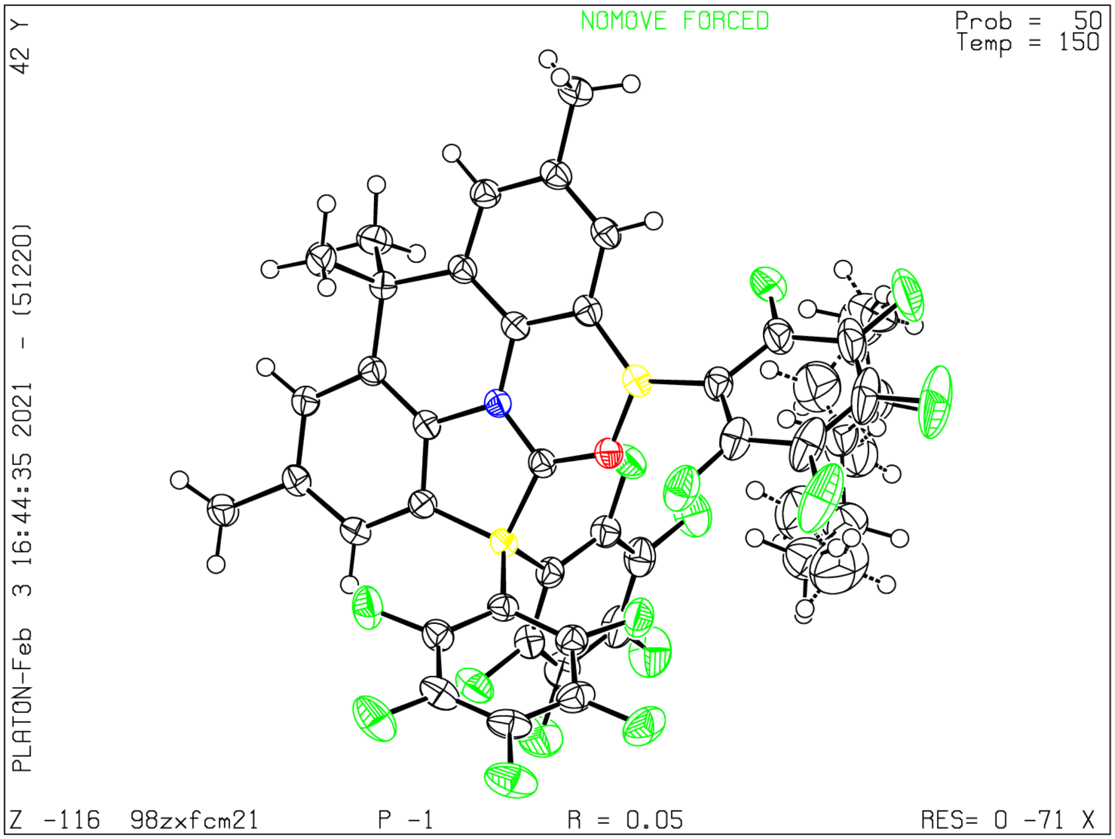

Supplement: Supplementary file 1 — Supplementary [file ANIE-60-16416-s001.zip › 6_checkcif.pdf]
