# Supplementary material for: Colorimetric Metal‐Free Detection of Carbon Monoxide: Reversible CO Uptake by a BNB Frustrated Lewis Pair
Source: Angew Chem Int Ed Engl. 2021 Jun 22;60(30):16416–9. doi: 10.1002/anie.202106413 (PMC8362209; doi:10.1002/anie.202106413)

checkCIF/PLATON report

Structure factors have been supplied for datablock(s) 134xfah20

THIS REPORT IS FOR GUIDANCE ONLY. IF USED AS PART OF A REVIEW PROCEDURE FOR PUBLICATION, IT SHOULD NOT REPLACE THE EXPERTISE OF AN EXPERIENCED CRYSTALLOGRAPHIC REFEREE.

No syntax errors found. CIF dictionary Interpreting this report

Datablock: 134xfah20

Bond precision: C-C = 0.0047 A Wavelength=1.54184

Cell:	a=12.5944(5)	b=15.0257(7)	c=19.6564(6)
	alpha=106.987(3)	beta=92.159(3)	gamma=112.386(4)
Temperature:	150 K		

	Calculated	Reported
Volume	3242.4(3)	3242.4(2)
Space group	P -1	P -1
Hall group	-P 1	-P 1
Moiety formula	C58 H41 B2 F24 N3 O, 2(C H Cl3)	C58 H41 B2 F24 N3 O, 2(C H Cl3)
Sum formula	C60 H43 B2 Cl6 F24 N3 O	C60 H43 B2 Cl6 F24 N3 O
Mr	1512.30	1512.29
Dx,g cm-3	1.549	1.549
Z	2	2
Mu (mm-1)	3.437	3.437
F000	1520.0	1520.0
F000’	1530.23	
h,k,lmax	15,18,24	15,18,24
Nref	13675	13444
Tmin,Tmax	0.745,0.934	0.895,1.000
Tmin’	0.675	

Correction method= # Reported T Limits: Tmin=0.895 Tmax=1.000
AbsCorr = MULTI-SCAN

Data completeness= 0.983 Theta(max)= 76.871

R(reflections)= 0.0738(9798) wR2(reflections)= 0.2291(13444)

S = 1.058 Npar= 1058

The following ALERTS were generated. Each ALERT has the format
test-name_ALERT_alert-type_alert-level.
Click on the hyperlinks for more details of the test.

Alert level C

```
PLAT213_ALERT_2_C Atom F6              has ADP max/min Ratio .....         3.7 prolat
PLAT230_ALERT_2_C Hirshfeld Test Diff for    F15      --C40      .         5.3 s.u.
PLAT260_ALERT_2_C Large Average Ueq of Residue Including       Cl4       0.158 Check
PLAT260_ALERT_2_C Large Average Ueq of Residue Including      Cl1A       0.103 Check
PLAT260_ALERT_2_C Large Average Ueq of Residue Including      Cl4A       0.140 Check
PLAT340_ALERT_3_C Low Bond Precision on  C-C Bonds ...............     0.00471 Ang.
PLAT906_ALERT_3_C Large K Value in the Analysis of Variance ......       2.441 Check
PLAT911_ALERT_3_C Missing FCF Refl Between Thmin & STh/L=    0.600           6 Report
```

Alert level G

```
PLAT002_ALERT_2_G Number of Distance or Angle Restraints on AtSite          37 Note
PLAT003_ALERT_2_G Number of Uiso or Uij Restrained non-H Atoms ...          34 Report
PLAT072_ALERT_2_G SHELXL First  Parameter in WGHT  Unusually Large        0.12 Report
PLAT172_ALERT_4_G The CIF-Embedded .res File Contains DFIX Records           7 Report
PLAT173_ALERT_4_G The CIF-Embedded .res File Contains DANG Records          10 Report
PLAT178_ALERT_4_G The CIF-Embedded .res File Contains SIMU Records           7 Report
PLAT230_ALERT_2_G Hirshfeld Test Diff for    F12      --C33      .         6.0 s.u.
PLAT230_ALERT_2_G Hirshfeld Test Diff for    F10A     --C33      .         5.7 s.u.
PLAT230_ALERT_2_G Hirshfeld Test Diff for    F11A     --C33      .         5.5 s.u.
PLAT230_ALERT_2_G Hirshfeld Test Diff for    F18      --C41      .         5.5 s.u.
PLAT242_ALERT_2_G Low    'MainMol' Ueq as Compared to Neighbors of         C24 Check
PLAT242_ALERT_2_G Low    'MainMol' Ueq as Compared to Neighbors of         C25 Check
PLAT242_ALERT_2_G Low    'MainMol' Ueq as Compared to Neighbors of         C32 Check
PLAT242_ALERT_2_G Low    'MainMol' Ueq as Compared to Neighbors of         C33 Check
PLAT242_ALERT_2_G Low    'MainMol' Ueq as Compared to Neighbors of         C40 Check
PLAT242_ALERT_2_G Low    'MainMol' Ueq as Compared to Neighbors of         C41 Check
PLAT242_ALERT_2_G Low    'MainMol' Ueq as Compared to Neighbors of         C48 Check
PLAT242_ALERT_2_G Low    'MainMol' Ueq as Compared to Neighbors of         C49 Check
PLAT301_ALERT_3_G Main Residue  Disorder ..............(Resd  1  )         14% Note
PLAT302_ALERT_4_G Anion/Solvent/Minor-Residue Disorder (Resd  2  )        100% Note
PLAT302_ALERT_4_G Anion/Solvent/Minor-Residue Disorder (Resd  3  )        100% Note
PLAT302_ALERT_4_G Anion/Solvent/Minor-Residue Disorder (Resd  4  )        100% Note
PLAT302_ALERT_4_G Anion/Solvent/Minor-Residue Disorder (Resd  5  )        100% Note
PLAT304_ALERT_4_G Non-Integer Number of Atoms in ..... (Resd  2  )        4.16 Check
PLAT304_ALERT_4_G Non-Integer Number of Atoms in ..... (Resd  3  )        2.59 Check
PLAT304_ALERT_4_G Non-Integer Number of Atoms in ..... (Resd  4  )        0.83 Check
PLAT304_ALERT_4_G Non-Integer Number of Atoms in ..... (Resd  5  )        2.41 Check
PLAT793_ALERT_4_G Model has Chirality at N1          (Centro SPGR)           S Verify
PLAT793_ALERT_4_G Model has Chirality at C51         (Centro SPGR)           S Verify
PLAT860_ALERT_3_G Number of Least-Squares Restraints .............         504 Note
PLAT912_ALERT_4_G Missing # of FCF Reflections Above STh/L=  0.600         225 Note
PLAT941_ALERT_3_G Average HKL Measurement Multiplicity ...........         2.1 Low
PLAT978_ALERT_2_G Number C-C Bonds with Positive Residual Density.           1 Info
PLAT992_ALERT_5_G Repd & Actual _reflns_number_gt Values Differ by           4 Check
```

---

0 **ALERT level A** = Most likely a serious problem - resolve or explain
0 **ALERT level B** = A potentially serious problem, consider carefully
8 **ALERT level C** = Check. Ensure it is not caused by an omission or oversight
34 **ALERT level G** = General information/check it is not something unexpected

0 ALERT type 1 CIF construction/syntax error, inconsistent or missing data

```
21 ALERT type 2 Indicator that the structure model may be wrong or deficient
 6 ALERT type 3 Indicator that the structure quality may be low
14 ALERT type 4 Improvement, methodology, query or suggestion
 1 ALERT type 5 Informative message, check
```

It is advisable to attempt to resolve as many as possible of the alerts in all categories. Often the minor alerts point to easily fixed oversights, errors and omissions in your CIF or refinement strategy, so attention to these fine details can be worthwhile. In order to resolve some of the more serious problems it may be necessary to carry out additional measurements or structure refinements. However, the purpose of your study may justify the reported deviations and the more serious of these should normally be commented upon in the discussion or experimental section of a paper or in the "special_details" fields of the CIF. checkCIF was carefully designed to identify outliers and unusual parameters, but every test has its limitations and alerts that are not important in a particular case may appear. Conversely, the absence of alerts does not guarantee there are no aspects of the results needing attention. It is up to the individual to critically assess their own results and, if necessary, seek expert advice.

Publication of your CIF in IUCr journals

A basic structural check has been run on your CIF. These basic checks will be run on all CIFs submitted for publication in IUCr journals (*Acta Crystallographica*, *Journal of Applied Crystallography*, *Journal of Synchrotron Radiation*); however, if you intend to submit to *Acta Crystallographica Section C* or *E* or *IUCrData*, you should make sure that full publication checks are run on the final version of your CIF prior to submission.

Publication of your CIF in other journals

Please refer to the *Notes for Authors* of the relevant journal for any special instructions relating to CIF submission.

PLATON version of 05/12/2020; check.def file version of 05/12/2020

Datablock 134xfah20 - ellipsoid plot

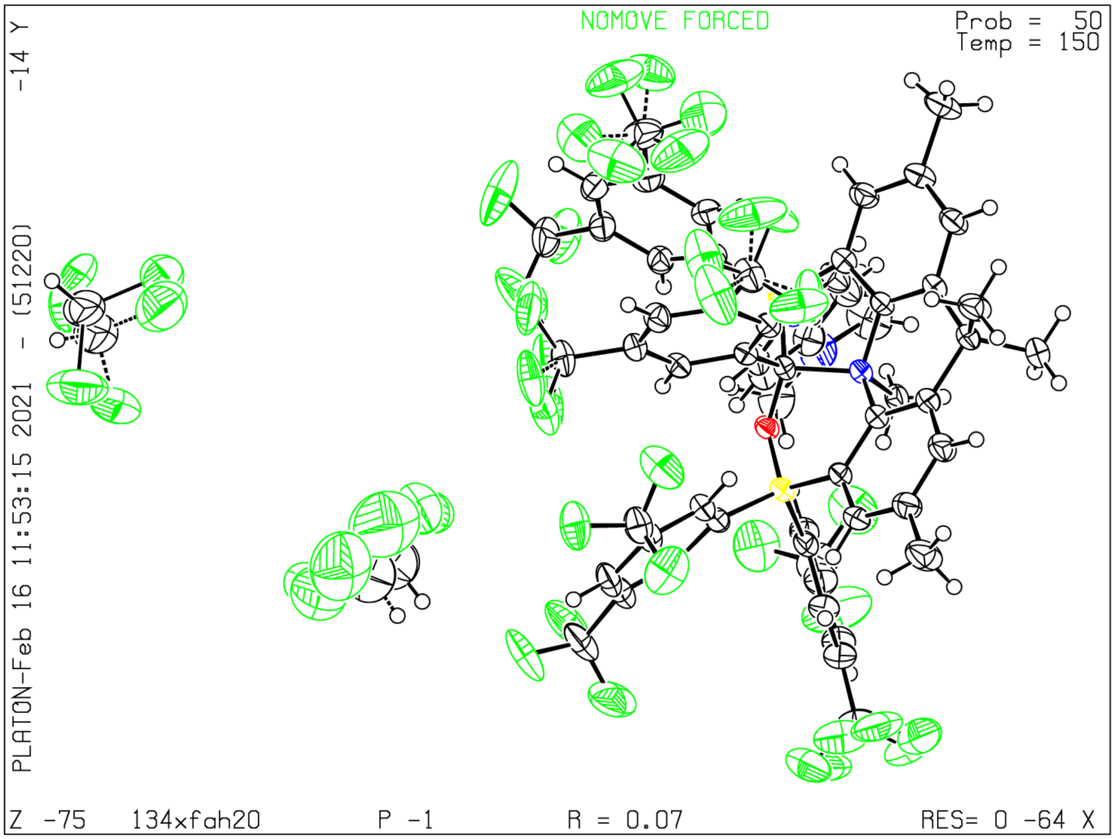

Supplement: Supplementary file 1 — Supplementary [file ANIE-60-16416-s001.zip › 7_checkcif.pdf]
