# Supplementary material for: Colorimetric Metal‐Free Detection of Carbon Monoxide: Reversible CO Uptake by a BNB Frustrated Lewis Pair
Source: Angew Chem Int Ed Engl. 2021 Jun 22;60(30):16416–9. doi: 10.1002/anie.202106413 (PMC8362209; doi:10.1002/anie.202106413)

checkCIF/PLATON report

Structure factors have been supplied for datablock(s) 130xzah20

THIS REPORT IS FOR GUIDANCE ONLY. IF USED AS PART OF A REVIEW PROCEDURE FOR PUBLICATION, IT SHOULD NOT REPLACE THE EXPERTISE OF AN EXPERIENCED CRYSTALLOGRAPHIC REFEREE.

No syntax errors found. CIF dictionary Interpreting this report

Datablock: 130xzah20

Bond precision:	C-C = 0.0067 A		Wavelength=1.54184
Cell:	a=23.8866(3)	b=13.0541(2)	c=21.7644(3)
	alpha=90	beta=90	gamma=90
Temperature:	150 K		

	Calculated	Reported
Volume	6786.53(16)	6786.53(16)
Space group	P n a 21	P n a 21
Hall group	P 2c -2n	P 2c -2n
Moiety formula	C50 H28 B2 F24 N O, C13 H30 P, C6 H6	C13 H30 P, C50 H28 B2 F24 N O, C6 H6
Sum formula	C69 H64 B2 F24 N O P	C69 H64 B2 F24 N O P
Mr	1431.80	1431.80
Dx,g cm-3	1.401	1.401
Z	4	4
Mu (mm-1)	1.334	1.334
F000	2936.0	2936.0
F000’	2949.23	
h,k,lmax	30,16,27	30,16,27
Nref	14315[7353]	11421
Tmin,Tmax	0.880,0.899	0.742,1.000
Tmin’	0.716	

Correction method= # Reported T Limits: Tmin=0.742 Tmax=1.000
AbsCorr = MULTI-SCAN

Data completeness= 1.55/0.80 Theta(max)= 76.830

R(reflections)= 0.0562(10489) wR2(reflections)= 0.1563(11421)

S = 1.045 Npar= 952

The following ALERTS were generated. Each ALERT has the format
      test-name_ALERT_alert-type_alert-level.
Click on the hyperlinks for more details of the test.

Alert level C

```
PLAT202_ALERT_3_C Isotropic non-H Atoms in Anion/Solvent .........          6 Check
              C64A    C65A    C66A    C67A    C68A    C69A
PLAT213_ALERT_2_C Atom F16A            has ADP max/min Ratio .....        3.4 prolat
PLAT213_ALERT_2_C Atom F18A            has ADP max/min Ratio .....        3.1 prolat
PLAT220_ALERT_2_C NonSolvent   Resd 1  C   Ueq(max)/Ueq(min) Range        3.8 Ratio
PLAT340_ALERT_3_C Low Bond Precision on  C-C Bonds ...............    0.00671 Ang.
```

Alert level G

```
PLAT002_ALERT_2_G Number of Distance or Angle Restraints on AtSite         16 Note
PLAT003_ALERT_2_G Number of Uiso or Uij Restrained non-H Atoms ...         31 Report
PLAT042_ALERT_1_G Calc. and Reported MoietyFormula Strings  Differ     Please Check
PLAT172_ALERT_4_G The CIF-Embedded .res File Contains DFIX Records          3 Report
PLAT173_ALERT_4_G The CIF-Embedded .res File Contains DANG Records          2 Report
PLAT178_ALERT_4_G The CIF-Embedded .res File Contains SIMU Records          7 Report
PLAT230_ALERT_2_G Hirshfeld Test Diff for    F5       --C25      .        5.5 s.u.
PLAT230_ALERT_2_G Hirshfeld Test Diff for    F16      --C41      .        5.1 s.u.
PLAT242_ALERT_2_G Low    'MainMol' Ueq as Compared to Neighbors of        C24 Check
PLAT242_ALERT_2_G Low    'MainMol' Ueq as Compared to Neighbors of        C25 Check
PLAT242_ALERT_2_G Low    'MainMol' Ueq as Compared to Neighbors of        C32 Check
PLAT242_ALERT_2_G Low    'MainMol' Ueq as Compared to Neighbors of        C33 Check
PLAT242_ALERT_2_G Low    'MainMol' Ueq as Compared to Neighbors of        C40 Check
PLAT242_ALERT_2_G Low    'MainMol' Ueq as Compared to Neighbors of        C48 Check
PLAT242_ALERT_2_G Low    'MainMol' Ueq as Compared to Neighbors of        C49 Check
PLAT301_ALERT_3_G Main Residue  Disorder .............(Resd  1  )         12% Note
PLAT302_ALERT_4_G Anion/Solvent/Minor-Residue Disorder (Resd  3  )       100% Note
PLAT302_ALERT_4_G Anion/Solvent/Minor-Residue Disorder (Resd  4  )       100% Note
PLAT304_ALERT_4_G Non-Integer Number of Atoms in ..... (Resd  3  )       6.17 Check
PLAT304_ALERT_4_G Non-Integer Number of Atoms in ..... (Resd  4  )       5.83 Check
PLAT434_ALERT_2_G Short Inter HL..HL Contact F21      ..F5A              2.79 Ang.
                                        3/2-x,-1/2+y,-1/2+z  =      4_644 Check
PLAT860_ALERT_3_G Number of Least-Squares Restraints .............        177 Note
PLAT912_ALERT_4_G Missing # of FCF Reflections Above STh/L=  0.600         46 Note
PLAT915_ALERT_3_G No Flack x Check Done: Low Friedel Pair Coverage         59 %
PLAT978_ALERT_2_G Number C-C Bonds with Positive Residual Density.          7 Info
```

---

0 **ALERT level A** = Most likely a serious problem - resolve or explain
0 **ALERT level B** = A potentially serious problem, consider carefully
5 **ALERT level C** = Check. Ensure it is not caused by an omission or oversight
25 **ALERT level G** = General information/check it is not something unexpected

1 ALERT type 1 CIF construction/syntax error, inconsistent or missing data
16 ALERT type 2 Indicator that the structure model may be wrong or deficient
5 ALERT type 3 Indicator that the structure quality may be low
8 ALERT type 4 Improvement, methodology, query or suggestion
0 ALERT type 5 Informative message, check

It is advisable to attempt to resolve as many as possible of the alerts in all categories. Often the minor alerts point to easily fixed oversights, errors and omissions in your CIF or refinement strategy, so attention to these fine details can be worthwhile. In order to resolve some of the more serious problems it may be necessary to carry out additional measurements or structure refinements. However, the purpose of your study may justify the reported deviations and the more serious of these should normally be commented upon in the discussion or experimental section of a paper or in the "special_details" fields of the CIF. checkCIF was carefully designed to identify outliers and unusual parameters, but every test has its limitations and alerts that are not important in a particular case may appear. Conversely, the absence of alerts does not guarantee there are no aspects of the results needing attention. It is up to the individual to critically assess their own results and, if necessary, seek expert advice.

Publication of your CIF in IUCr journals

A basic structural check has been run on your CIF. These basic checks will be run on all CIFs submitted for publication in IUCr journals (*Acta Crystallographica*, *Journal of Applied Crystallography*, *Journal of Synchrotron Radiation*); however, if you intend to submit to *Acta Crystallographica Section C* or *E* or *IUCrData*, you should make sure that full publication checks are run on the final version of your CIF prior to submission.

Publication of your CIF in other journals

Please refer to the *Notes for Authors* of the relevant journal for any special instructions relating to CIF submission.

PLATON version of 05/12/2020; check.def file version of 05/12/2020

Datablock 130xzah20 - ellipsoid plot

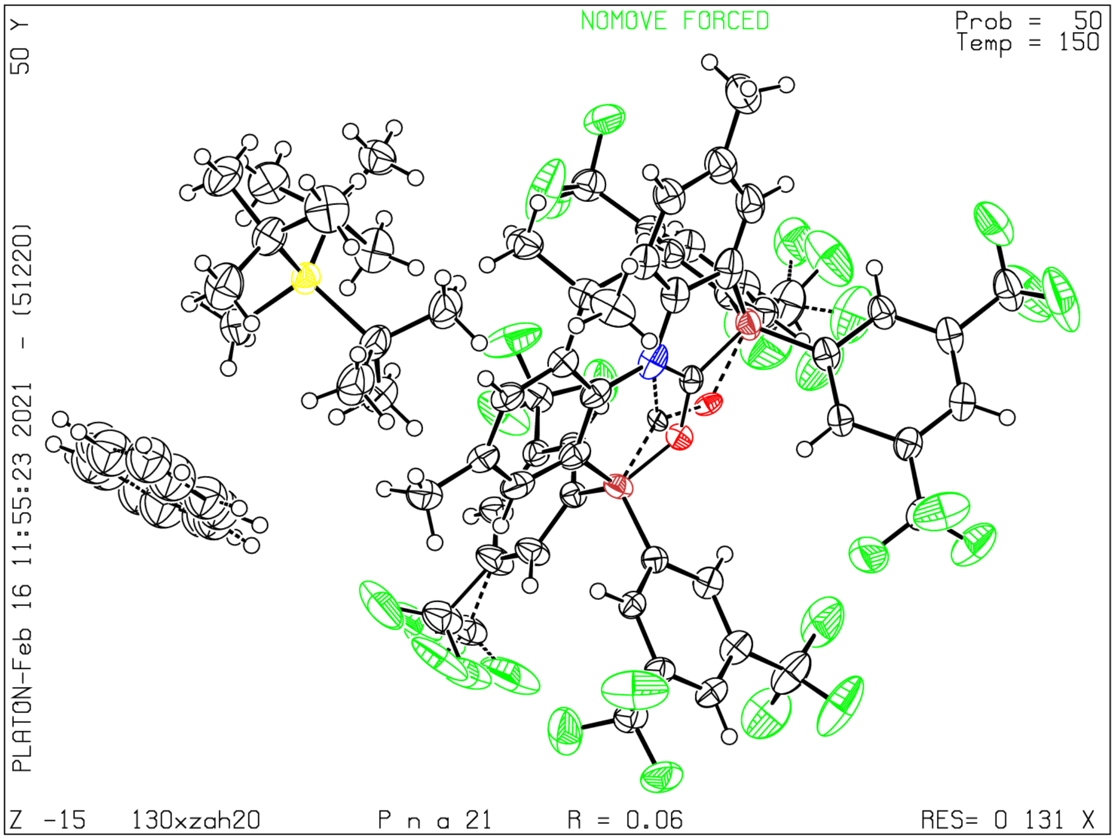

Supplement: Supplementary file 1 — Supplementary [file ANIE-60-16416-s001.zip › 8_checkcif.pdf]
