# Supplementary material for: Colorimetric Metal‐Free Detection of Carbon Monoxide: Reversible CO Uptake by a BNB Frustrated Lewis Pair
Source: Angew Chem Int Ed Engl. 2021 Jun 22;60(30):16416–9. doi: 10.1002/anie.202106413 (PMC8362209; doi:10.1002/anie.202106413)

checkCIF/PLATON report

Structure factors have been supplied for datablock(s) C__ILISTR~1_izcm19_a

THIS REPORT IS FOR GUIDANCE ONLY. IF USED AS PART OF A REVIEW PROCEDURE FOR PUBLICATION, IT SHOULD NOT REPLACE THE EXPERTISE OF AN EXPERIENCED CRYSTALLOGRAPHIC REFEREE.

No syntax errors found. CIF dictionary Interpreting this report

Datablock: C__ILISTR~1_izcm19_a

Bond precision: C-C = 0.0066 A Wavelength=1.54184

Cell:	a=12.8728(2)	b=21.0733(4)	c=59.1848(10)
	alpha=90	beta=94.540(2)	gamma=90
Temperature:	150 K		

	Calculated	Reported
Volume	16004.8(5)	16004.8(5)
Space group	C 2/c	C 2/c
Hall group	-C 2yc	-C 2yc
Moiety formula	3(C42 H16 B2 F20 N O), 3(C13 H30 P), C7 H8	3(C42 H16 B2 F20 N O), 3(C13 H30 P), C7 H8
Sum formula	C172 H146 B6 F60 N3 O3 P3	C172 H146 B6 F60 N3 O3 P3
Mr	3600.69	3600.68
Dx,g cm-3	1.494	1.494
Z	4	4
Mu (mm-1)	1.469	1.469
F000	7352.0	7352.0
F000’	7385.81	
h,k,lmax	16,26,74	16,26,74
Nref	17090	16875
Tmin,Tmax	0.738,0.745	0.811,1.000
Tmin’	0.613	

Correction method= # Reported T Limits: Tmin=0.811 Tmax=1.000
AbsCorr = MULTI-SCAN

Data completeness= 0.987 Theta(max)= 77.769

R(reflections)= 0.0976(14438) wR2(reflections)= 0.2401(16875)

S = 1.099 Npar= 1457

The following ALERTS were generated. Each ALERT has the format
test-name_ALERT_alert-type_alert-level.
Click on the hyperlinks for more details of the test.

Alert level C

```
PLAT230_ALERT_2_C Hirshfeld Test Diff for    C28      --C29      .        6.3 s.u.
PLAT250_ALERT_2_C Large U3/U1 Ratio for Average U(i,j) Tensor ....        2.4 Note
PLAT250_ALERT_2_C Large U3/U1 Ratio for Average U(i,j) Tensor ....        2.2 Note
PLAT250_ALERT_2_C Large U3/U1 Ratio for Average U(i,j) Tensor ....        2.3 Note
PLAT340_ALERT_3_C Low Bond Precision on  C-C Bonds ...............    0.00656 Ang.
PLAT906_ALERT_3_C Large K Value in the Analysis of Variance ......     15.439 Check
PLAT906_ALERT_3_C Large K Value in the Analysis of Variance ......      3.428 Check
PLAT977_ALERT_2_C Check Negative Difference Density on H87F           -0.45 eA-3
```

Alert level G

```
PLAT002_ALERT_2_G Number of Distance or Angle Restraints on AtSite         46 Note
PLAT003_ALERT_2_G Number of Uiso or Uij Restrained non-H Atoms ...         42 Report
PLAT083_ALERT_2_G SHELXL Second Parameter in WGHT  Unusually Large      68.61 Why ?
PLAT172_ALERT_4_G The CIF-Embedded .res File Contains DFIX Records         15 Report
PLAT173_ALERT_4_G The CIF-Embedded .res File Contains DANG Records         19 Report
PLAT174_ALERT_4_G The CIF-Embedded .res File Contains FLAT Records          2 Report
PLAT178_ALERT_4_G The CIF-Embedded .res File Contains SIMU Records          4 Report
PLAT186_ALERT_4_G The CIF-Embedded .res File Contains ISOR Records          1 Report
PLAT300_ALERT_4_G Atom Site Occupancy of O2        Constrained at        0.5 Check
PLAT300_ALERT_4_G Atom Site Occupancy of C56       Constrained at        0.5 Check
PLAT300_ALERT_4_G Atom Site Occupancy of C63       Constrained at        0.5 Check
PLAT300_ALERT_4_G Atom Site Occupancy of C64       Constrained at        0.5 Check
PLAT300_ALERT_4_G Atom Site Occupancy of C64A      Constrained at        0.5 Check
PLAT300_ALERT_4_G Atom Site Occupancy of H64A      Constrained at        0.5 Check
PLAT300_ALERT_4_G Atom Site Occupancy of H64B      Constrained at        0.5 Check
PLAT300_ALERT_4_G Atom Site Occupancy of H64C      Constrained at        0.5 Check
PLAT300_ALERT_4_G Atom Site Occupancy of H64D      Constrained at        0.5 Check
PLAT300_ALERT_4_G Atom Site Occupancy of H64E      Constrained at        0.5 Check
PLAT300_ALERT_4_G Atom Site Occupancy of H64F      Constrained at        0.5 Check
PLAT300_ALERT_4_G Atom Site Occupancy of P2        Constrained at        0.3 Check
PLAT300_ALERT_4_G Atom Site Occupancy of C78       Constrained at        0.3 Check
PLAT300_ALERT_4_G Atom Site Occupancy of C79       Constrained at        0.3 Check
PLAT300_ALERT_4_G Atom Site Occupancy of C80       Constrained at        0.3 Check
PLAT300_ALERT_4_G Atom Site Occupancy of C81       Constrained at        0.3 Check
PLAT300_ALERT_4_G Atom Site Occupancy of C82       Constrained at        0.3 Check
PLAT300_ALERT_4_G Atom Site Occupancy of C83       Constrained at        0.3 Check
PLAT300_ALERT_4_G Atom Site Occupancy of C84       Constrained at        0.3 Check
PLAT300_ALERT_4_G Atom Site Occupancy of C85       Constrained at        0.3 Check
PLAT300_ALERT_4_G Atom Site Occupancy of C86       Constrained at        0.3 Check
PLAT300_ALERT_4_G Atom Site Occupancy of C87       Constrained at        0.3 Check
PLAT300_ALERT_4_G Atom Site Occupancy of C88       Constrained at        0.3 Check
PLAT300_ALERT_4_G Atom Site Occupancy of C89       Constrained at        0.3 Check
PLAT300_ALERT_4_G Atom Site Occupancy of C90       Constrained at        0.3 Check
PLAT300_ALERT_4_G Atom Site Occupancy of H79A      Constrained at        0.3 Check
PLAT300_ALERT_4_G Atom Site Occupancy of H79B      Constrained at        0.3 Check
PLAT300_ALERT_4_G Atom Site Occupancy of H79C      Constrained at        0.3 Check
PLAT300_ALERT_4_G Atom Site Occupancy of H80A      Constrained at        0.3 Check
PLAT300_ALERT_4_G Atom Site Occupancy of H80B      Constrained at        0.3 Check
PLAT300_ALERT_4_G Atom Site Occupancy of H80C      Constrained at        0.3 Check
PLAT300_ALERT_4_G Atom Site Occupancy of H81A      Constrained at        0.3 Check
PLAT300_ALERT_4_G Atom Site Occupancy of H81B      Constrained at        0.3 Check
PLAT300_ALERT_4_G Atom Site Occupancy of H81C      Constrained at        0.3 Check
PLAT300_ALERT_4_G Atom Site Occupancy of H83A      Constrained at        0.3 Check
PLAT300_ALERT_4_G Atom Site Occupancy of H83B      Constrained at        0.3 Check
```

```
PLAT300_ALERT_4_G Atom Site Occupancy of H83C       Constrained at        0.3 Check
PLAT300_ALERT_4_G Atom Site Occupancy of H84A       Constrained at        0.3 Check
PLAT300_ALERT_4_G Atom Site Occupancy of H84B       Constrained at        0.3 Check
PLAT300_ALERT_4_G Atom Site Occupancy of H84C       Constrained at        0.3 Check
PLAT300_ALERT_4_G Atom Site Occupancy of H85A       Constrained at        0.3 Check
PLAT300_ALERT_4_G Atom Site Occupancy of H85B       Constrained at        0.3 Check
PLAT300_ALERT_4_G Atom Site Occupancy of H85C       Constrained at        0.3 Check
PLAT300_ALERT_4_G Atom Site Occupancy of H87A       Constrained at        0.3 Check
PLAT300_ALERT_4_G Atom Site Occupancy of H87B       Constrained at        0.3 Check
PLAT300_ALERT_4_G Atom Site Occupancy of H87C       Constrained at        0.3 Check
PLAT300_ALERT_4_G Atom Site Occupancy of H88A       Constrained at        0.3 Check
PLAT300_ALERT_4_G Atom Site Occupancy of H88B       Constrained at        0.3 Check
PLAT300_ALERT_4_G Atom Site Occupancy of H88C       Constrained at        0.3 Check
PLAT300_ALERT_4_G Atom Site Occupancy of H89A       Constrained at        0.3 Check
PLAT300_ALERT_4_G Atom Site Occupancy of H89B       Constrained at        0.3 Check
PLAT300_ALERT_4_G Atom Site Occupancy of H89C       Constrained at        0.3 Check
PLAT300_ALERT_4_G Atom Site Occupancy of H90A       Constrained at        0.3 Check
PLAT300_ALERT_4_G Atom Site Occupancy of H90B       Constrained at        0.3 Check
PLAT300_ALERT_4_G Atom Site Occupancy of H90C       Constrained at        0.3 Check
PLAT300_ALERT_4_G Atom Site Occupancy of P2A        Constrained at        0.2 Check
PLAT300_ALERT_4_G Atom Site Occupancy of C78A       Constrained at        0.2 Check
PLAT300_ALERT_4_G Atom Site Occupancy of C79A       Constrained at        0.2 Check
PLAT300_ALERT_4_G Atom Site Occupancy of C80A       Constrained at        0.2 Check
PLAT300_ALERT_4_G Atom Site Occupancy of C81A       Constrained at        0.2 Check
PLAT300_ALERT_4_G Atom Site Occupancy of C82A       Constrained at        0.2 Check
PLAT300_ALERT_4_G Atom Site Occupancy of C83A       Constrained at        0.2 Check
PLAT300_ALERT_4_G Atom Site Occupancy of C84A       Constrained at        0.2 Check
PLAT300_ALERT_4_G Atom Site Occupancy of C85A       Constrained at        0.2 Check
PLAT300_ALERT_4_G Atom Site Occupancy of C86A       Constrained at        0.2 Check
PLAT300_ALERT_4_G Atom Site Occupancy of C87A       Constrained at        0.2 Check
PLAT300_ALERT_4_G Atom Site Occupancy of C88A       Constrained at        0.2 Check
PLAT300_ALERT_4_G Atom Site Occupancy of C89A       Constrained at        0.2 Check
PLAT300_ALERT_4_G Atom Site Occupancy of C90A       Constrained at        0.2 Check
PLAT300_ALERT_4_G Atom Site Occupancy of H79D       Constrained at        0.2 Check
PLAT300_ALERT_4_G Atom Site Occupancy of H79E       Constrained at        0.2 Check
PLAT300_ALERT_4_G Atom Site Occupancy of H79F       Constrained at        0.2 Check
PLAT300_ALERT_4_G Atom Site Occupancy of H80D       Constrained at        0.2 Check
PLAT300_ALERT_4_G Atom Site Occupancy of H80E       Constrained at        0.2 Check
PLAT300_ALERT_4_G Atom Site Occupancy of H80F       Constrained at        0.2 Check
PLAT300_ALERT_4_G Atom Site Occupancy of H81D       Constrained at        0.2 Check
PLAT300_ALERT_4_G Atom Site Occupancy of H81E       Constrained at        0.2 Check
PLAT300_ALERT_4_G Atom Site Occupancy of H81F       Constrained at        0.2 Check
PLAT300_ALERT_4_G Atom Site Occupancy of H83D       Constrained at        0.2 Check
PLAT300_ALERT_4_G Atom Site Occupancy of H83E       Constrained at        0.2 Check
PLAT300_ALERT_4_G Atom Site Occupancy of H83F       Constrained at        0.2 Check
PLAT300_ALERT_4_G Atom Site Occupancy of H84D       Constrained at        0.2 Check
PLAT300_ALERT_4_G Atom Site Occupancy of H84E       Constrained at        0.2 Check
PLAT300_ALERT_4_G Atom Site Occupancy of H84F       Constrained at        0.2 Check
PLAT300_ALERT_4_G Atom Site Occupancy of H85D       Constrained at        0.2 Check
PLAT300_ALERT_4_G Atom Site Occupancy of H85E       Constrained at        0.2 Check
PLAT300_ALERT_4_G Atom Site Occupancy of H85F       Constrained at        0.2 Check
PLAT300_ALERT_4_G Atom Site Occupancy of H87D       Constrained at        0.2 Check
PLAT300_ALERT_4_G Atom Site Occupancy of H87E       Constrained at        0.2 Check
PLAT300_ALERT_4_G Atom Site Occupancy of H87F       Constrained at        0.2 Check
PLAT300_ALERT_4_G Atom Site Occupancy of H88D       Constrained at        0.2 Check
PLAT300_ALERT_4_G Atom Site Occupancy of H88E       Constrained at        0.2 Check
PLAT300_ALERT_4_G Atom Site Occupancy of H88F       Constrained at        0.2 Check
PLAT300_ALERT_4_G Atom Site Occupancy of H89D       Constrained at        0.2 Check
PLAT300_ALERT_4_G Atom Site Occupancy of H89E       Constrained at        0.2 Check
PLAT300_ALERT_4_G Atom Site Occupancy of H89F       Constrained at        0.2 Check
PLAT300_ALERT_4_G Atom Site Occupancy of H90D       Constrained at        0.2 Check
PLAT300_ALERT_4_G Atom Site Occupancy of H90E       Constrained at        0.2 Check
```

```
PLAT300_ALERT_4_G Atom Site Occupancy of H90F       Constrained at        0.2 Check
PLAT300_ALERT_4_G Atom Site Occupancy of C91        Constrained at       0.25 Check
PLAT300_ALERT_4_G Atom Site Occupancy of C92        Constrained at       0.25 Check
PLAT300_ALERT_4_G Atom Site Occupancy of C93        Constrained at       0.25 Check
PLAT300_ALERT_4_G Atom Site Occupancy of C94        Constrained at       0.25 Check
PLAT300_ALERT_4_G Atom Site Occupancy of C95        Constrained at       0.25 Check
PLAT300_ALERT_4_G Atom Site Occupancy of C96        Constrained at       0.25 Check
PLAT300_ALERT_4_G Atom Site Occupancy of C97        Constrained at       0.25 Check
PLAT300_ALERT_4_G Atom Site Occupancy of H91        Constrained at       0.25 Check
PLAT300_ALERT_4_G Atom Site Occupancy of H92        Constrained at       0.25 Check
PLAT300_ALERT_4_G Atom Site Occupancy of H93        Constrained at       0.25 Check
PLAT300_ALERT_4_G Atom Site Occupancy of H94        Constrained at       0.25 Check
PLAT300_ALERT_4_G Atom Site Occupancy of H95        Constrained at       0.25 Check
PLAT300_ALERT_4_G Atom Site Occupancy of H97A       Constrained at       0.25 Check
PLAT300_ALERT_4_G Atom Site Occupancy of H97B       Constrained at       0.25 Check
PLAT300_ALERT_4_G Atom Site Occupancy of H97C       Constrained at       0.25 Check
PLAT300_ALERT_4_G Atom Site Occupancy of C91A       Constrained at       0.25 Check
PLAT300_ALERT_4_G Atom Site Occupancy of C92A       Constrained at       0.25 Check
PLAT300_ALERT_4_G Atom Site Occupancy of C93A       Constrained at       0.25 Check
PLAT300_ALERT_4_G Atom Site Occupancy of C94A       Constrained at       0.25 Check
PLAT300_ALERT_4_G Atom Site Occupancy of C95A       Constrained at       0.25 Check
PLAT300_ALERT_4_G Atom Site Occupancy of C96A       Constrained at       0.25 Check
PLAT300_ALERT_4_G Atom Site Occupancy of C97A       Constrained at       0.25 Check
PLAT300_ALERT_4_G Atom Site Occupancy of H91A       Constrained at       0.25 Check
PLAT300_ALERT_4_G Atom Site Occupancy of H92A       Constrained at       0.25 Check
PLAT300_ALERT_4_G Atom Site Occupancy of H93A       Constrained at       0.25 Check
PLAT300_ALERT_4_G Atom Site Occupancy of H94A       Constrained at       0.25 Check
PLAT300_ALERT_4_G Atom Site Occupancy of H95A       Constrained at       0.25 Check
PLAT300_ALERT_4_G Atom Site Occupancy of H97D       Constrained at       0.25 Check
PLAT300_ALERT_4_G Atom Site Occupancy of H97E       Constrained at       0.25 Check
PLAT300_ALERT_4_G Atom Site Occupancy of H97F       Constrained at       0.25 Check
PLAT301_ALERT_3_G Main Residue  Disorder ..............(Resd  1  )         8% Note
PLAT301_ALERT_3_G Main Residue  Disorder ..............(Resd  2  )         3% Note
PLAT302_ALERT_4_G Anion/Solvent/Minor-Residue Disorder (Resd  4  )       100% Note
PLAT302_ALERT_4_G Anion/Solvent/Minor-Residue Disorder (Resd  5  )       100% Note
PLAT302_ALERT_4_G Anion/Solvent/Minor-Residue Disorder (Resd  6  )       100% Note
PLAT302_ALERT_4_G Anion/Solvent/Minor-Residue Disorder (Resd  7  )       100% Note
PLAT304_ALERT_4_G Non-Integer Number of Atoms in ..... (Resd  4  )      13.20 Check
PLAT304_ALERT_4_G Non-Integer Number of Atoms in ..... (Resd  5  )       8.80 Check
PLAT304_ALERT_4_G Non-Integer Number of Atoms in ..... (Resd  6  )       3.75 Check
PLAT304_ALERT_4_G Non-Integer Number of Atoms in ..... (Resd  7  )       3.75 Check
PLAT380_ALERT_4_G Incorrectly? Oriented X(sp2)-Methyl Moiety .....        C15 Check
PLAT412_ALERT_2_G Short Intra XH3 .. XHn     H61      ..H64F     .       2.07 Ang.
                                               -x,y,1/2-z  =      2_555 Check
PLAT432_ALERT_2_G Short Inter X...Y Contact  F11      ..C84A             2.91 Ang.
                                              1-x,y,1/2-z  =      2_655 Check
PLAT432_ALERT_2_G Short Inter X...Y Contact  F11      ..C85              2.96 Ang.
                                              1-x,y,1/2-z  =      2_655 Check
PLAT432_ALERT_2_G Short Inter X...Y Contact  F21      ..C89              2.96 Ang.
                                              1-x,y,1/2-z  =      2_655 Check
PLAT432_ALERT_2_G Short Inter X...Y Contact  F29      ..C81A             2.97 Ang.
                                                 -1+x,y,z  =      1_455 Check
PLAT434_ALERT_2_G Short Inter HL..HL Contact F3       ..F8               2.83 Ang.
                                            1/2+x,1/2+y,z  =      3_555 Check
PLAT434_ALERT_2_G Short Inter HL..HL Contact F4       ..F6               2.84 Ang.
                                          3/2-x,3/2-y,1-z  =      7_666 Check
PLAT434_ALERT_2_G Short Inter HL..HL Contact F9       ..F14              2.74 Ang.
                                          -1/2+x,-1/2+y,z  =      3_445 Check
PLAT434_ALERT_2_G Short Inter HL..HL Contact F18      ..F23              2.80 Ang.
                                                    x,y,z  =      1_555 Check
PLAT789_ALERT_4_G Atoms with Negative _atom_site_disorder_group  #        129 Check
PLAT860_ALERT_3_G Number of Least-Squares Restraints ............         356 Note
```

```
PLAT883_ALERT_1_G No Info/Value for _atom_sites_solution_primary .     Please Do !
PLAT910_ALERT_3_G Missing # of FCF Reflection(s) Below Theta(Min).          1 Note
PLAT912_ALERT_4_G Missing # of FCF Reflections Above STh/L=  0.600        215 Note
PLAT941_ALERT_3_G Average HKL Measurement Multiplicity ...........        4.3 Low
PLAT978_ALERT_2_G Number C-C Bonds with Positive Residual Density.          1 Info
PLAT992_ALERT_5_G Repd & Actual _reflns_number_gt Values Differ by          1 Check
```

```
   0 ALERT level A = Most likely a serious problem - resolve or explain
   0 ALERT level B = A potentially serious problem, consider carefully
   8 ALERT level C = Check. Ensure it is not caused by an omission or oversight
 165 ALERT level G = General information/check it is not something unexpected

   1 ALERT type 1 CIF construction/syntax error, inconsistent or missing data
  18 ALERT type 2 Indicator that the structure model may be wrong or deficient
   8 ALERT type 3 Indicator that the structure quality may be low
 145 ALERT type 4 Improvement, methodology, query or suggestion
   1 ALERT type 5 Informative message, check
```

It is advisable to attempt to resolve as many as possible of the alerts in all categories. Often the minor alerts point to easily fixed oversights, errors and omissions in your CIF or refinement strategy, so attention to these fine details can be worthwhile. In order to resolve some of the more serious problems it may be necessary to carry out additional measurements or structure refinements. However, the purpose of your study may justify the reported deviations and the more serious of these should normally be commented upon in the discussion or experimental section of a paper or in the "special_details" fields of the CIF. checkCIF was carefully designed to identify outliers and unusual parameters, but every test has its limitations and alerts that are not important in a particular case may appear. Conversely, the absence of alerts does not guarantee there are no aspects of the results needing attention. It is up to the individual to critically assess their own results and, if necessary, seek expert advice.

Publication of your CIF in IUCr journals

A basic structural check has been run on your CIF. These basic checks will be run on all CIFs submitted for publication in IUCr journals (*Acta Crystallographica*, *Journal of Applied Crystallography*, *Journal of Synchrotron Radiation*); however, if you intend to submit to *Acta Crystallographica Section C* or *E* or *IUCrData*, you should make sure that full publication checks are run on the final version of your CIF prior to submission.

Publication of your CIF in other journals

Please refer to the *Notes for Authors* of the relevant journal for any special instructions relating to CIF submission.

PLATON version of 16/04/2020; check.def file version of 09/03/2020

Datablock C__ILISTR~1_izcm19_a - ellipsoid plot

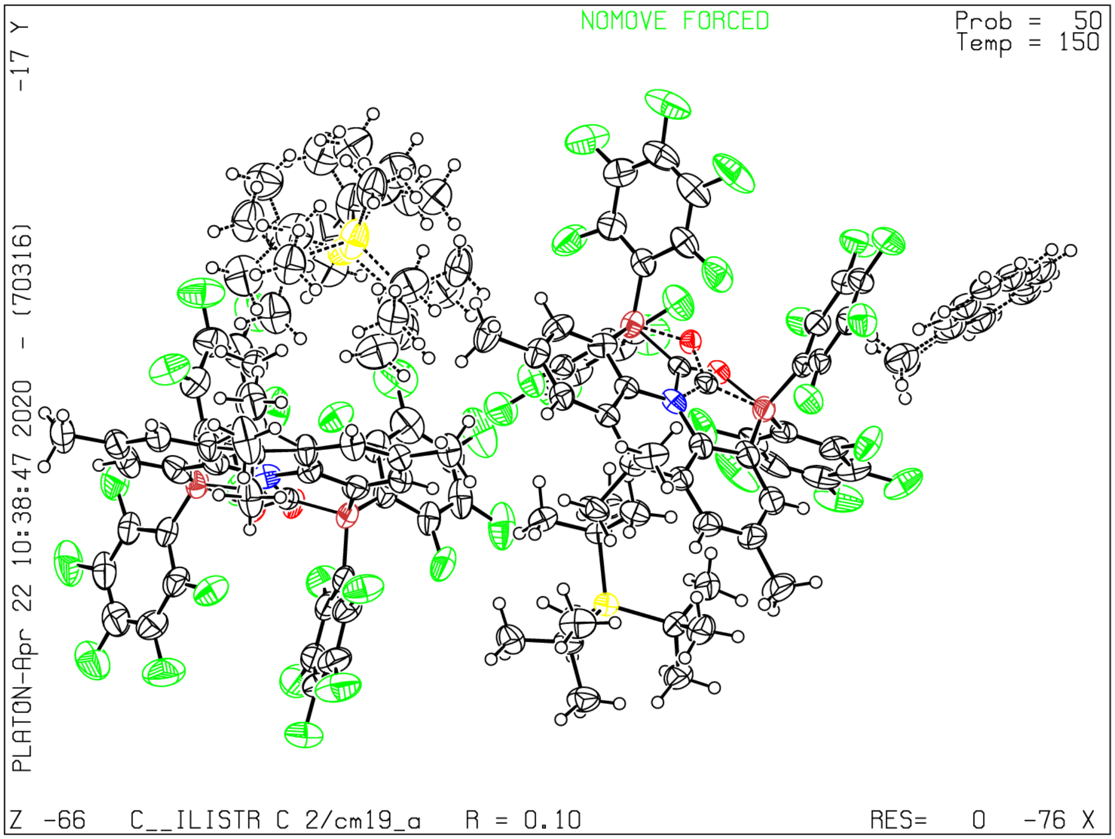

Supplement: Supplementary file 1 — Supplementary [file ANIE-60-16416-s001.zip › 9_checkcif.pdf]
