# Supplementary material for: Colorimetric Metal‐Free Detection of Carbon Monoxide: Reversible CO Uptake by a BNB Frustrated Lewis Pair
Source: Angew Chem Int Ed Engl. 2021 Jun 22;60(30):16416–9. doi: 10.1002/anie.202106413 (PMC8362209; doi:10.1002/anie.202106413)
Supplement: Supplementary file 2 — Supplementary [file ANIE-60-16416-s002.pdf]

Angewandte Chemie
Eine Zeitschrift der Gesellschaft Deutscher Chemiker

Supporting Information

Colorimetric Metal-Free Detection of Carbon Monoxide: Reversible CO Uptake by a BNB Frustrated Lewis Pair

*Xiongfei Zheng, Ili Zulkifly, Andreas Heilmann, Caitilín McManus, and Simon Aldridge**

anie_202106413_sm_miscellaneous_information.pdf

Supporting Information

Table of Contents

1. General considerations and starting material preparations s2
2. Synthetic, spectroscopic and analytical data for compounds **2**-**9** s3
3. NMR spectra of compounds **2**-**9** s10
4. X-ray crystallographic studies s29
5. Computational studies s31
6. References s33

1. General considerations and starting material preparations

All manipulations were carried out using standard Schlenk line or dry-box techniques under an atmosphere of argon or dinitrogen. Solvents were degassed by sparging with argon and dried by passing through a column of the appropriate drying agent. NMR spectra were measured in benzene-d_6 (which was dried over potassium), with the solvent then being distilled under reduced pressure and stored under argon in Teflon valve ampoules. NMR samples were prepared under argon in 5 mm Wilmad 507-PP tubes fitted with J. Young Teflon valves. ^{1}H, $^{31}P\{^{1}H\}$, $^{13}C\{^{1}H\}$, $^{11}B\{^{1}H\}$, ^{19}F NMR spectra were measured on a Bruker Avance III HD nanobay 400 MHz or Bruker Avance 500 MHz spectrometer at ambient temperature and referenced internally to residual protio-solvent (^{1}H) or solvent (^{13}C) resonances and are reported relative to tetramethylsilane (δ = 0 ppm). Assignments were confirmed using two-dimensional ^{1}H-^{1}H and ^{13}C-^{1}H NMR correlation experiments. Chemical shifts are quoted in δ (ppm) and coupling constants in Hz. Elemental analyses were carried out by Elemental Microanalysis Ltd, Okehampton, Devon, UK. UV-Vis samples were prepared by dissolving the compound in chloroform (0.8 mM) and transferring the solution into a cuvette (l = 1.0 mm) fitted with a Teflon valve. UV-Vis spectra were measured on a Perkin Elmer Lambda 19 UV/vis/NIR spectrometer. Compound 1-H[s1], KCH_2Ph[s2], $ClBAr^{f}_{2}$[s3] and $ClB(C_6F_5)_2$[s4] were synthesized according to literature. All other reagents were used as received.

2. Synthetic, spectroscopic and analytical data

Synthesis of 1

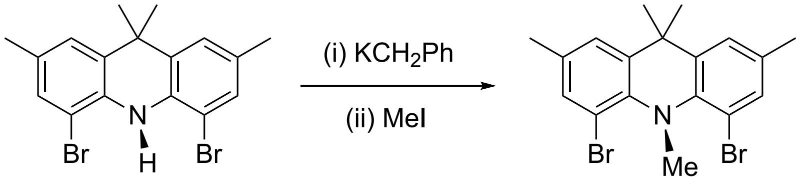

1

Dibromoacridin (2.0 g, 5.1 mmol) and KCH_2Ph (0.69 g, 5.4 mmol) were dissolved in toluene (20 mL) at 0 °C, and the reaction mixture slowly warmed to room temperature with stirring. After 4 h, MeI (0.34 ml, 5.4 mmol) was added and the reaction mixture stirred overnight. The solution was filtered and the filtrate dried under vacuum, affording pale yellow power. Yield: 1.98 g, 95.6 %.

^{1}H NMR (400 MHz, C_6D_6, 298 K): δ_H 1.17 (s, 3H, CMe_2 of backbone), 1.46 (s, 3H, CMe_2 of backbone), 1.97 (s, 6H, Ar Me), 3.50 (s, 3H, NMe), 6.91 (m, 2H, Ar CH), 7.23 (m, 2H, Ar CH). ^{13}C{^{1}H} (126 MHz, C_6D_6, 298 K): δ_C 20. 6 (Ar Me), 23.8 (C*Me$_2$* of backbone), 36.1 (C*Me$_2$* of backbone), 38.0 (CMe$_2$ of backbone), 44.4 (NMe), 118.3, 123.8, 132.5, 134.5, 143.3, 143.5.

Synthesis of 2

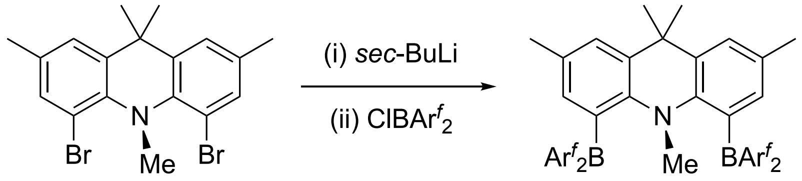

1 **2**

To a suspension of **1** (0.36 g, 0.88 mmol) in hexane (20 mL) at -78 °C was added *sec*-BuLi (1.29 mL of a 1.4 M solution in cyclohexane, 1.8 mmol). The reaction mixture was slowly warmed to room temperature and stirred for 4 h. After cooling back to 78 °C, a solution of $ClBAr^f_2$ (0.67 g, 1.88 mmol) in toluene (10 mL) was then added dropwise, during which time the colour of the solution turned to red orange. After warming to room temperature and stirring overnight, the reaction mixture was allowed to settle and filtered by cannula. The resulting filtrate was dried under vacuum and deep red orange solid was obtained. Yield: 0.85 g, 86.0 %. **2** was further purified by heating the crystals of compound **4**, giving a orange crystalline powder.

Anal. Calc. for $C_{56}H_{37}B_2F_{24}N_1$: C 53.46 %, H 2.78 %, N 1.25 %; Meas.: C 52.77 %, H 2.75 %, N 1.39 %.

^{1}H NMR (400 MHz, C_6D_6, 298 K): δ_H 1.55 (s, 6H, CMe_2 of backbone), 2.06 (s, 6H, Ar Me), 2.54 (3H, NMe), 6.66 (d, 2H, J_{HH} = 2.0 Hz, Ar CH), 7.36 (d, 2H, J_{HH} = 2.0, Ar CH), 7.76 (m, 8H, *o*-CH of Ar^f), 7.84 (m, 4H, *p*-CH of Ar^f). ^{13}C{^{1}H} (126 MHz, C_6D_6, 298 K): δ_C 20.6 (Ar Me), 29.3 (C*Me$_2$* of backbone), 36.2 (CMe$_2$ of backbone), 46.6 (NMe), 123.6 (q, $^1J_{CF}$ = 272.6 Hz, CF_3), 125.8 (*p*-CH of Ar^f), 128.4, 128.6, 130.8, 131.3, 131.8 (q, $^2J_{CF}$ = 33.3 Hz, *m*-C of Ar^f), 132.7, 137.5 (*o*-CH of Ar^f), 143.1, 145.8. ^{19}F NMR (471 MHz, C_6D_6, 298 K): δ_F -62.8. ^{11}B{^{1}H} NMR (128 MHz, C_6D_6, 25 °C): δ_B 60.7 (broad). UV-vis (chloroform, λ_{max}): 464 nm (ε = 29,600 L mol^{-1} cm^{-1}).

Synthesis of 3

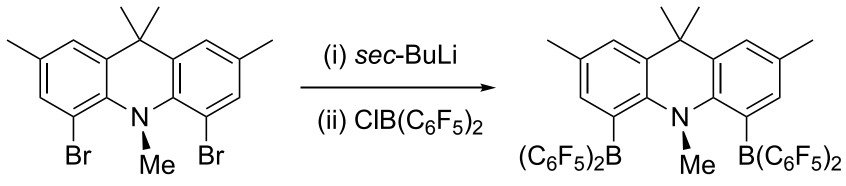

1 **3**

3 was synthesized in a manner similar to **2** using $ClB(C_6F_5)_2$ instead of $ClBAr^f_2$. To a suspension of **1** (0.39 g, 0.93 mmol) in hexane (20 mL) at -78 °C was added *sec*-BuLi (1.39 mL of a 1.4 M solution in cyclohexane, 1.86 mmol). The reaction mixture was slowly warmed to room temperature and stirred for 4 h. After cooling back to -78 °C a solution of $ClB(C_6F_5)_2$ (0.71 g, 1.86 mmol) in hexane (15 mL) was then added, during which the colour of the solution turned to red purple. After warming to room temperature and stirring overnight, the reaction mixture was allowed to settle and filtered by cannula. The resulting filtrate was dried under vacuum and deep red orange solid was obtained. Yield: 0.80 g, 89.8 %.

Although we were unable to obtain crystalline samples of **3** suitable for microanalysis (samples are typically oily), we were able to exploit the synthesis of the derived $ONNP^tBu_3$ complex (see below) to isolate a crystalline bulk sample for proof of bulk composition (including microanalysis).

^{1}H NMR (400 MHz, C_6D_6, 298 K): δ_H 1.45 (s, 6H, CMe_2 of backbone), 2.14 (s, 6H, Ar Me), 3.53 (s, 3H, NMe), 6.96 (d, 2H, J_{HH} = 1.9, Ar CH), 7.31 (d, 2H, J_{HH} = 2.0, Ar CH). ^{13}C{^{1}H} (126 MHz, C_6D_6, 298 K): δ_C 20.7 (Ar Me), 27.3 (C*Me₂* of backbone), 30.7 (C*Me₂* of backbone), 36.4 (*C*Me₂ of backbone), 48.0 (NMe), 114.8, 130.4, 130.6, 131.3, 131.4, 137.7 (d, $^1J_{CF}$ = 252.3 Hz, CF), 143.8 (d, $^1J_{CF}$ = 262.9 Hz, CF), 146.9 (*i*-C of C_6F_5), 148.2 (d, $^1J_{CF}$ = 248.7 Hz, CF). ^{11}B{^{1}H} NMR (128 MHz, C_6D_6, 25 °C): δ_B 61.0 (broad). ^{19}F NMR (471 MHz, C_6D_6, 298 K): δ_F -128.4 (m, 8F, *o*-CF), -145.2 (m, 4F, *p*-CF), -160.4 (m, 8F, *m*-CF). UV-vis (chloroform, λ_{max}): 500 nm (ε = 43,525 L mol^{-1} cm^{-1}).

Synthesis of 3·ONNP^{t}Bu$_3$

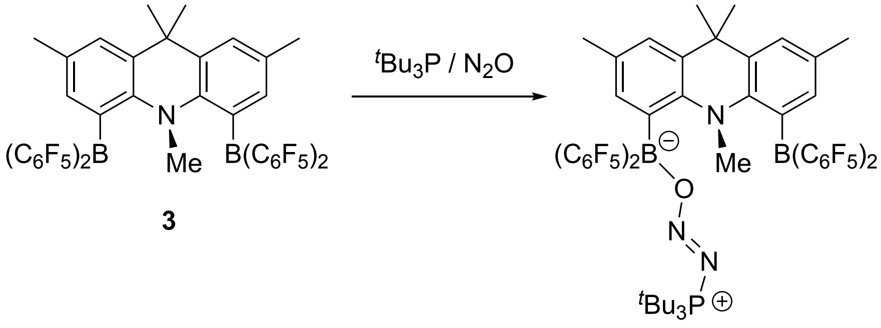

3·ONNP^{t}Bu$_3$

3 (150 mg, 0.37 mmol) and ^{t}Bu$_3$P (74 mg, 0.37 mmol) were dissolved in benzene (2 mL). The resulting solution was subjected to three freeze-pump-thaw cycles before backfilling with N_2O (ca. 1 atm). The solution was allowed to stand at room temperature for 18 h, yielding colourless crystals, which were

separated by filtration, washed with pentane (3 x 2 mL) and dried in vacuum to afford the adduct **3**·ONNP^{t}Bu$_3$ as colourless crystals. Yield: 0.13 g, 31 %.

Anal. Calc. for $C_{54}H_{46}B_2F_{20}N_3OP$: C 54.71 %, H 3.91 %, N 3.54 %; Meas.: C 54.31 %, H 4.28 %, N 3.18 %.

^{1}H NMR (400 MHz, C_6D_6, 298 K): δ_H 1.26 (d, J_{PH} = 9.3 Hz, 18H, P^{t}Bu$_3$), 1.40 (s, 6H, C*Me*$_2$ of backbone), 2.14 (s, 6H, Ar Me), 3.53 (s, 3H, NMe), 6.96 (s, 2H, Ar CH), 7.31 (s, 2H, Ar CH). ^{11}B{^{1}H} NMR (128 MHz, C_6D_6, 298 K): δ_B 5.2 (B(C_6F_5)$_2$ONNP^{t}Bu$_3$), 62.0 (B(C_6F_5)$_2$). ^{13}C NMR (101 MHz, CD_2Cl_2, 298 K): δ_C 21.5 (Ar Me), 21.8 (Ar Me), 25.6 (C$\underline{Me}_2$ of backbone), 29.7 (PC$\underline{Me}_3$), 30.6 (C$\underline{Me}_2$ of backbone), 31.2 ($\underline{C}$Me$_2$ of backbone), 41.7 (d, J_{CP} = 25.6 Hz, P$\underline{C}$Me$_3$), 42.1 (NMe), 123.3, 127.5, 130.6, 131.7, 136.3 (d, J_{CF} = 240.3 Hz, CF), 137.1, 137.2, 138.8, 139.8, 140.9, 141.8 (d, J_{CF} = 297.5 Hz, CF), 149.5 (d, J_{CF} = 275.4 Hz, CF). ^{19}F NMR (377 MHz, C_6D_6, 298 K): δ_F -128.4 (m, 4F, *o*-CF), -132.4 (dd, J_{FF} = 24.5, 8.9Hz, 4F, *o*-CF), -148.1 (t, J_{FF} = 18.1 Hz, 2F, *p*-CF), -159.5 (t, J_{FF} = 18.6 Hz, 2F, *p*-CF), -160.5 (m, 4F, *m*-CF), -165.7 (m, 4F, *m*-CF). ^{31}P{^{1}H} NMR (162 MHz, C_6D_6, 298 K): δ_P 66.5.

Synthesis of 4

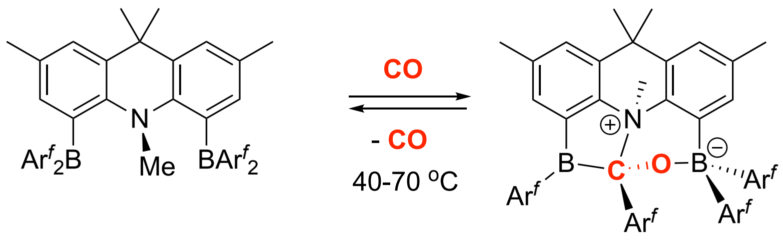

2 **4**

2 (30 mg, 0.027 mmol) was dissolved in hexane (2 mL) and the solution degassed by the freeze-pump-thaw method, before CO was admitted (ca. 1 atm), at which point the product precipitated from the solution. The reaction mixture was kept stand for 10 min before the solvent was decanted. The obtained white crystalline solid was then washed twice with cold hexane (2 x 1 ml) and dried under vacuum. Crystals which were qualified for X-ray diffraction was obtained by dissolving **2** in toluene in NMR tube and charged with 1 atm CO. Yield: 27 mg, 87.8 %.

Satisfactory microanalytical data were obtained by synthesizing the DMAP adduct of **4** (i.e. **7**, see below).

^{1}H NMR (400 MHz, $CDCl_3$, 298 K): δ_H 1.86 (s, 3H, CMe$_2$ of backbone), 2.02 (s, 3H, CMe$_2$ of backbone), 2.51 (s, 3H, Ar Me), 2.59 (s, 3H, Ar Me), 3.58 (s, 3H, NMe), 6.76 (s, 2H, Ar CH), 7.11 (s, 2H, Ar CH), 7.15 (s, 1H, Ar CH), 7.36 (s, 1H, Ar CH), 7.42 (s, 1H, Ar CH), 7.53 (s, 1H, Ar CH), 7.65 (s, 1H, Ar CH), 7.87 (s, 1H, Ar CH), 7.92 (s, 1H, Ar CH), 8.31 (overlapping s, 3H, Ar CH), 9.36 (s, 2H, Ar CH). ^{11}B{^{1}H} NMR (128 MHz, $CDCl_3$, 25 °C): δ_B 31.9 (broad), -0.5. ^{13}C{^{1}H} (126 MHz, $CDCl_3$, 298 K): δ_C 22.1 (Ar Me), 29.4 (C$\underline{Me}_2$ of backbone), 35.4 (C$\underline{Me}_2$ of backbone), 37.2 ($\underline{C}$Me$_2$ of backbone), 69.6 (NMe), 116.4, 119.2, 121.8, 122.2 (q, $^1J_{CF}$ = 272.1 Hz, CF_3), 123.1 (q, $^1J_{CF}$ = 273.4 Hz, CF_3), 123.9 (q, $^1J_{CF}$ = 272. 5Hz, CF_3), 124.1 (q, $^1J_{CF}$ = 272.1 Hz, CF_3), 125.3, 129.1, 129.5 (q, $^2J_{CF}$ = 32.1 Hz, *m*-C), 130.1 (q, $^2J_{CF}$ = 32.1 Hz, *m*-C), 130.7 (q, $^2J_{CF}$ = 34.1 Hz, *m*-C), 131.0, 131.5, 132.4, 132.8 (q, $^2J_{CF}$ = 33.8 Hz, *m*-C), 134.4, 135.4, 135.9, 136.1, 136.2, 136.3, 138.1, 139.8, 142.5, 148.6, 160.0 (br, NCO). ^{19}F NMR (471 MHz, $CDCl_3$, 298 K): δ_F -62.6 (s, 6F), -63.1 (s, 6F), -63.5 (s, 6F), -63.6 (s, 6F).

Synthesis of 5

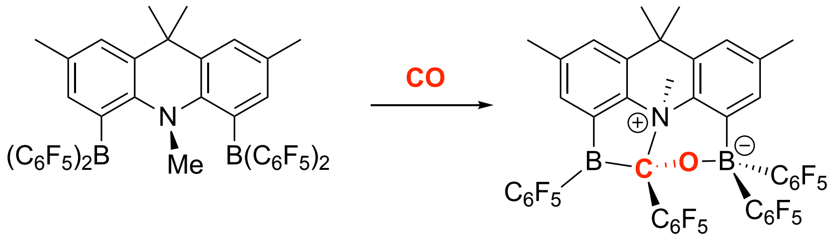

3 **5**

3 (45mg, 0.048 mmol) was dissolved in 2 ml of hexane, the resulting solution was processed with three times freeze-pump-thaw and backfilled with 1 atm CO at -40 °C. The supernatant was decanted and the white powder was further washed with cold hexane (2 x 2 ml) and dried under vacuum. Yield: 10 mg, 22 %.

^{1}H NMR (400 MHz, C_6D_6, 298K): δ = 1.28 (s, 3H, CMe_2 of backbone), 1.32 (s, 3H, CMe_2 of backbone). 2.02 (s, 3H, Ar Me), 2.08 (s, 3H , Ar Me), 2.84 (s, 3H, NMe), 6.98 (m ,1H, Ar CH), 7.10 (m, 1H, Ar CH), 7.23(s, 1H, Ar CH), 7.68 (s, 1H Ar CH). ^{13}C{^{1}H} (126 MHz, $CDCl_3$, 298 K): δ_C 21.2 (ArMe), 21.6 (ArMe), 30.9 (C*Me₂* of backbone), 34.0 (C*Me₂* of backbone), 37.1 (CMe_2 of backbone), 61.2 (NMe), 127.2, 127.5, 129.1, 133.3, 133.8, 134.2, 134.5, 136.5, 136.8 (d, $^{1}J_{CF}$ = 250.5 Hz, CF), 140.2, 140.7, 142.0, 145.9, 147.9 (d, $^{1}J_{CF}$ = 243.5 Hz, CF), 148.2 (d, $^{1}J_{CF}$ = 246.3 Hz, CF), 148.7 (d, $^{1}J_{CF}$ = 248.6 Hz, CF), OCNMe not observed . ^{11}B{^{1}H} NMR (128 MHz, C_6D_6, 25 °C): δ_B 25.4 (broad), -15.0. ^{19}F{^{1}H} NMR (471 MHz, C_6D_6, 298 K): 130.1 (s, 4F, *o*-F of Ar^F), -131.8 (s, 2F, *o*-F of Ar^F), -135.7 (m, 2F, *o*-F of Ar^F), -151.9 (t, 1F, J_{FF} = 20.1 Hz, *p*-F of Ar^F), -153.1 (t, 1F, J_{FF} = 21.3 Hz, *p*-F of Ar^F), -155.1 (t, 1F, J_{FF} = 21.1 Hz, *p*-F of Ar^F), -155.5 (t, 1F, J_{FF} = 20.9 Hz, *p*-F of Ar^F), -161.8 (m, 2F, *m*-F of Ar^F), -162.0 (m, 2F, *m*-F of Ar^F), -162.3 (m, 2F, *m*-F of Ar^F), -162.9 (m, 2F, *m*-F of Ar^F).

Synthesis of 6

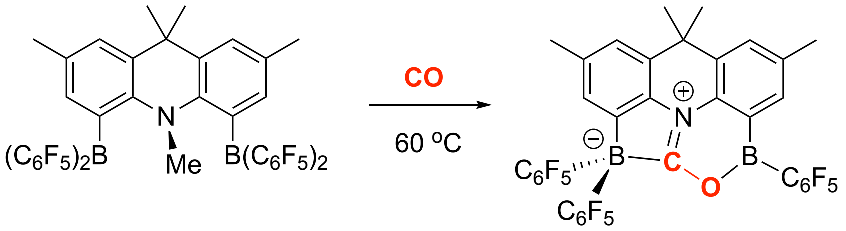

3 **6**

3 (50 mg, 0.053 mmol) was dissolved in pentane (2 mL) and the solution degassed by the freeze-pump-thaw method, before CO was admitted (ca. 1 atm). The reaction mixture was heated at 60 °C for 10 min, cooled to room temperature and filtered by cannula. The resulting filtrate was concentrated to 5 ml and cooled to 4 °C for crystallization. **6** was obtained as a colourless crystalline material, which was washed twice with cold pentane (2 x 1 mL) and dried under vacuum. Crystals which were qualified for XRD was obtained by slow-evaporating a pentane solution of **6.** Yield: 34 mg, 81.3 %.

Anal. Calc. for $C_{36}H_{16}B_2F_{15}N_1O_1$: C 55.07 %, H 2.05 %, N 1.78 %: Meas.: C 55.40 %, H 2.26 %, N 1.85 %.

^{1}H NMR (400 MHz, $CDCl_3$, 298 K): δ_H 1.82 (s, 6H, CMe_2 of backbone), 2.44 (s, 3H, Ar Me), 2.61 (s, 3H, Ar Me), 7.21 (s, 1H, Ar CH), 7.51 (s, 1H, Ar CH), 7.83 (s, 1H, Ar CH), 8.00 (s, 1H, Ar CH). ^{11}B{^{1}H} (128 MHz, $CDCl_3$, 25 ºC): δ_B 37.2 (broad), -14.5. ^{13}C{^{1}H} (126 MHz, $CDCl_3$, 298 K): δ_C 22.1 (Ar Me), 22.2 (Ar Me), 34.2 (C$\underline{Me_2}$ of backbone), 36.9 ($\underline{C}$Me$_2$ of backbone), 124.7, 130.4, 131.6, 132.3, 133.1, 135.3, 136.7, 137.2 (d, $^1J_{CF}$ = 238.1 Hz, CF), 137.7, 138.4 (br), 139.9, 141.0, 146.8 (br), 148.5 (d, $^1J_{CF}$ = 260.6 Hz, CF) [O*C*=NMe not observed]. ^{19}F NMR (471 MHz, $CDCl_3$, 298 K): δ_F -129.3 (m, 2F, *o*-F of BC_6F_5), -130.9 (m, 4F, *o*-F of $B(C_6F_5)_2$), -146.6 (m, 1F, *p*-F of BC_6F_5), -158.6 (m, 2F, *p*-F of $B(C_6F_5)_2$), -159.5 (m, 2F, *m*-F of BC_6F_5), -163.7 (m, 4F, *m*-F of $B(C_6F_5)_2$).

Synthesis of 7

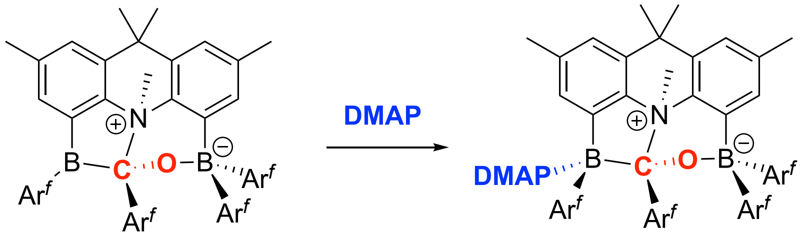

4 **7**

4 (124 mg, 0.11 mmol) and DMAP (13 mg, 0.11 mmol) were dissolved in chloroform (1 mL) and layered with pentane. The crystals of **7** could be obtained after few days. The crystals was then isolated, washed with benzene (2 x 1 ml) and pentane (2 x 1 ml), and dried under vacuum. Yield: 45 mg, 32.8 %.

Anal. Calc. for $C_{58}H_{41}B_2F_{24}N_3O_1$: C 54.70 %, H 3.24 %, N 3.30 %; Meas.: C 54.34 %, H 3.16 %, N 3.30 %.

^{1}H NMR (400 MHz, C_6D_6, 298K): δ = 1.75 (s, 3H, CMe_2 of backbone), 2.00 (s, 3H, CMe_2 of backbone), 2.37 (s, 3H, Ar Me), 2.46 (s, 3H, Ar Me), 3.03 (s, 3H, NMe), 3.24 (s, 3H, NMe_2 of DMAP), 3.40 (s, 3H, NMe_2 of DMAP), 6.26 (s, 1H, Ar CH), 6.45 (s, 2H, Ar CH), 6.57 (s, 1H, Ar CH), 6.66 (dd, 1H, J_{HH} = 7.29, 3.18 Hz, Ar CH), 6.87 (s, 2H, Ar CH), 6.94 (s, 1H, Ar CH), 7.09 (s, 1H, Ar CH), 7.21 (s, 1H, Ar CH), 7.30 (m, 3H, Ar CH), 7.40 (dd, 1H, J_{HH} = 7.29, 3.18 Hz, Ar CH), 7.51 (s, 1H, Ar CH), 8.09 (dd, 1H, J_{HH} = 7.34, 1.26 Hz, Ar CH), 8.21 (s, 2H, Ar CH), 10.23 (dd, 1H, J_{HH} = 7.34, 1.26 Hz, Ar CH). ^{13}C{^{1}H} (126 MHz, $CDCl_3$, 298 K): δ_C 21.9 (Ar Me), 22.0 (Ar Me), 29.9 (C$\underline{Me_2}$ of backbone), 35.7 (C$\underline{Me_2}$ of backbone), 36.8 ($\underline{C}$Me$_2$ of backbone), 39.9 (NMe_2 of DMAP), 61.0 (NMe), 107.4, 108.0, 118.9, 119.5, 119.7, 120.0 (q, $^1J_{CF}$ = 272.6 Hz, CF_3), 120.4 (q, $^1J_{CF}$ = 272.6 Hz, CF_3), 120.9 (q, $^1J_{CF}$ = 270.8 Hz, CF_3), 121.0, 121.5 (q, $^1J_{CF}$ = 272.6 Hz, CF_3), 123.2, 125.0, 125.4, 126.3, 127.5 (q, $^2J_{CF}$ = 33.4 Hz, *m*-C), 127.9, 128.2 (q, $^2J_{CF}$ = 33.2 Hz, *m*-C), 128.4 (q, $^2J_{CF}$ = 32.0 Hz, *m*-C), 128.5, 128.9, 129.1, 129.2 (q, $^2J_{CF}$ = 32.4 Hz, *m*-C), 129.4, 129.6, 131.7, 131.9, 133.9, 134.0, 135.2, 135.3, 135.6, 137.9, 140.2, 140.9, 143.3, 143.8, 147.1, 155.9, 160.6 (broad, NCO). ^{11}B{^{1}H} NMR (128 MHz, $CDCl_3$, 25 °C): δ_B 30.5 (broad), -0.69. ^{19}F NMR (471 MHz, $CDCl_3$, 298 K): δ = -61.8 (s, 6F), -62.8 (s, 6F), -63.0 (s, 6F), -63.1 (s, 3F), -63.7 (s, 3F).

Synthesis of 8

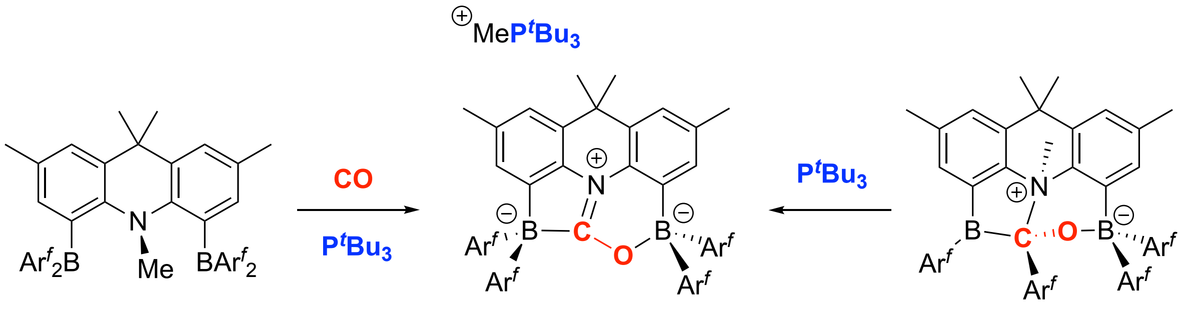

2 **8** **4**

Compound **8** can be obtained by two methods. Method A: **2** (61.0 mg, 0.054 mmol) and P^{t}Bu$_3$ (10.7 mg, 0.054 mmol) were dissolved in benzene, and the solution degassed by the freeze-pump-thaw before CO was admitted (ca. 1 atm). The reaction mixture was then stirred until the colour faded to almost colourless, upon which crystalline product precipitated from the solution which were qualified for XRD. The supernatent was then decanted off, and the product washed with cold hexane twice (2 x 5 mL) and dried. Yield: 57.1 mg, 79.6 %. Method B: **4** (99.5 mg, 0.086 mmol) and P^{t}Bu$_3$ (17.5 mg, 0.086 mmol) were suspended in benzene (2 mL), and the mixture stirred for 10 min. The solvent was then decanted, affording the product as a colourless crystalline material, which was washed with cold hexane (2 x 5 mL) and dried. Yield: 85.0 mg, 72.6 %).

Anal. Calc. for $C_{63}H_{58}B_2F_{24}NOP$: C 55.90 %, H 4.32 %, N 1.03 %; Meas.: C 55.91 %, H 4.28 %, N 1.10 %.

^{1}H NMR (400 MHz, CDCl$_3$, 298K): δ_H 1.37 (d, 27H, J_{PH} = 14.5 Hz, MeP^{t}Bu$_3$), 1.44 (d, 3H, J_{PH} = 15.9 Hz, MeP^{t}Bu$_3$), 1.72 (s, 6H, CMe$_2$ of backbone), 2.28 (s, 3H, Ar Me), 2.42 (s, 3H, Ar Me), 6.82 (d, 1H, J_{HH} = 1.46 Hz, Ar CH), 7.10 (m, 1H, Ar CH), 7.13 (m, 1H, Ar CH), 7.29 (m, 1H, Ar CH), 7.49 (s, 2H, *p*-Ar CH of BAr$^f_2$), 7.56 (s, 2H, *p*-Ar CH of BAr$^f_2$), 7.62 (s, 4H, *o*-Ar CH of BAr$^F_2$), 7.77 (s, 4H, o-Ar*H* of BAr$^f_2$). ^{11}B{^{1}H} NMR (128 MHz, CDCl$_3$, 25 °C): δ_B 1.81 (s, br, O*B*Ar$_2$), -10.2 (s, *B*Ar$_2$CO). ^{13}C{^{1}H} (126 MHz, CDCl$_3$, 298 K): δ_C 0.36 (d, $^1J_{PC}$ = 45.4 Hz, MeP^{t}Bu$_3$), 21.9 (Ar Me), 22.1 (Ar Me), 28.9 (MePCMe$_3$), 34.0 (CMe$_2$ of backbone), 36.3 (CMe$_2$ of backbone), 37.9 (d, $^1J_{PC}$ = 30.8 Hz, MePCMe$_3$), 118.6 (*p*-CH of Ar^f), 119.1 (*p*-CH of Ar^f), 123.9, 124.7 (q, $^1J_{CF}$ = 272.1 Hz, CF$_3$), 125.7, 127.2, 128.4, 129.4 (q, $^2J_{CF}$ = 32.3 Hz, *m*-C of Ar^f), 129.9, 130.9, 131.5, 132.6 (*o*-CH of Ar^f),132.9 (*o*-CH of Ar^f), 135.1, 136.4, 137.2, 157.3 (NCO). ^{19}F NMR (471 MHz, CDCl$_3$, 298 K): δ = -62.8 (s, 12F), -62.9 (s, 12F). ^{31}P{^{1}H} NMR (162 MHz, CDCl$_3$, 298 K): δ_P -49.1.

Synthesis of 9

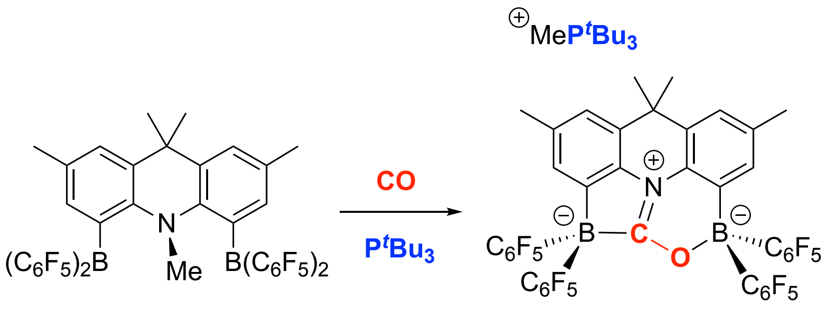

3 **9**

The synthesis of **9** was achieved via a similar method to the synthesis of **8** (via method A). **3** (16.6 mg, 0.018 mmol) and P^tBu_3 (3.6 mg, 0.018 mmol) were dissolved in benzene (1 mL), and the solution degassed by the freeze-pump-thaw method before CO was admitted (ca. 1 atm). The reaction mixture was shaken until the colour faded, at which point crystalline **9** precipitated from the solution. The supernatent was then decanted off, and the product washed with cold hexane (2 x 5 mL) and dried. Yield: 11.5 mg, 57.0%.

Anal. Calc. for $C_{55}H_{46}B_2F_{20}NOP$: C 56.48 %, H 3.96 %, N 1.20 %; Meas.: C 55.99 %, H 4.09 %, N 1.20 %.

^{1}H NMR (400 MHz, CD_2Cl_2, 298K): δ_H 1.52 (d, 27H, J_{PH} = 14.5 Hz, MeP^{t}Bu$_3$), 1.57 (d, 3H, J_{PH} = 11.5 Hz, MeP^{t}Bu$_3$), 1.68 (s, 6H, CMe$_2$ of backbone), 2.28 (s, 3H, Ar Me), 2.30 (s, 3H, Ar Me), 6.78 (m, 1H, J_{HH} = 1.46 Hz, Ar CH), 7.06 (m, 1H, Ar CH), 7.10 (m, 1H, Ar CH), 7.18 (m, 1H, Ar CH). ^{11}B NMR (128 MHz, CD_2Cl_2, 25 °C): δ_B -1.1 (s, br, O*B*Ar$_2$), -15.0 (s, *B*Ar$_2$CO). ^{13}C{^{1}H} (126 MHz, CD_2Cl_2, 298 K): δ_C 0.97 (d, $^1J_{PC}$ = 45.9 Hz, MeP^{t}Bu$_3$), 21.7 (ArMe), 21.8 (Ar Me), 29.3 (MePCMe$_3$), 33.7 (CMe$_2$ of backbone), 36.5 (*C*Me$_2$ of backbone), 38.3 (d, $^1J_{PC}$ = 31.3 Hz, MePCMe$_3$), 120.7, 122.9, 123.9, 126.0, 127.3, 128.0, 128.2, 128.4, 128.5, 128.7, 130.1, 131.1, 131.6, 135.2, 136.7, 136.8, 136.9 (d, $^1J_{CF}$ = 244.6 Hz, CF), 138.7 (d, $^1J_{CF}$ = 242.3 Hz, CF), 139.3 (d, $^1J_{CF}$ = 246.0 Hz, CF), 148.1 (d, $^1J_{CF}$ = 240.3 Hz, CF), O=CNMe not observed. ^{19}F NMR (471 MHz, CD_2Cl_2, 298 K): δ_F -131.7 (m, 4F, *o*-F of BAr$_2$), -134.2 (m, 4F, *o*-F of BAr$_2$), -161.8 (t, 2F, J_{FF} = 19.7 Hz, *p*-F of BAr$_2$), -162.6 (t, 2F, J_{FF} = 20.9 Hz, *p*-F of BAr$_2$), -166.3 (m, 8F, *m*-F of BAr$_2$). ^{31}P{^{1}H} NMR (162 MHz, CD_2Cl_2, 298 K): δ_P -49.4.

3. Representative NMR spectra

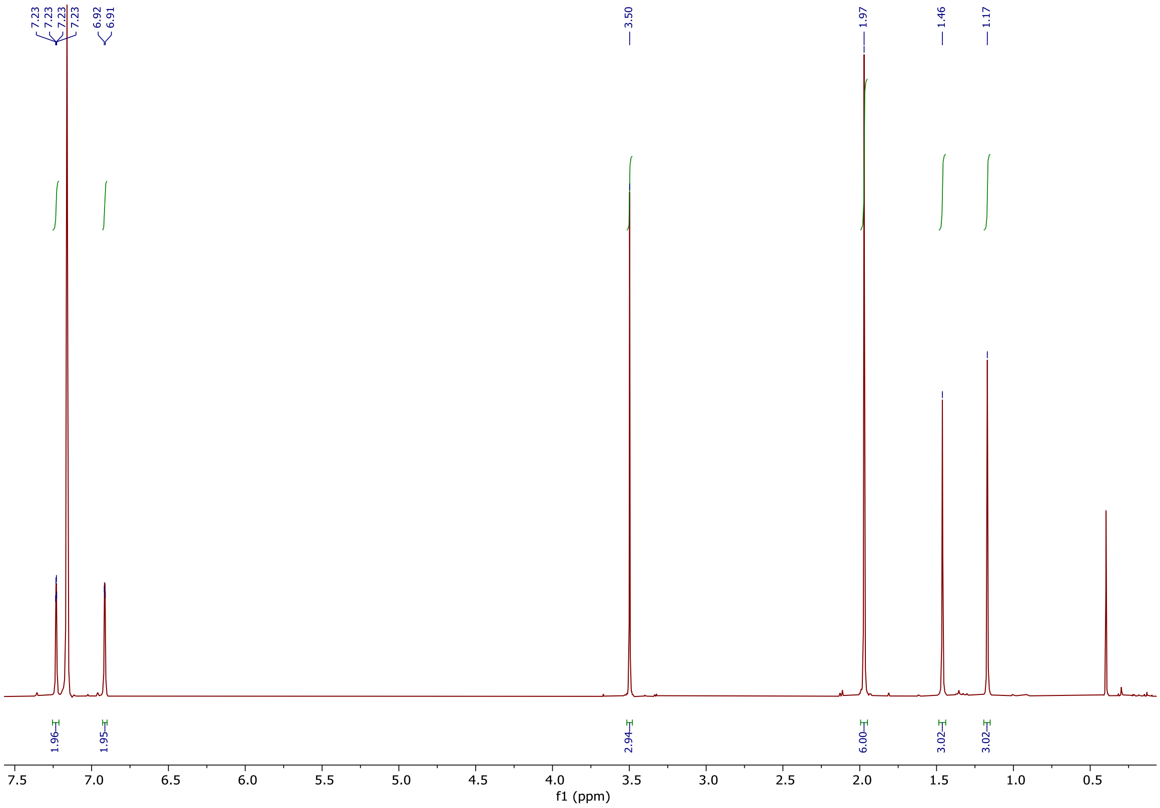

Figure S1. ^{1}H NMR spectrum of **1** in C_6D_6 at 298 K.

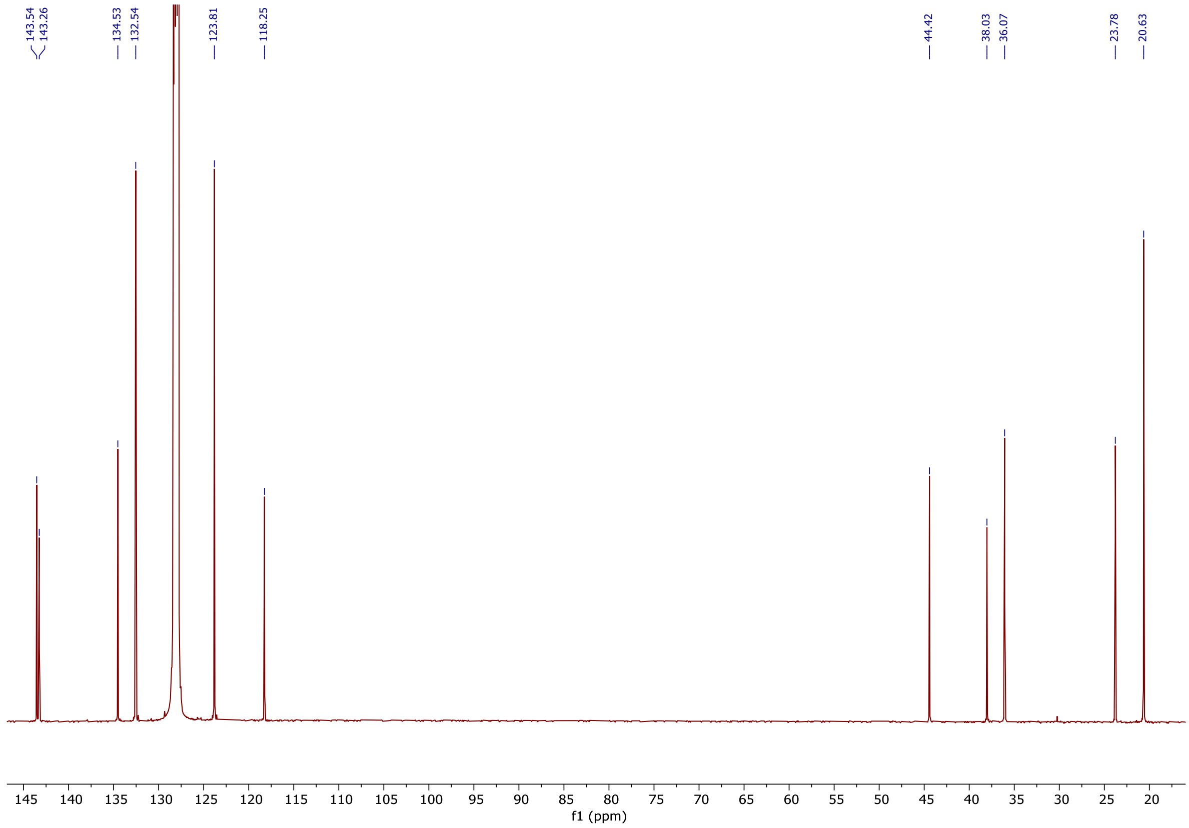

Figure S2. $^{13}C\{^{1}H\}$ NMR spectrum of **1** in C_6D_6 at 298 K.

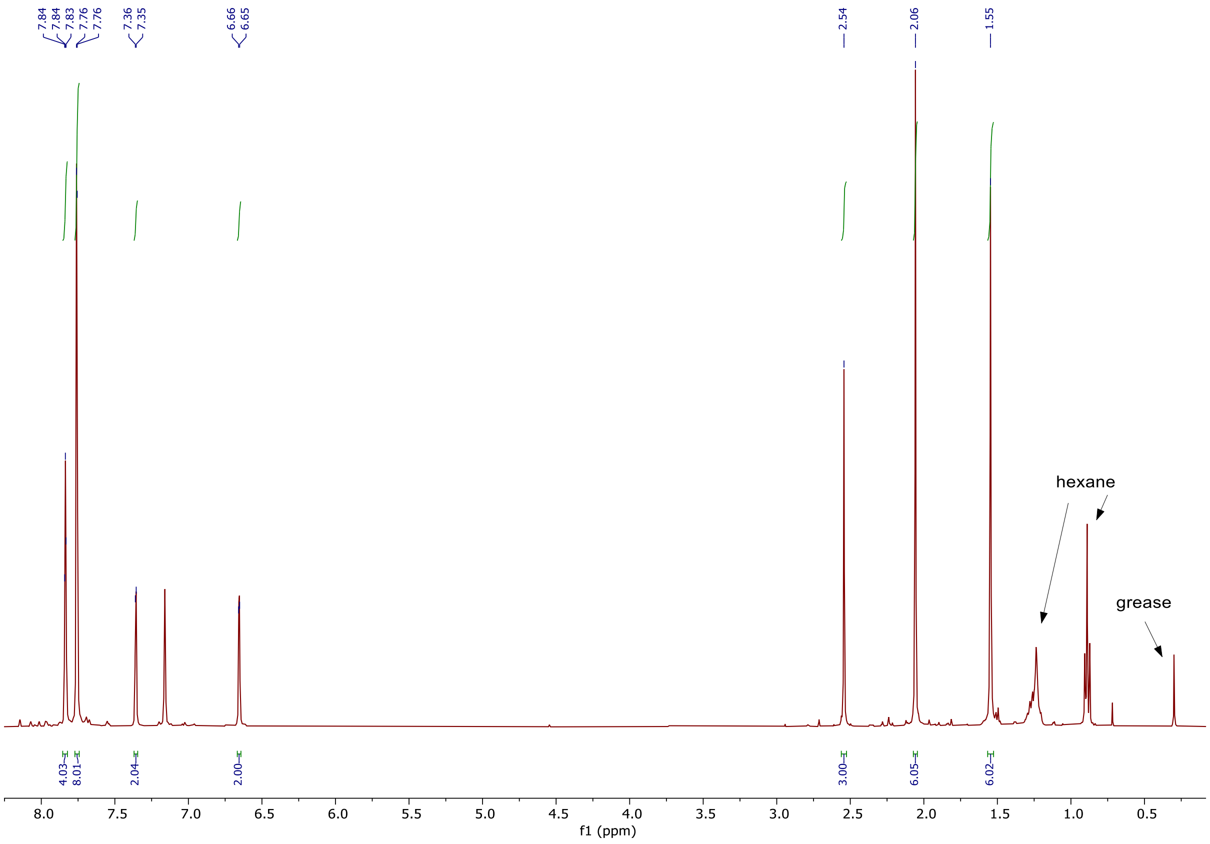

Figure S3. ^{1}H NMR spectrum of **2** in C_6D_6 at 298 K.

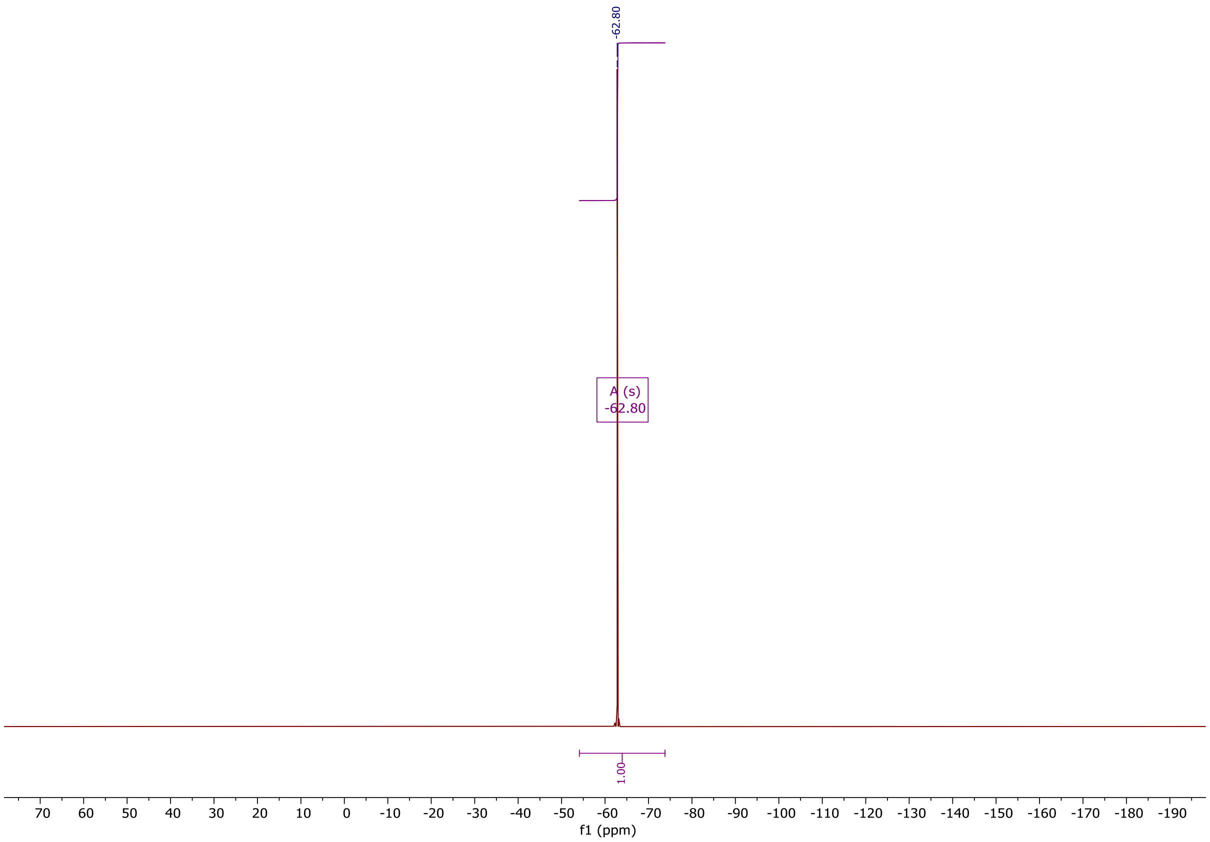

Figure S4. ^{19}F NMR spectrum of **2** in C_6D_6 at 298 K.

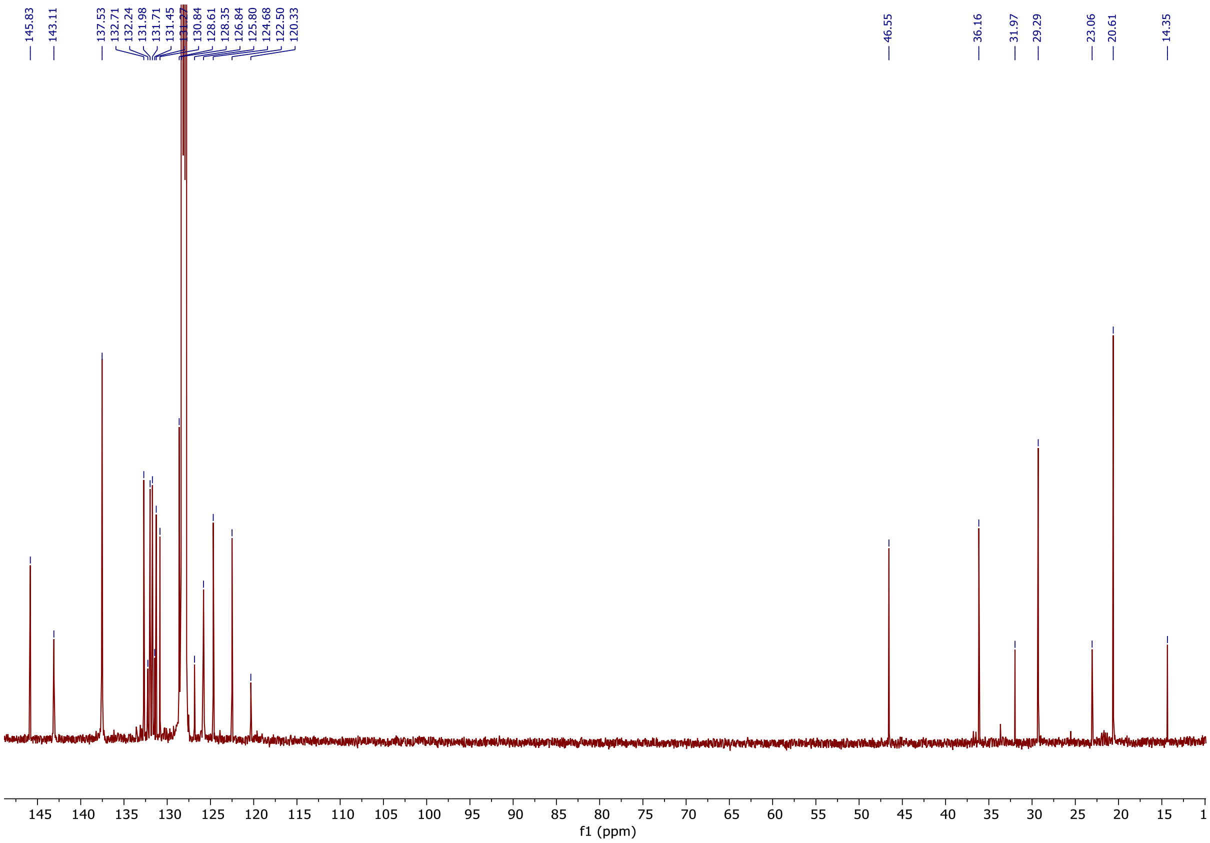

Figure S5. $^{13}C\{^{1}H\}$ NMR spectrum of **2** in C_6D_6 at 298 K.

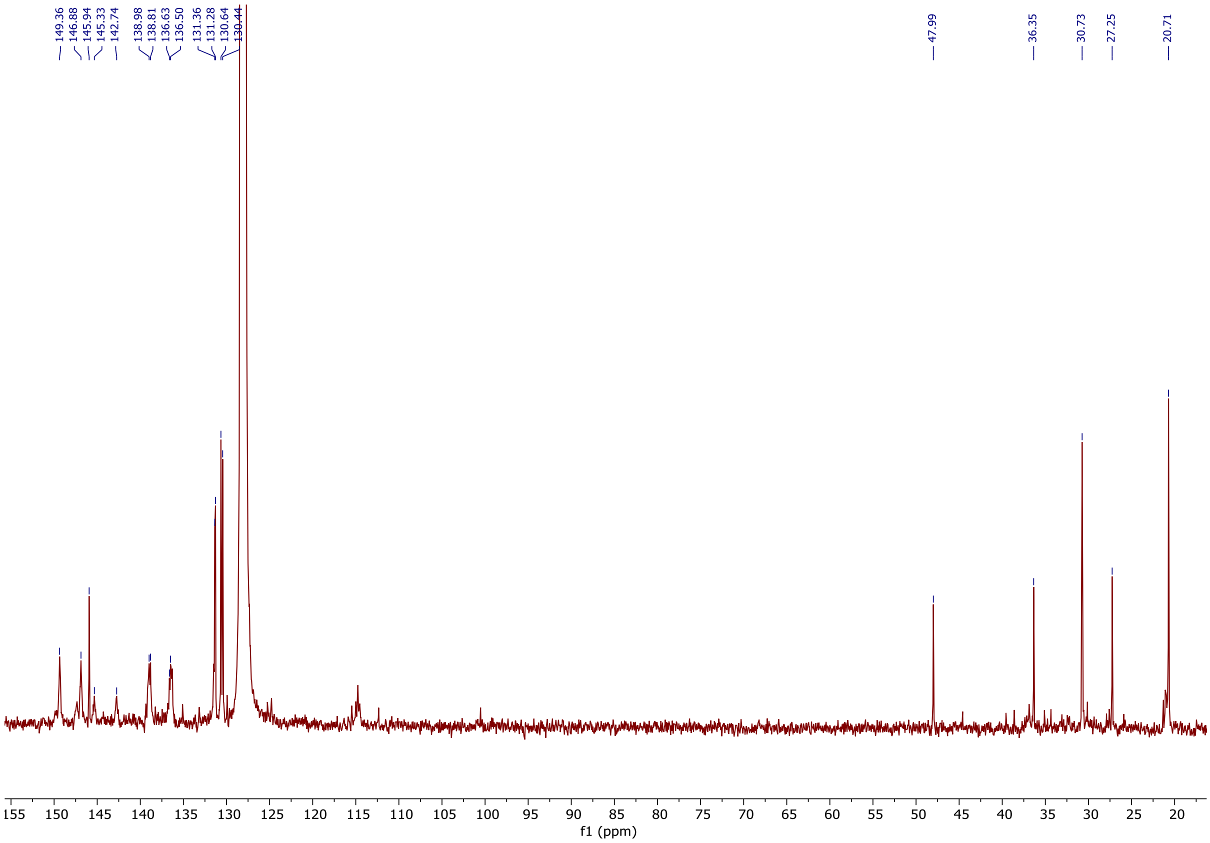

Figure S8. $^{13}C\{^1H\}$ NMR spectrum of **3** in C_6D_6 at 298 K.

Figs S6 and S7 here - ^1H and ^{19}F NMR spectra of **3**

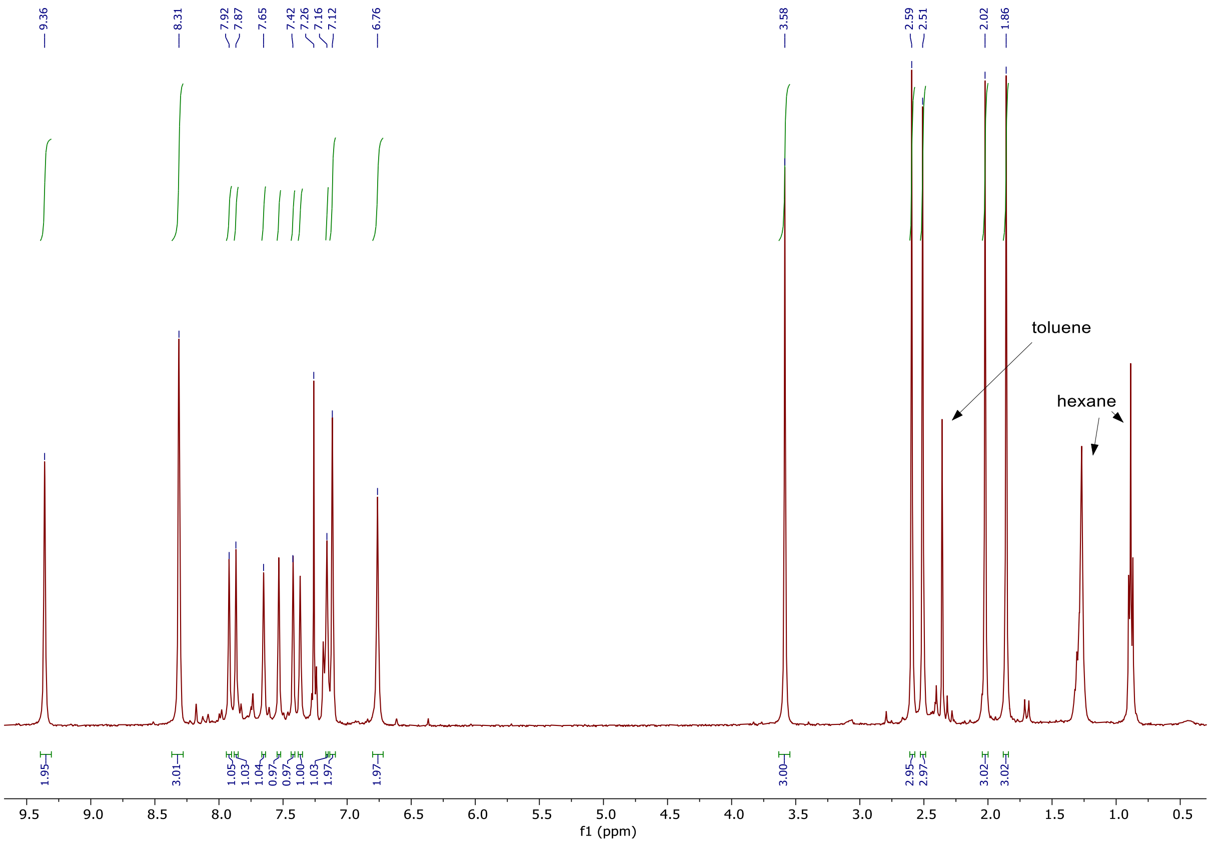

Figure S9. ^{1}H NMR spectrum of **4** in $CDCl_3$ at 298 K.

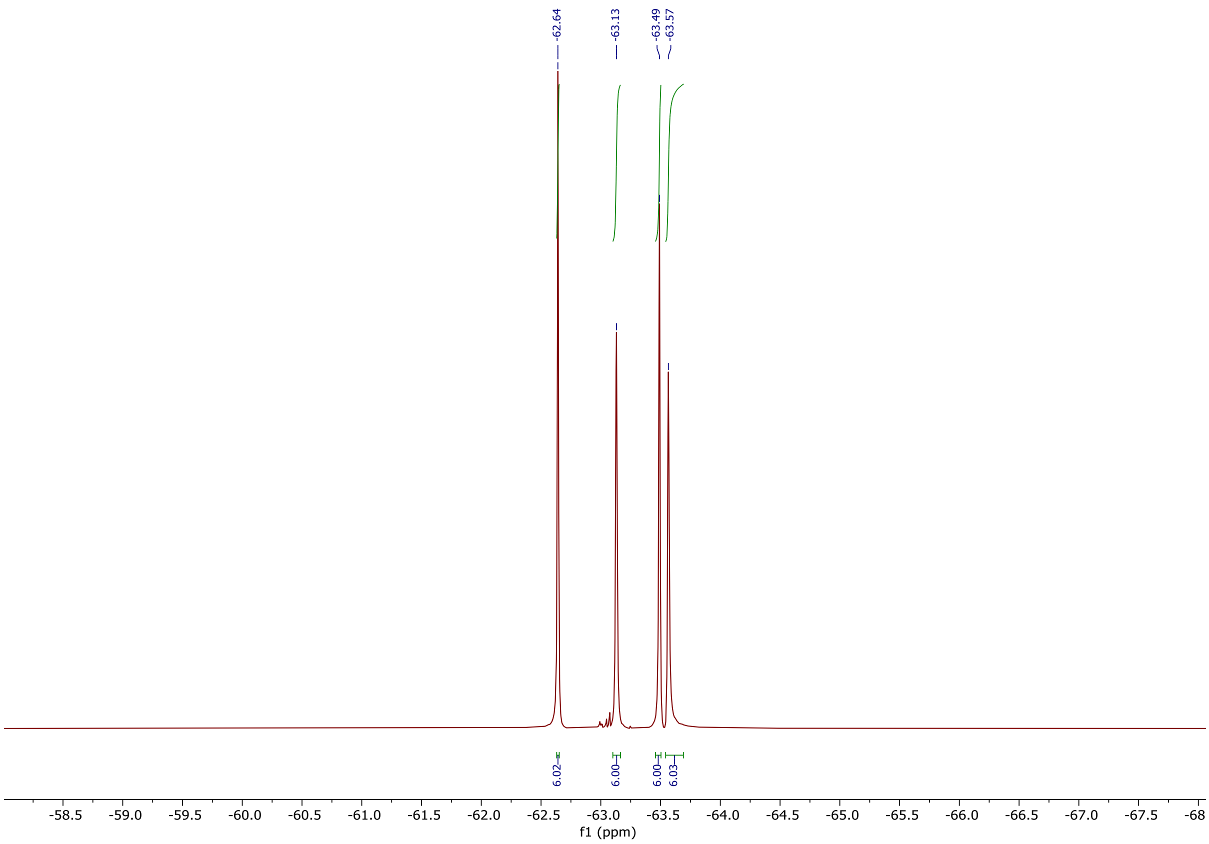

Figure S10. ^{19}F NMR spectrum of **4** in $CDCl_3$ at 298 K.

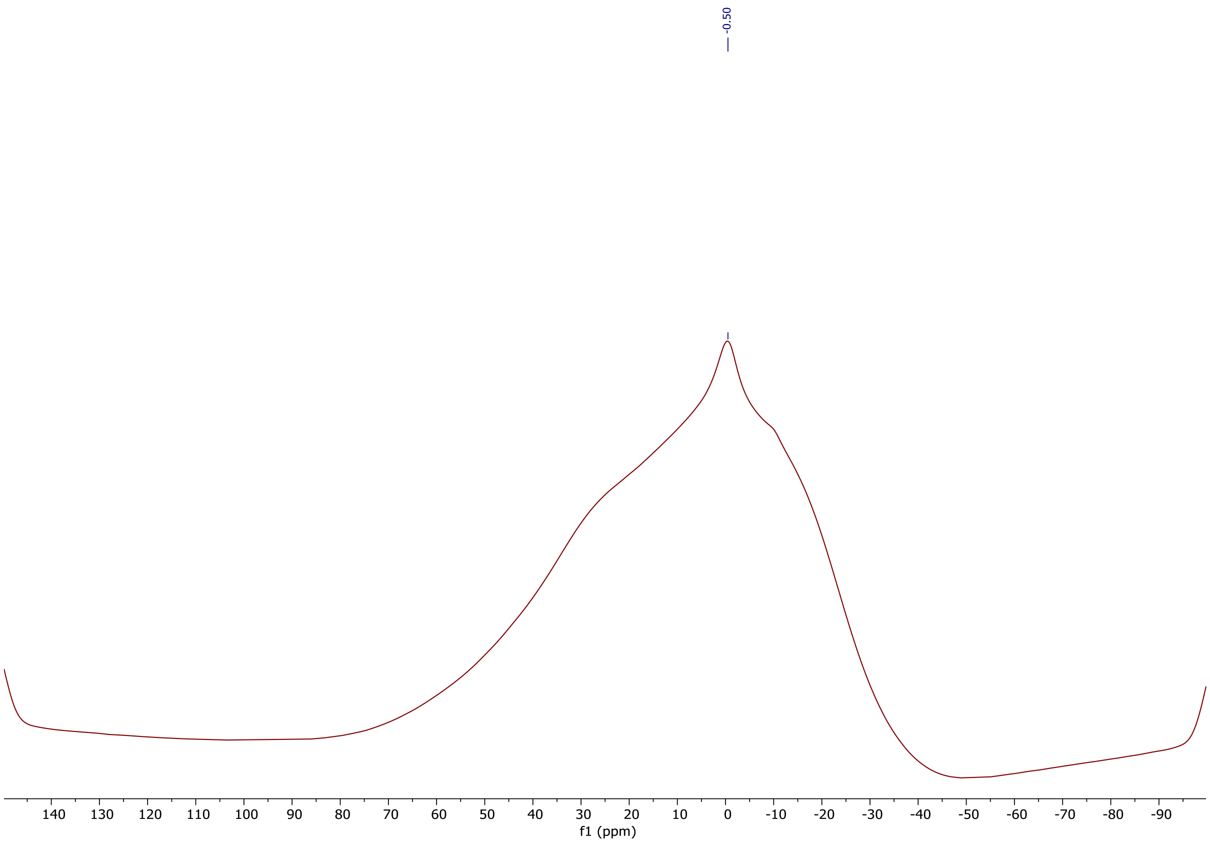

Figure S11. $^{11}B\{^{1}H\}$ NMR spectrum of **4** in $CDCl_3$ at 298 K.

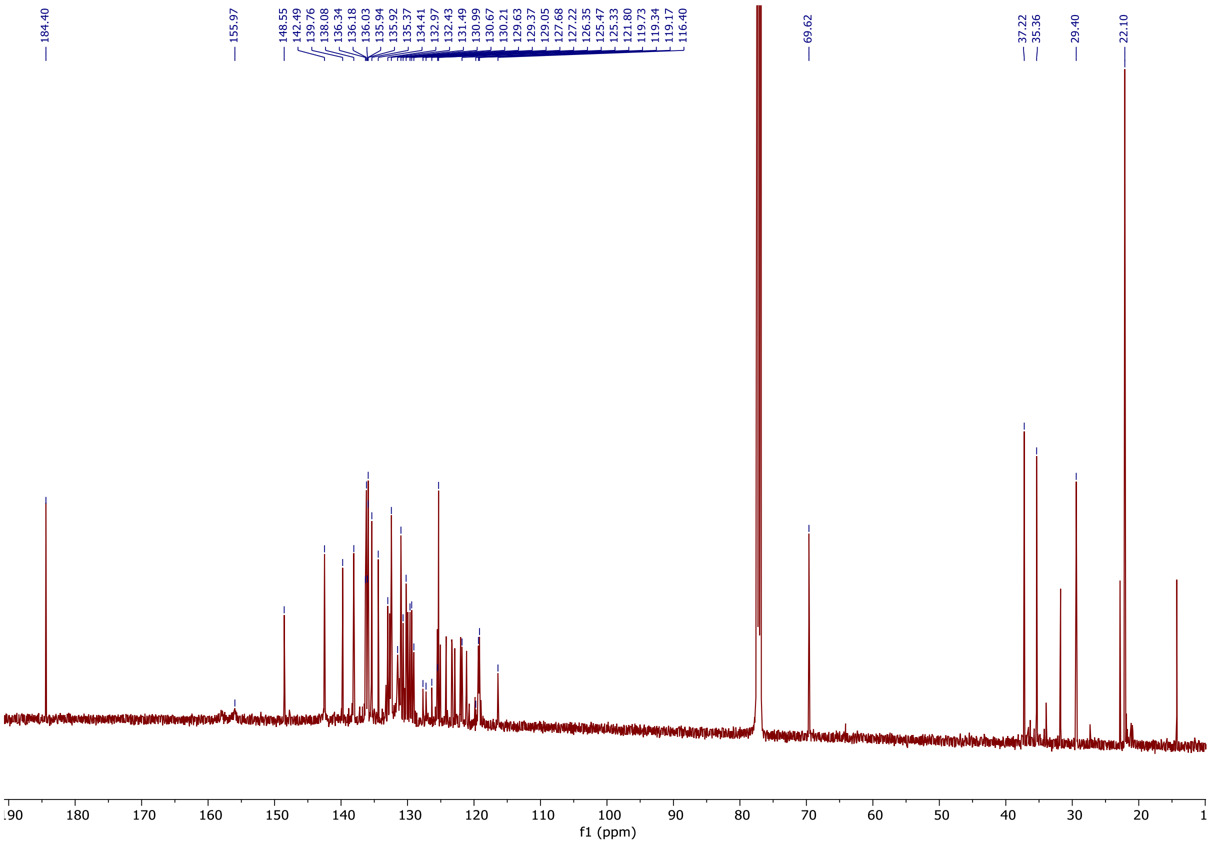

Figure S12. $^{13}C\{^{1}H\}$ NMR spectrum of **4** in $CDCl_3$ at 298 K.

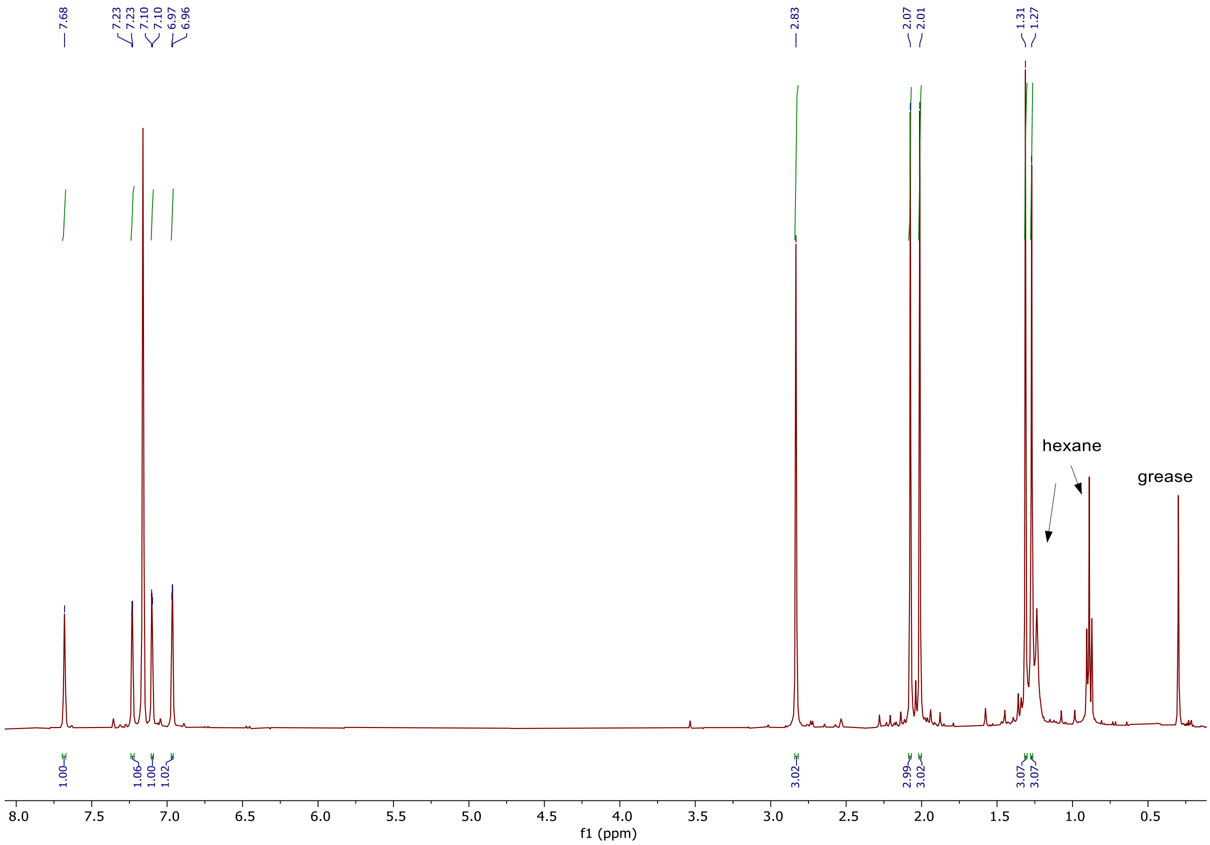

Figure S13. ^{1}H NMR spectrum of **5** in C_6D_6 at 298 K.

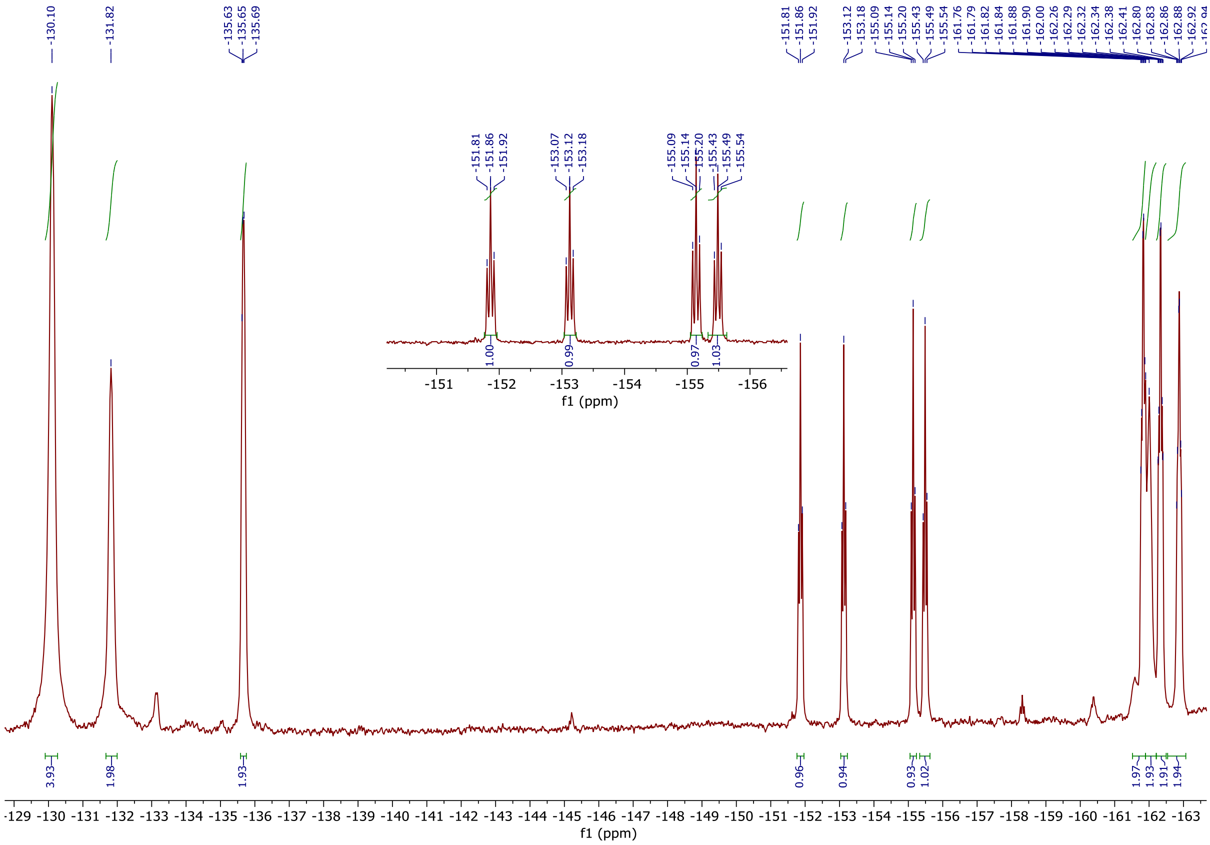

Figure S14. ^{19}F NMR spectrum of **5** in C_6D_6 at 298 K.

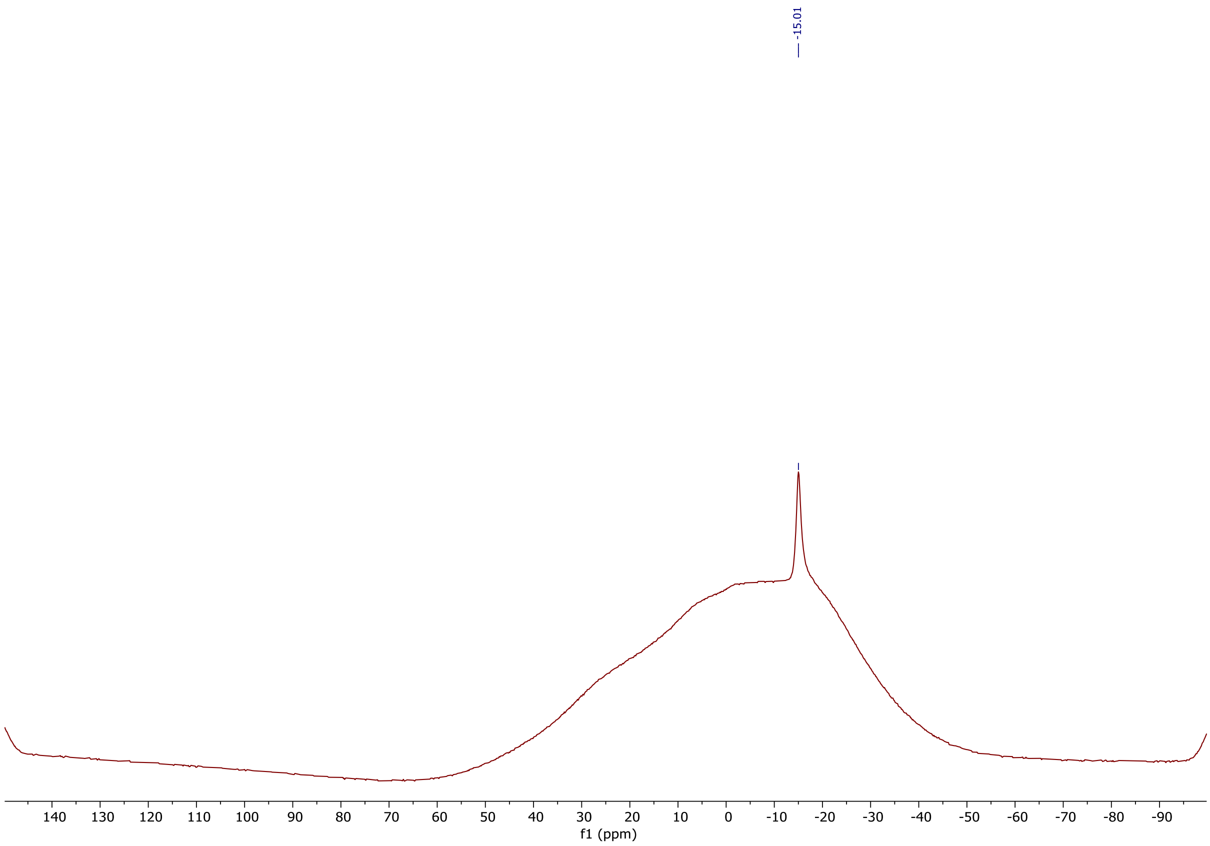

Figure S15. $^{11}B\{^{1}H\}$ NMR spectrum of **5** in C_6D_6 at 298 K.

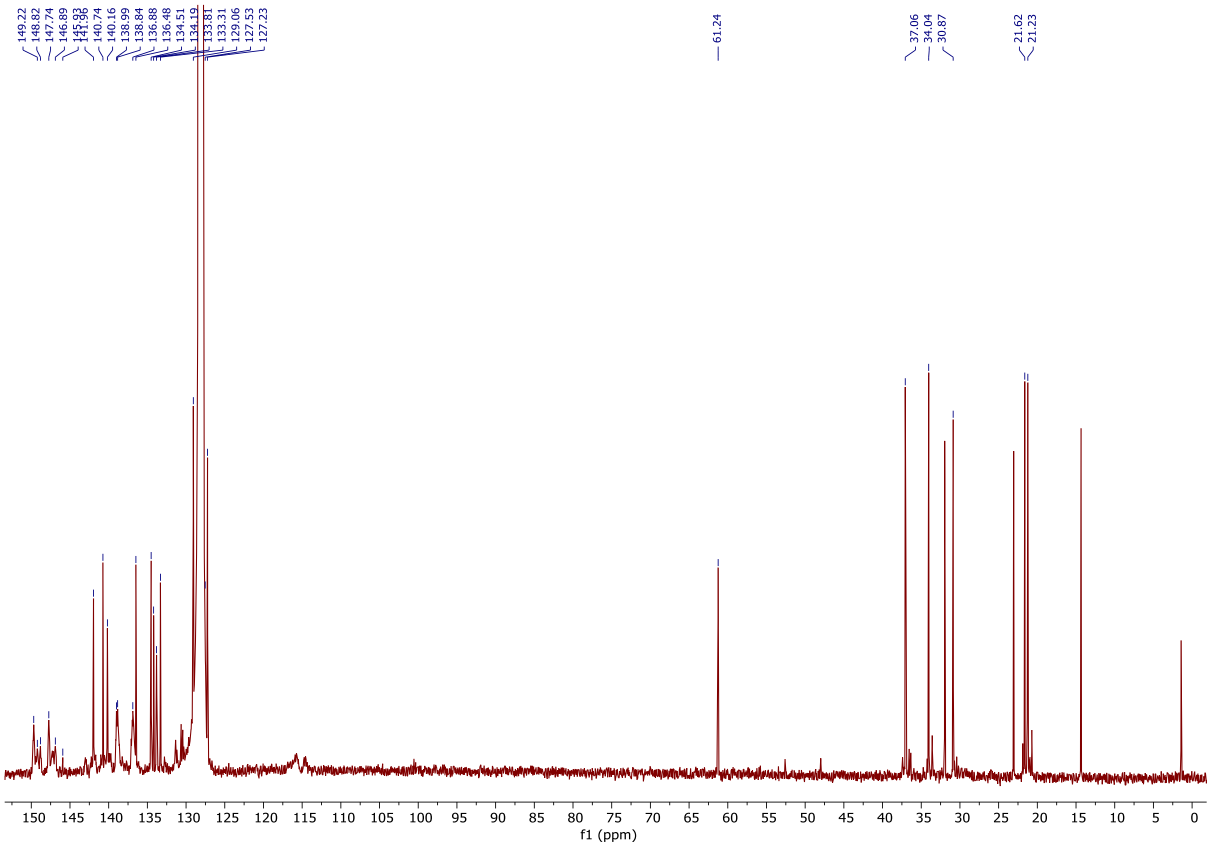

Figure S16. $^{13}C\{^{1}H\}$ NMR spectrum of **5** in C_6D_6 at 298 K.

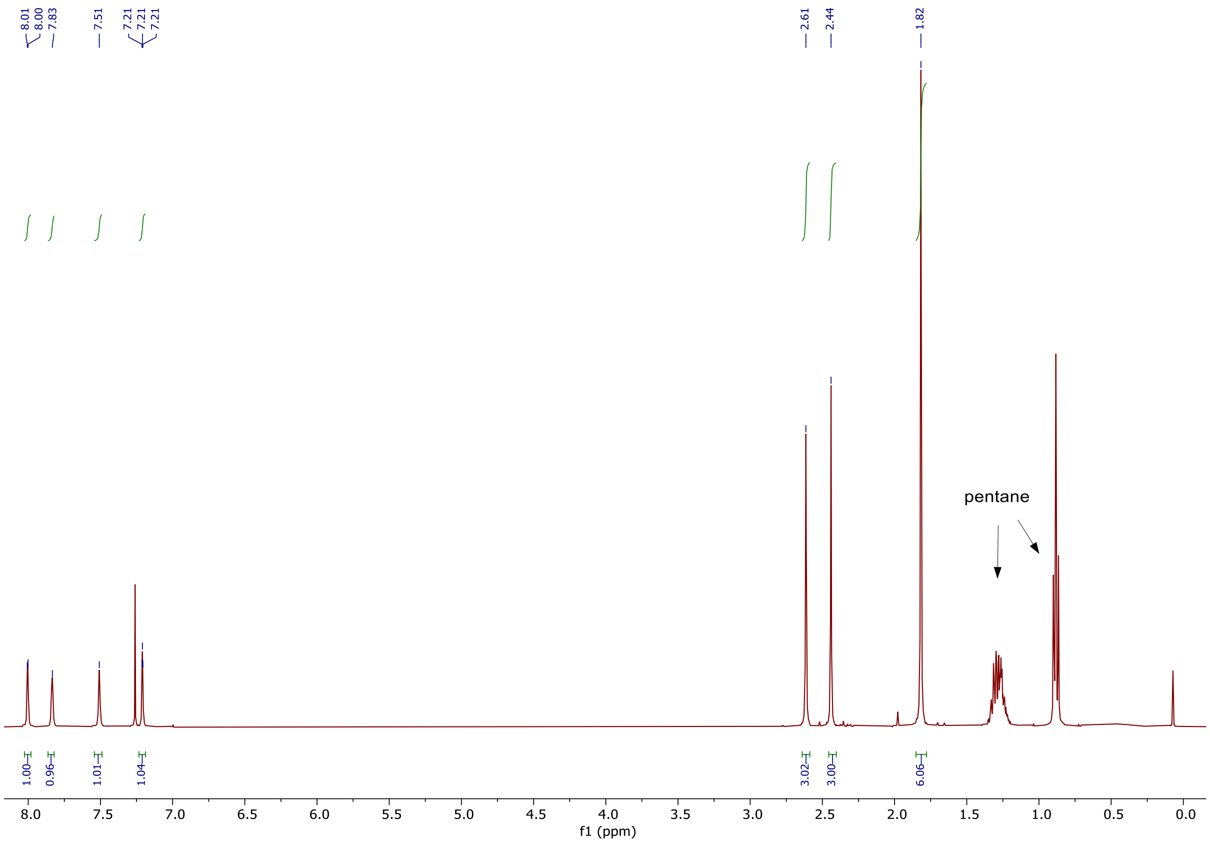

Figure S17. ^{1}H NMR spectrum of **6** in $CDCl_3$ at 298 K.

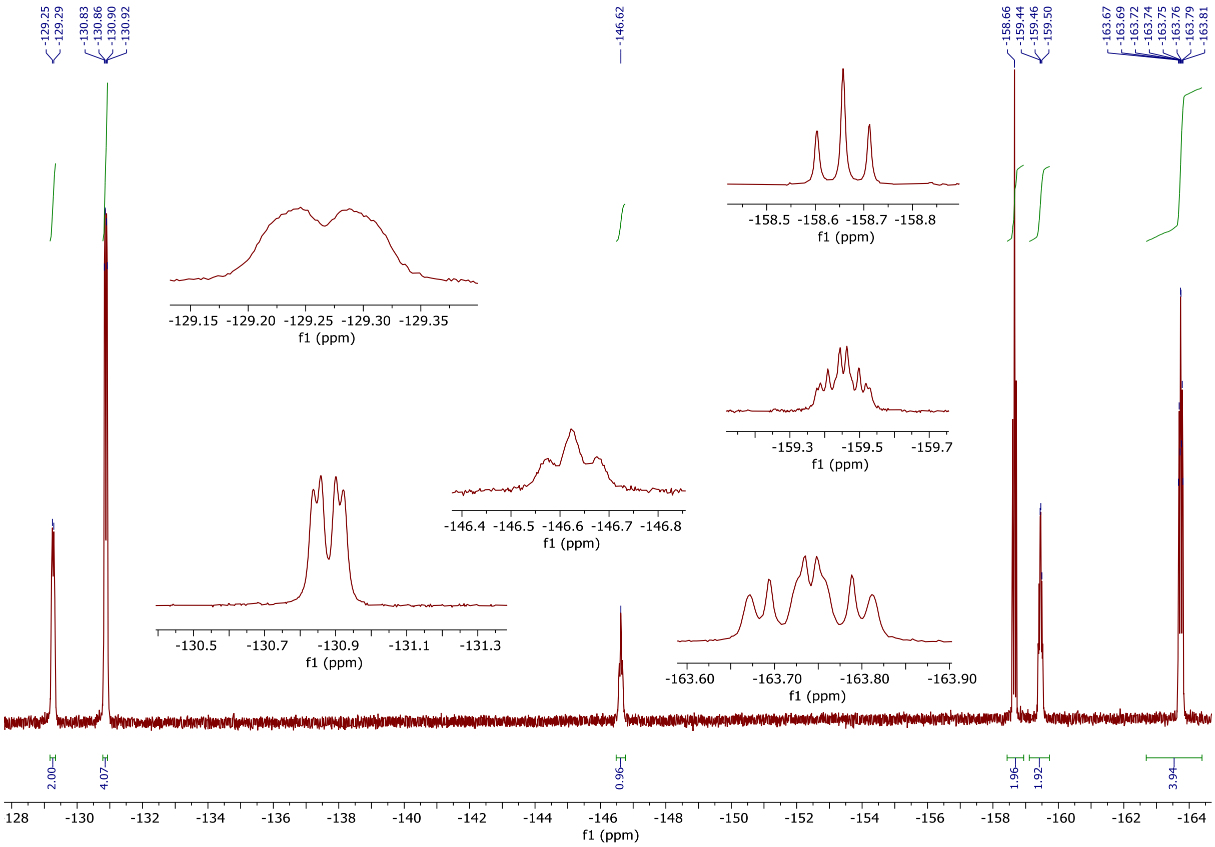

Figure S18. ^{19}F NMR spectrum of **6** in $CDCl_3$ at 298 K.

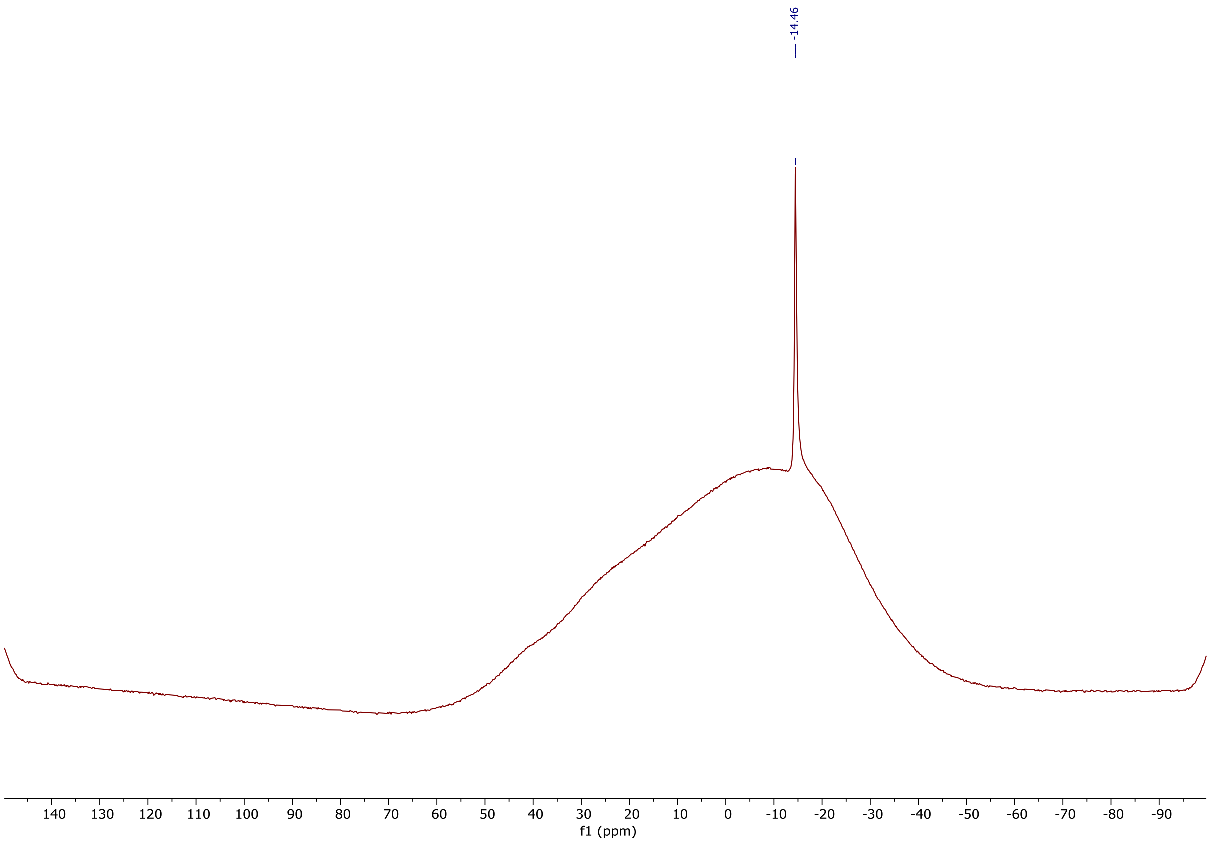

Figure S19. $^{11}B\{^1H\}$ NMR spectrum of **6** in $CDCl_3$ at 298 K.

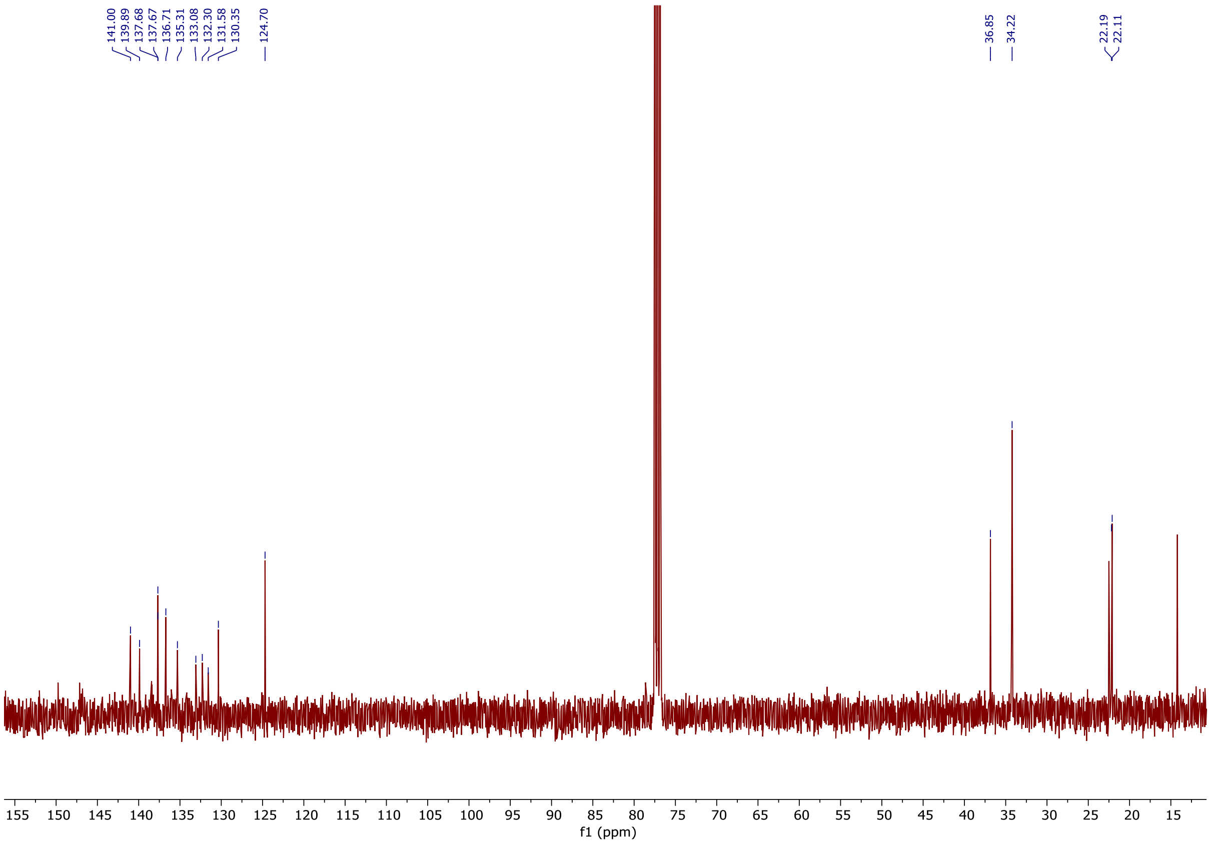

Figure S20. $^{13}C\{^1H\}$ NMR spectrum of **6** in $CDCl_3$ at 298 K.

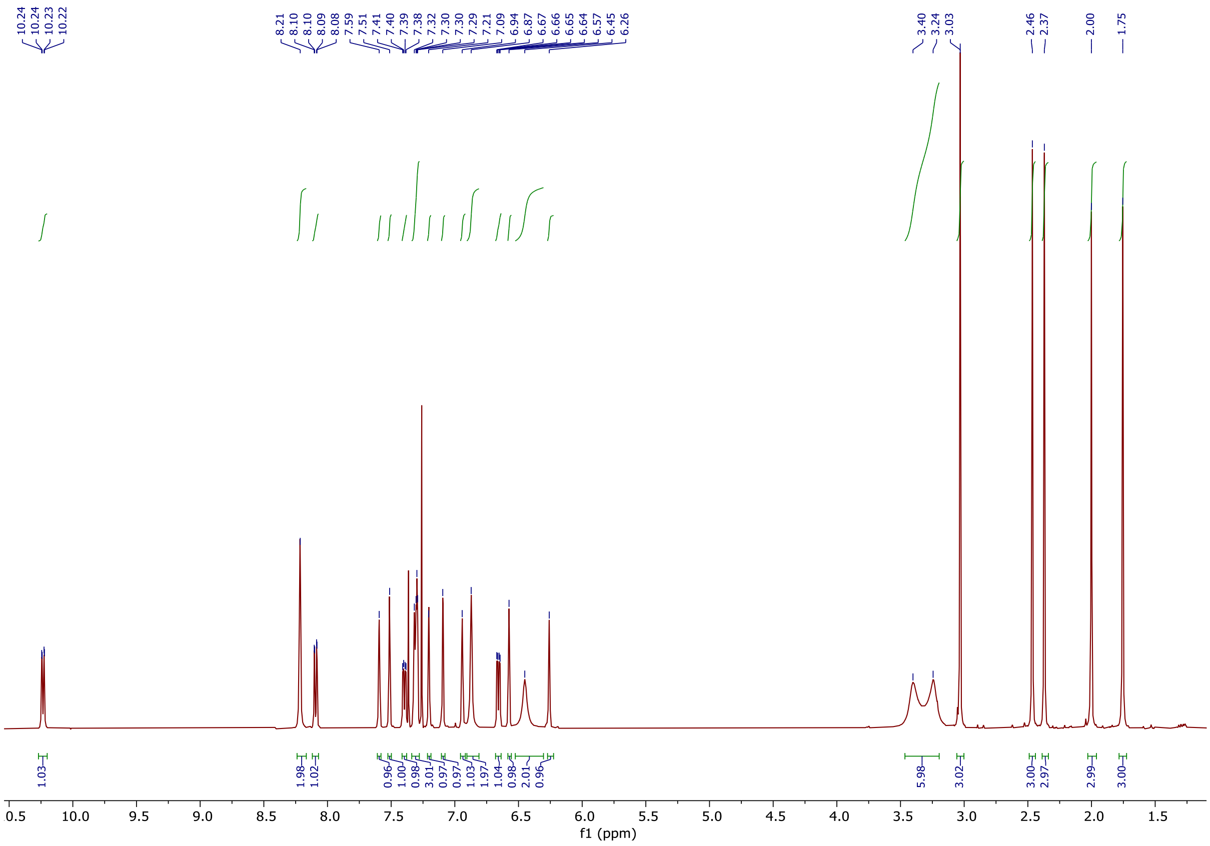

Figure S21. ^{1}H NMR spectrum of **7** in $CDCl_3$ at 298 K.

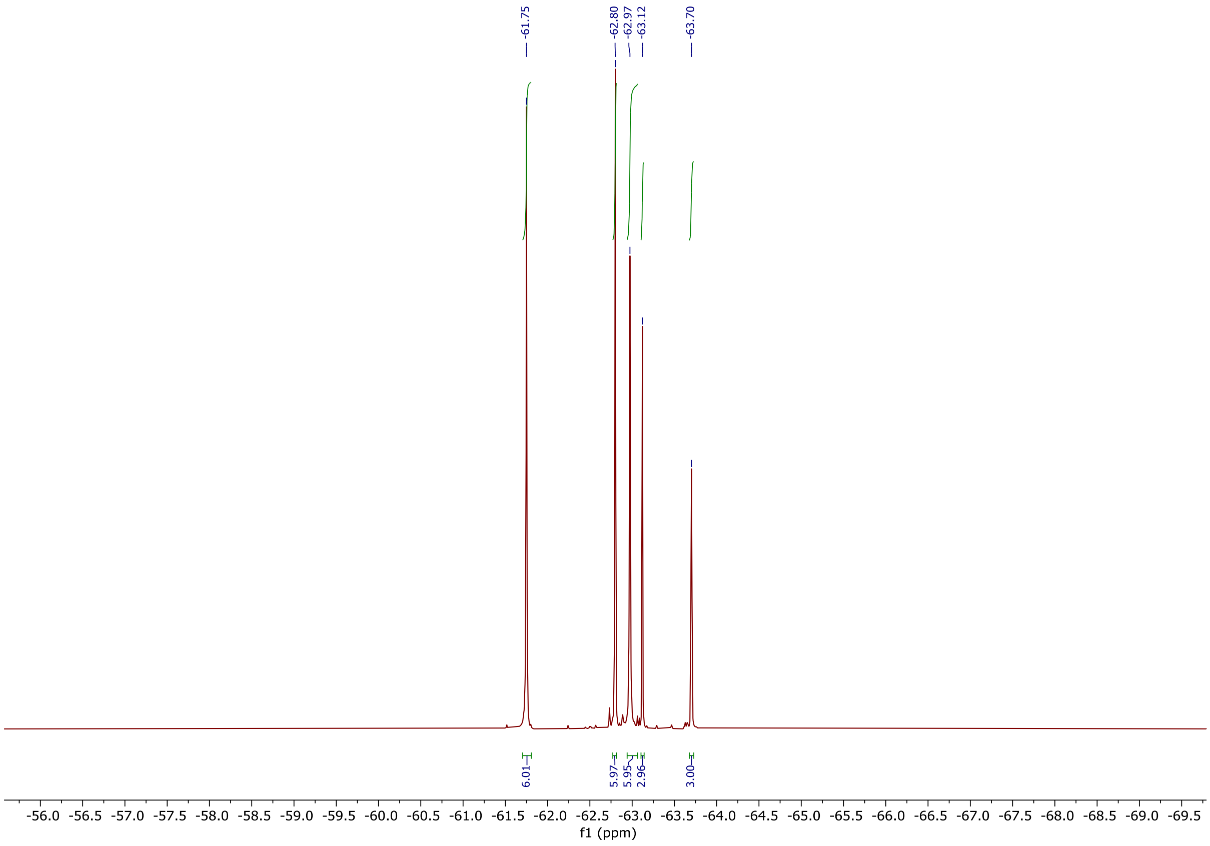

Figure S22. ^{19}F NMR spectrum of **7** in $CDCl_3$ at 298 K.

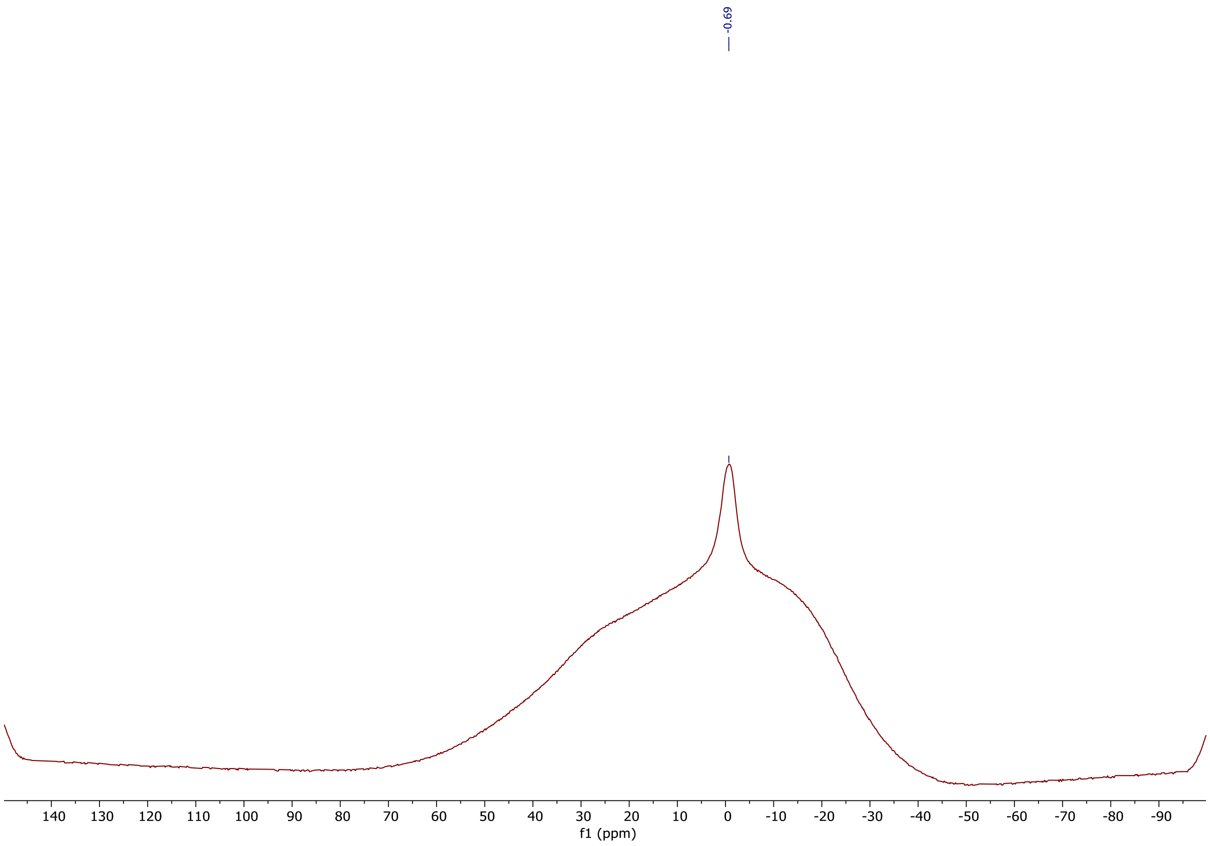

Figure S23. $^{11}B\{^{1}H\}$ NMR spectrum of **7** in $CDCl_3$ at 298 K.

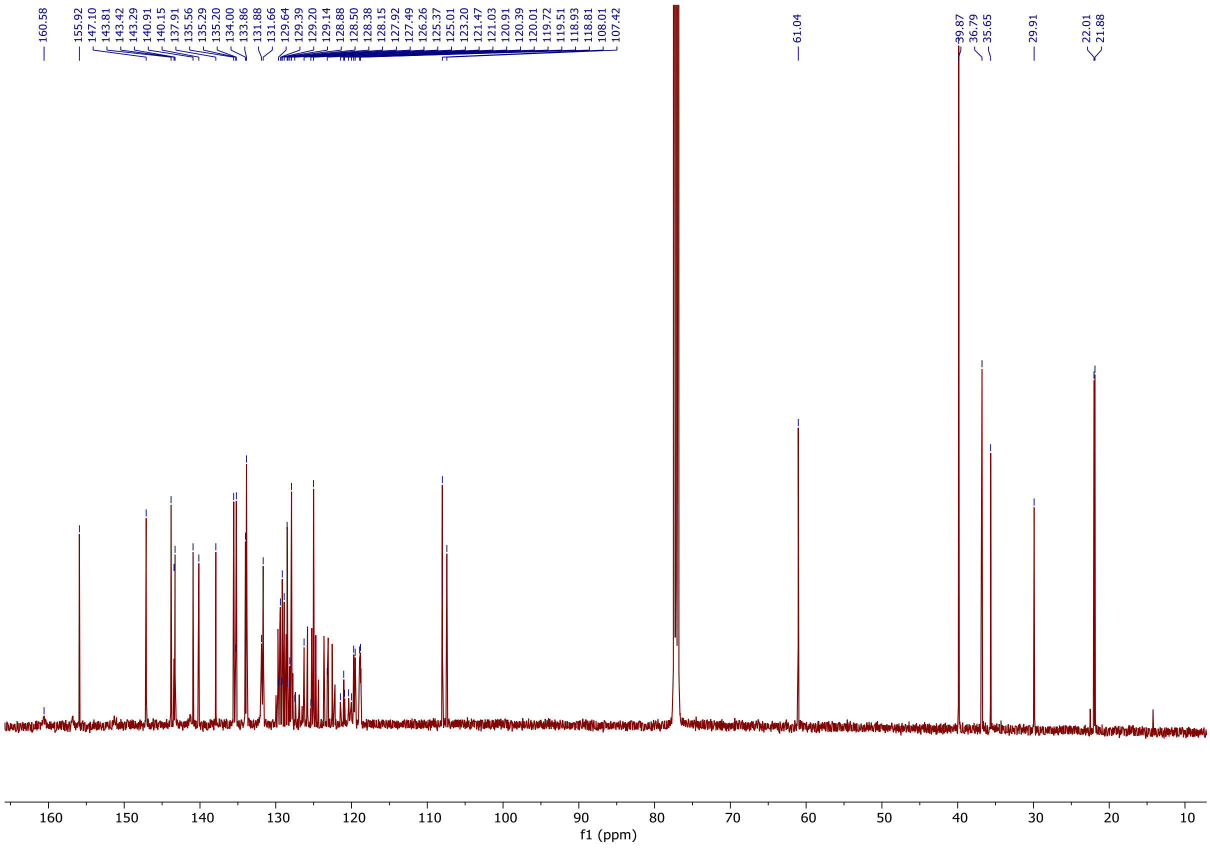

Figure S24. $^{13}C\{^{1}H\}$ NMR spectrum of **7** in $CDCl_3$ at 298 K.

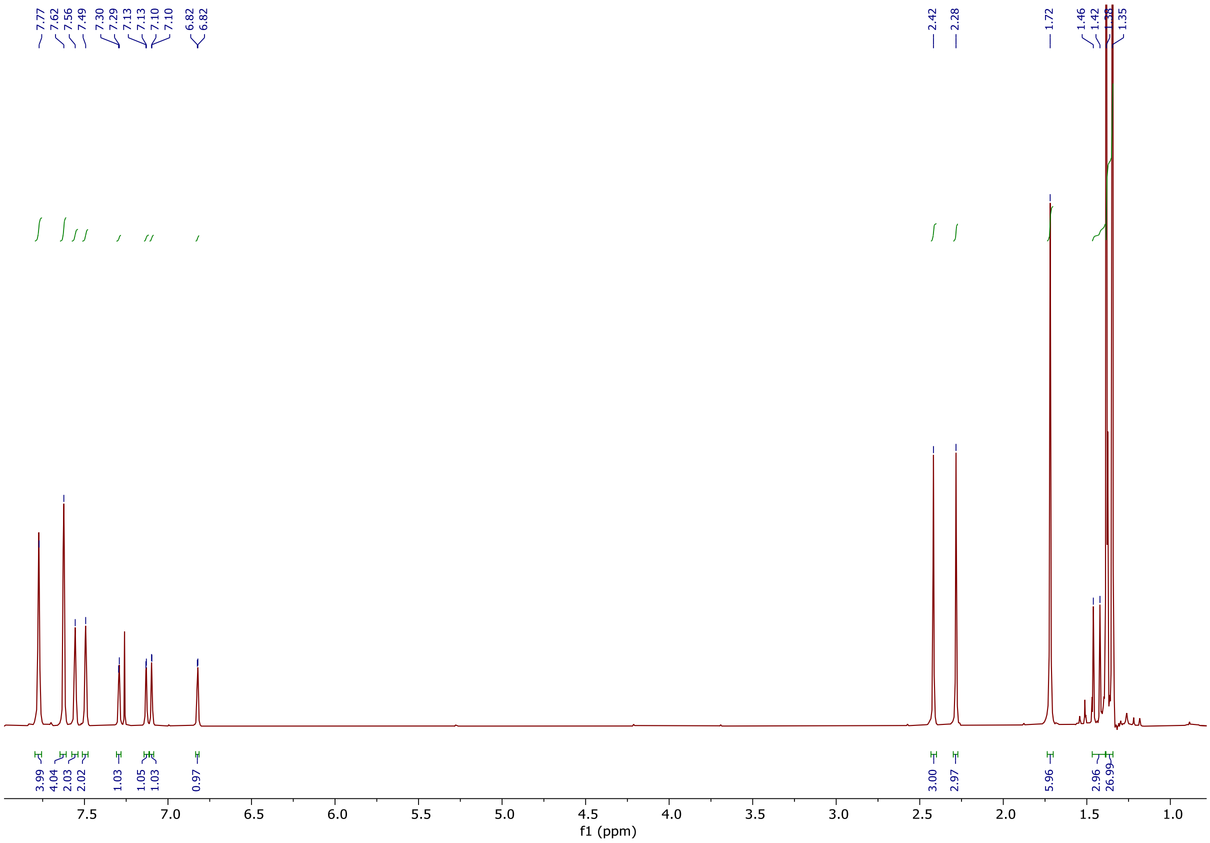

Figure S25. ^{1}H NMR spectrum of **8** in $CDCl_3$ at 298 K.

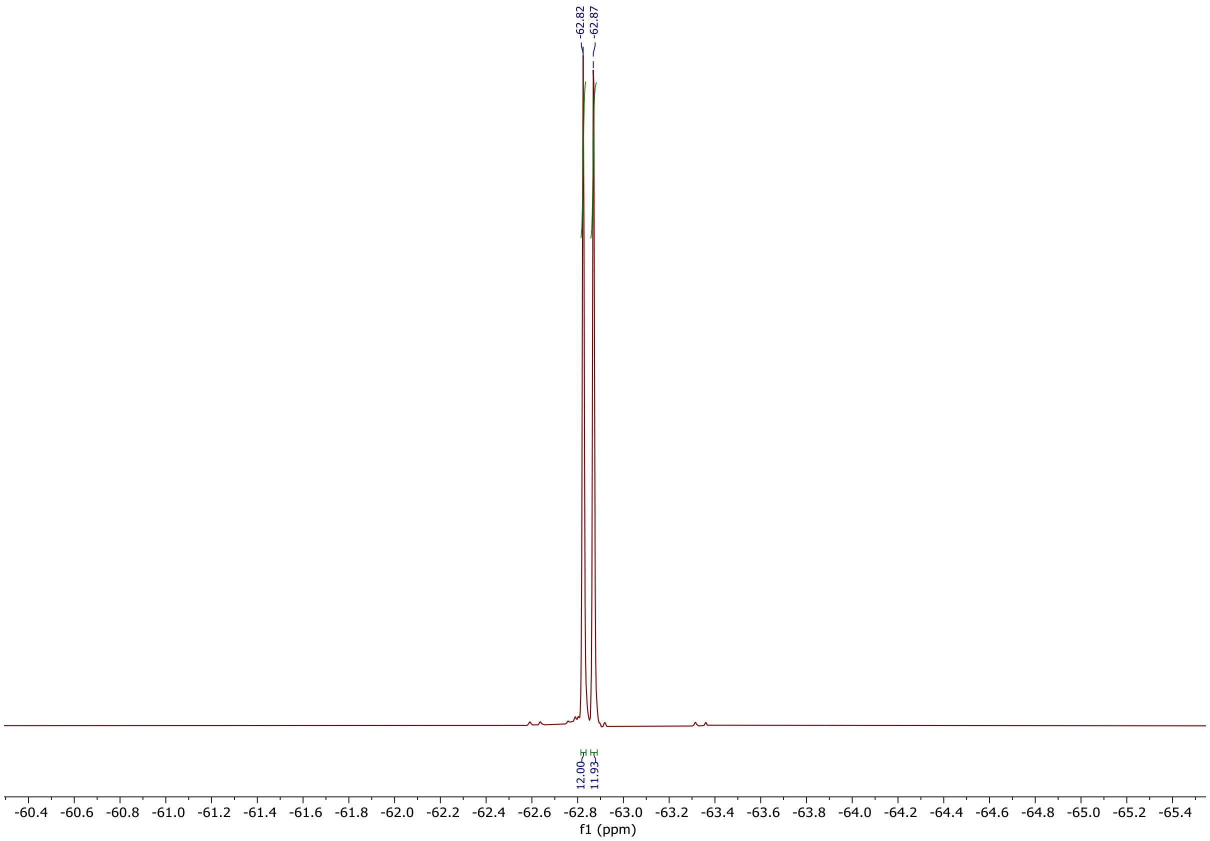

Figure S26. ^{19}F NMR spectrum of **8** in $CDCl_3$ at 298 K.

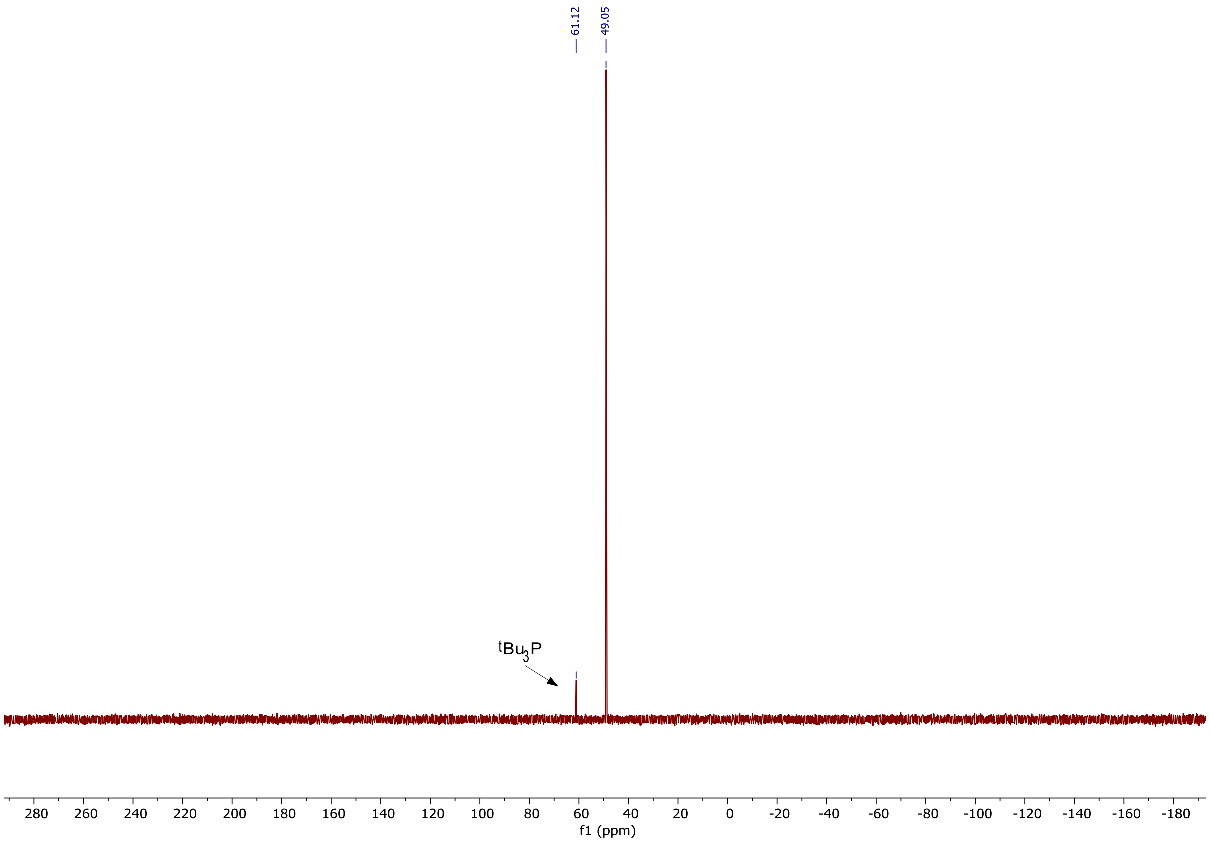

Figure S27. $^{31}P\{^{1}H\}$ NMR spectrum of **8** in $CDCl_3$ at 298 K.

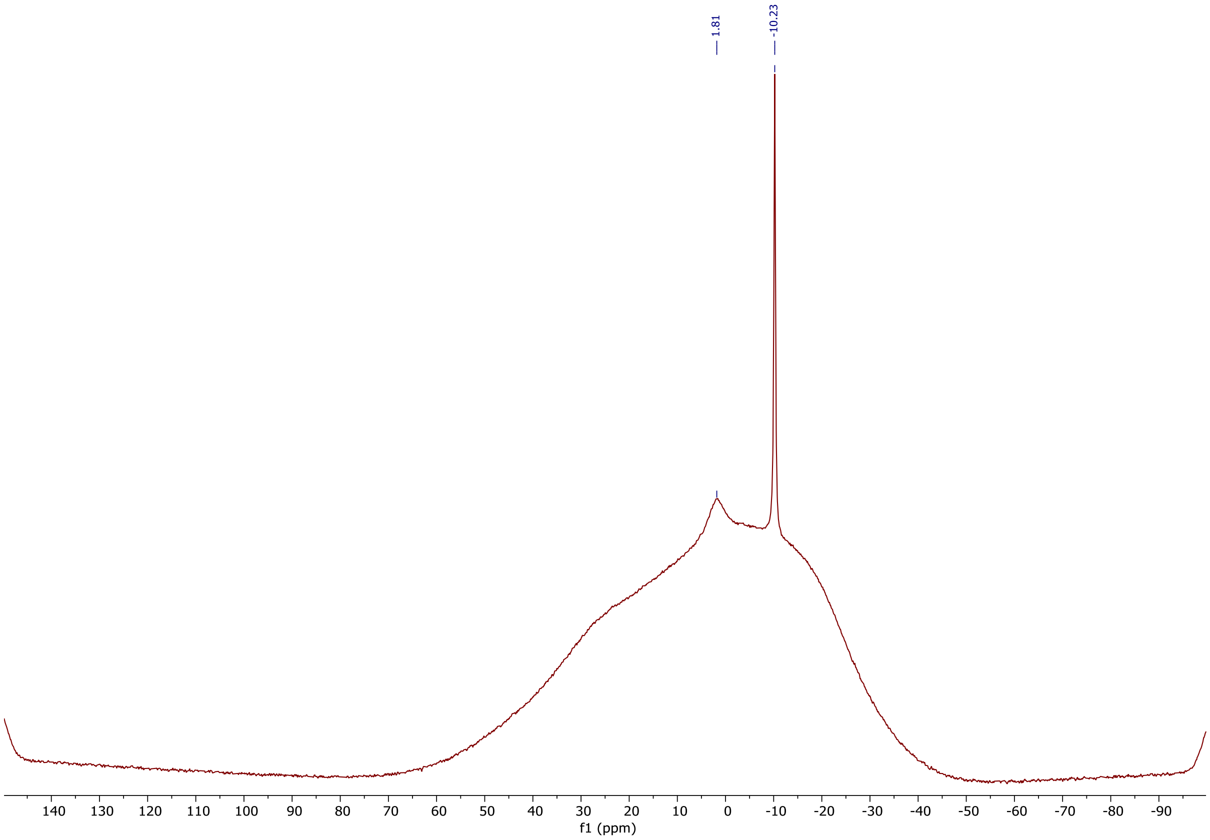

Figure S28. $^{11}B\{^{1}H\}$ NMR spectrum of **8** in $CDCl_3$ at 298 K.

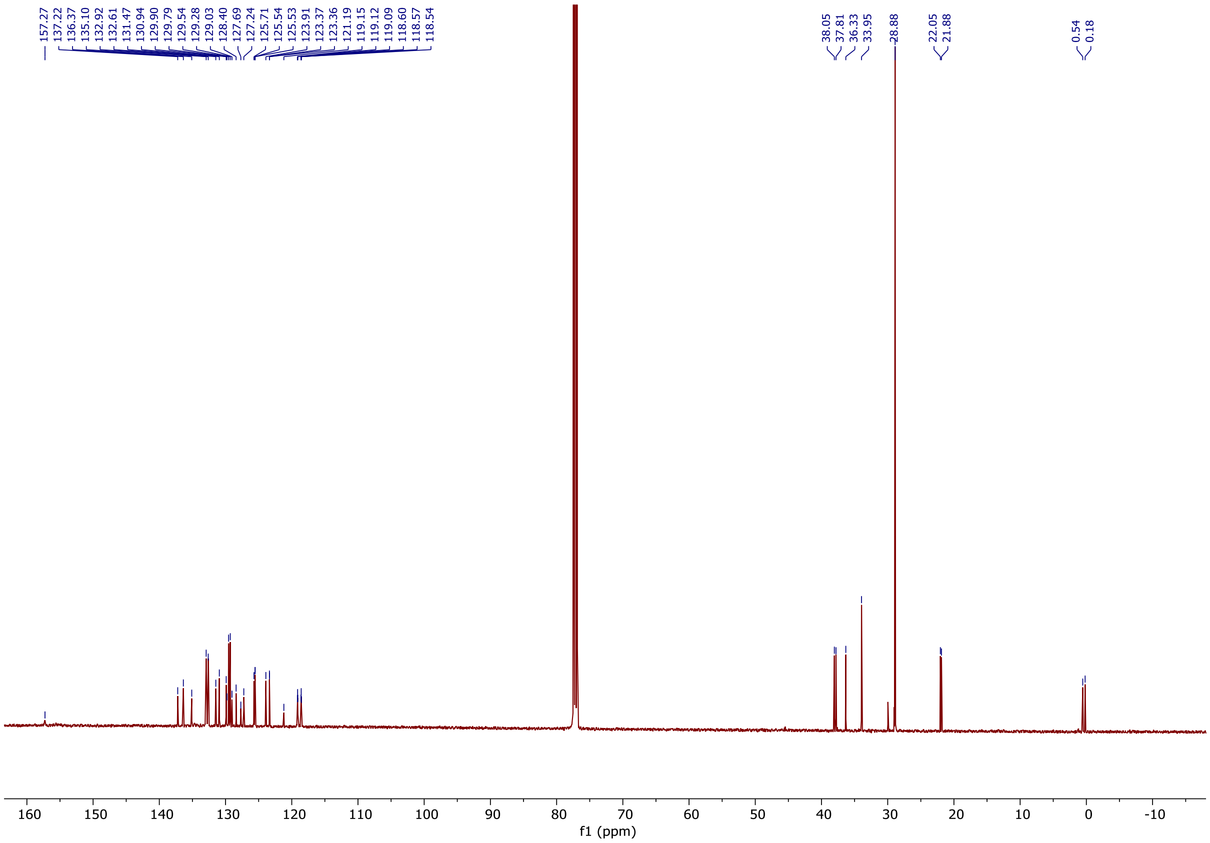

Figure S29. $^{13}C\{^1H\}$ NMR spectrum of **8** in $CDCl_3$ at 298 K.

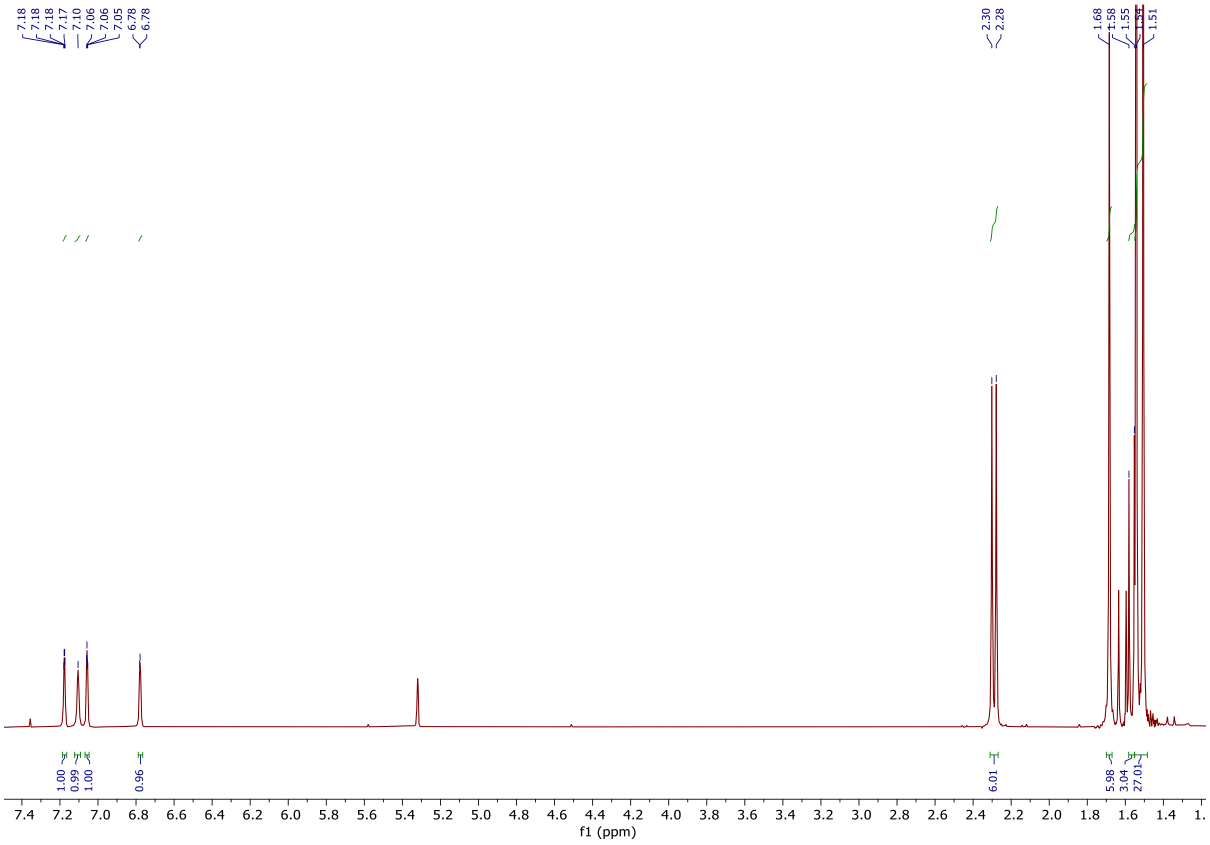

Figure S30. ^{1}H NMR spectrum of **9** in CD_2Cl_2 at 298 K.

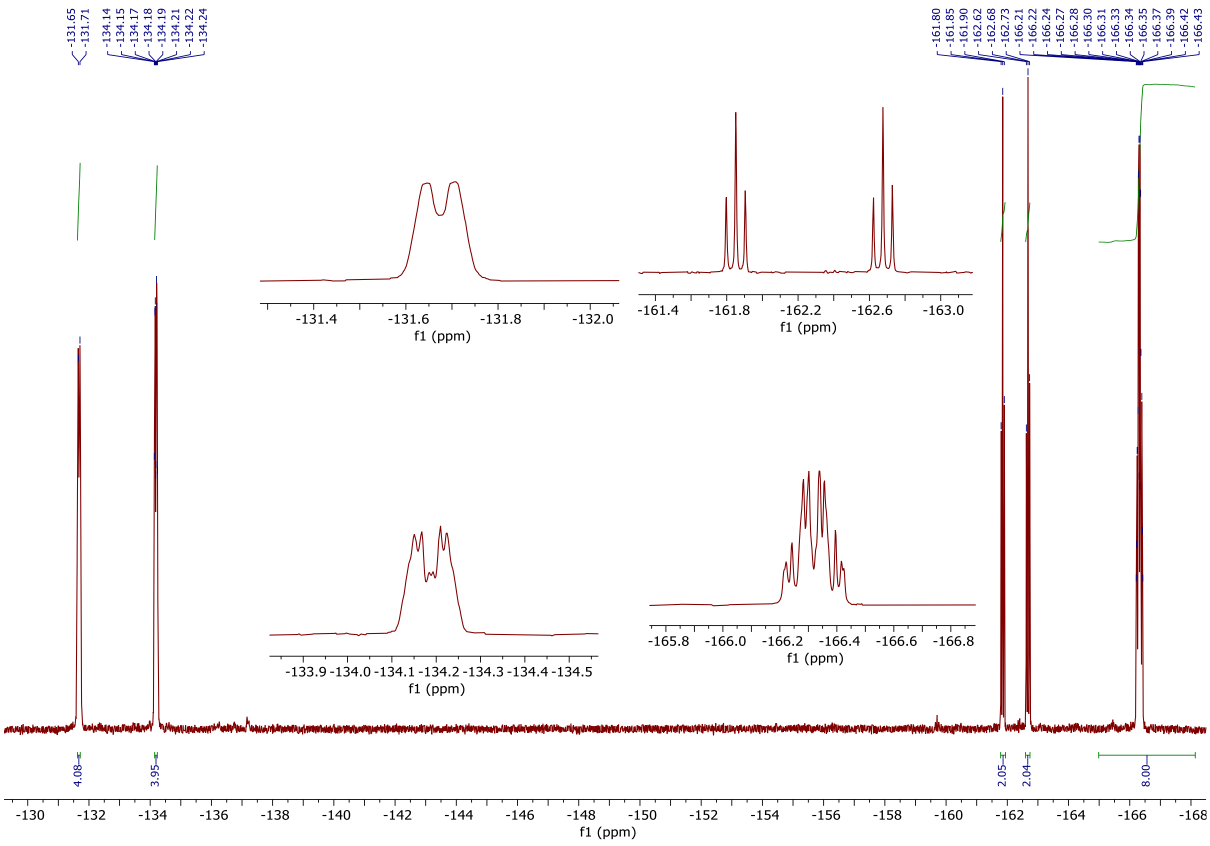

Figure S31. ^{19}F NMR spectrum of **9** in CD_2Cl_2 at 298 K.

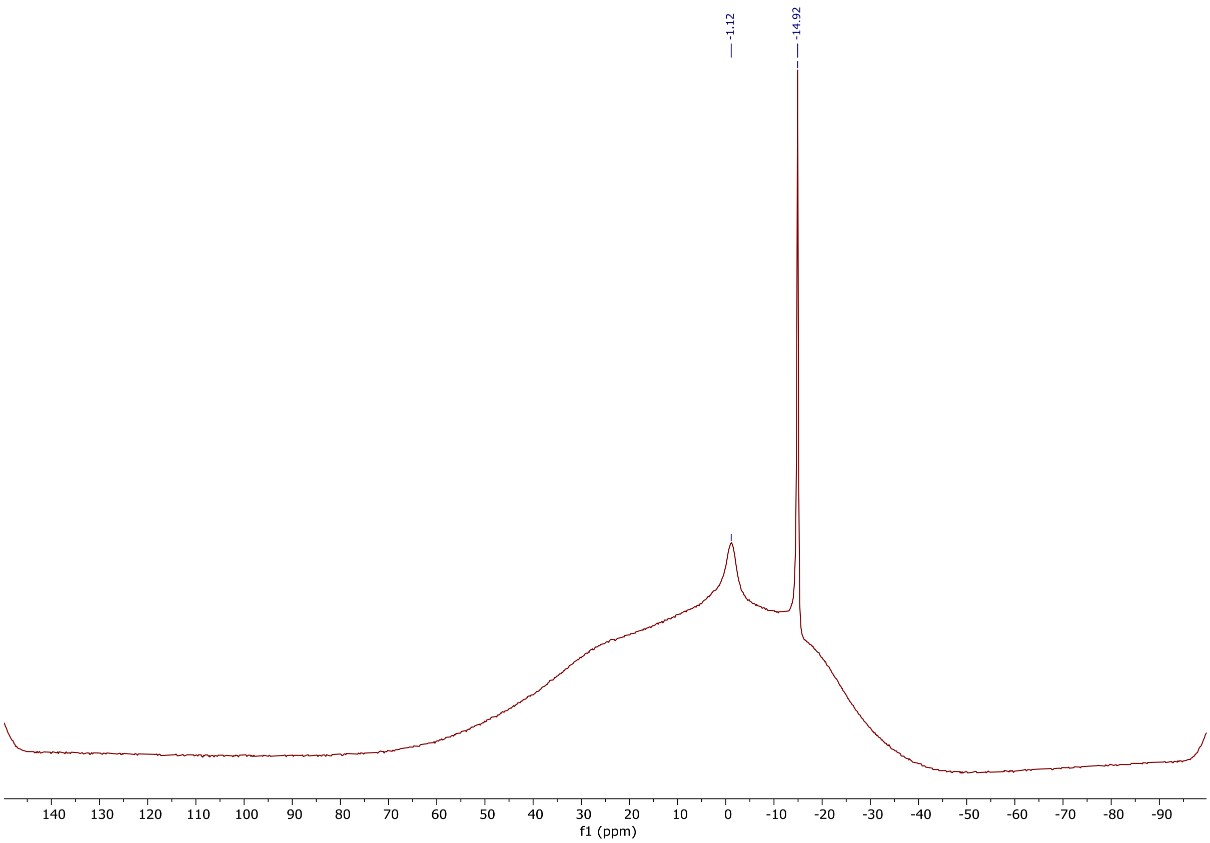

Figure S32. $^{11}B\{^1H\}$ NMR spectrum of **9** in CD_2Cl_2 at 298 K.

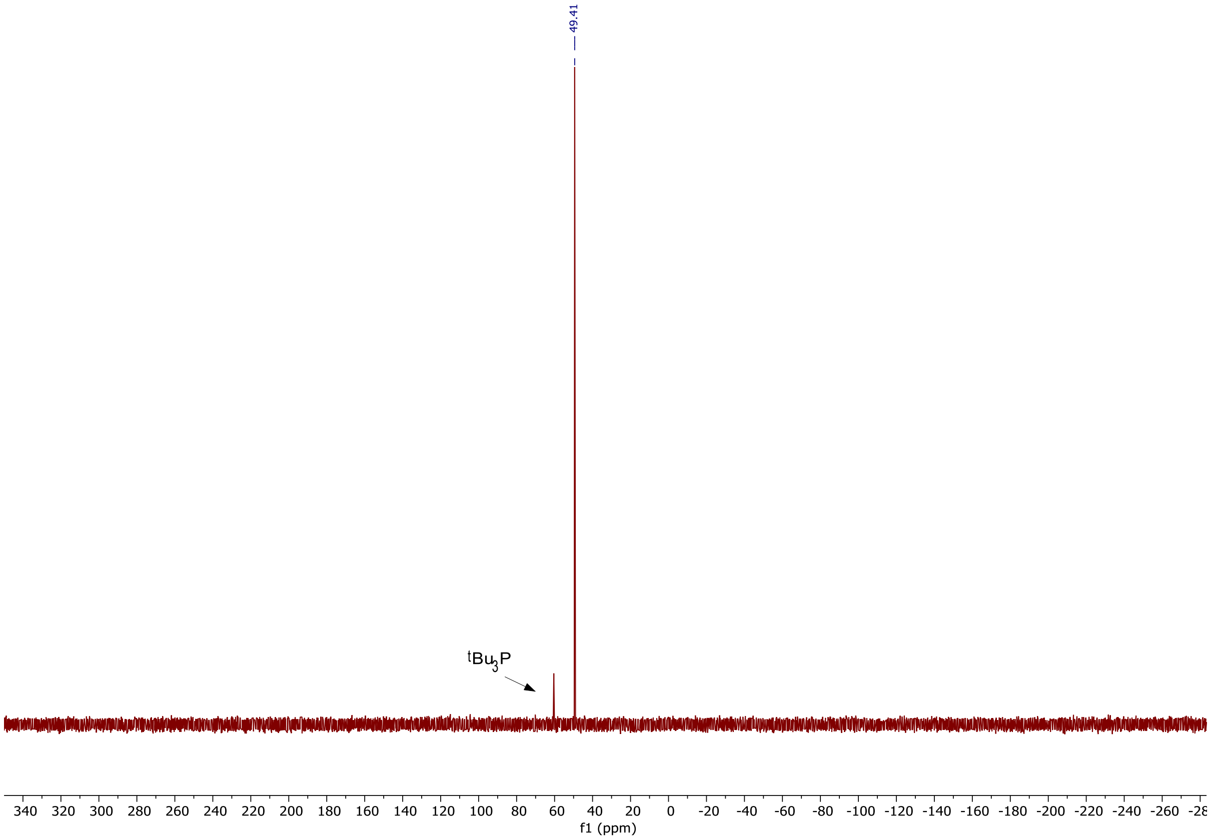

Figure S33. $^{31}P\{^1H\}$ NMR spectrum of **9** in CD_2Cl_2 at 298 K.

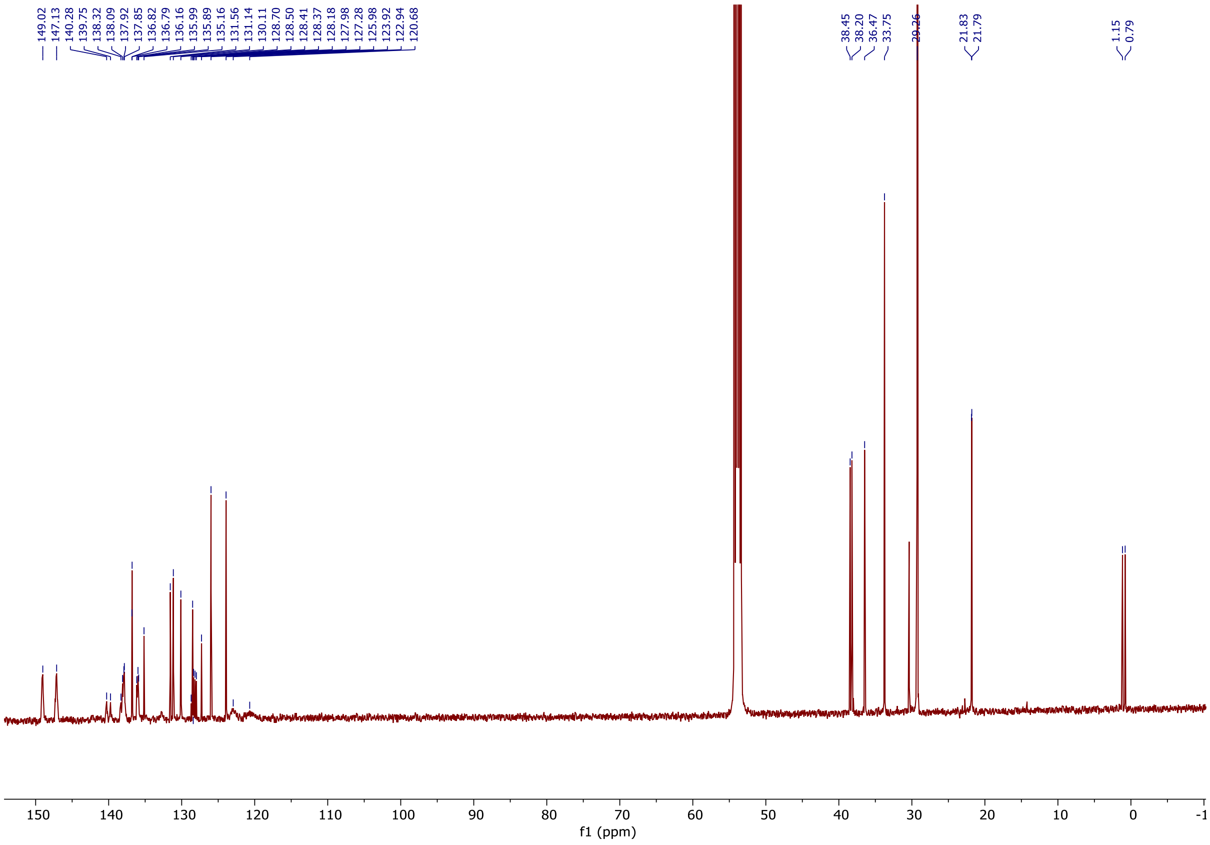

Figure S34. $^{13}C\{^{1}H\}$ NMR spectrum of **9** in CD_2Cl_2 at 298 K.

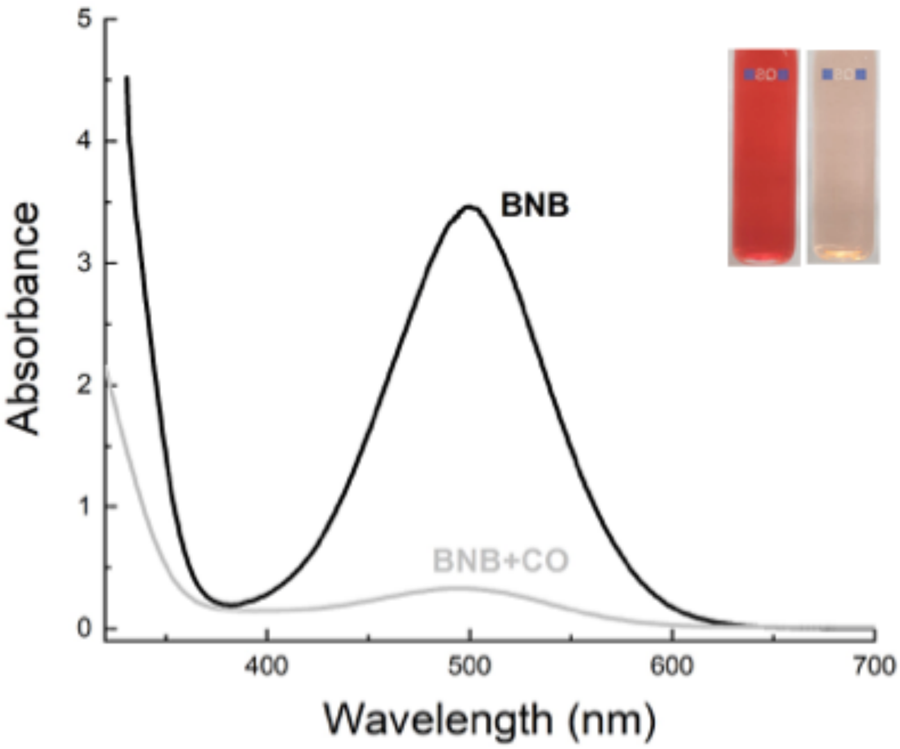

Fig S35. UV/Vis spectra of **3** (black trace) and **5** (grey trace) in chloroform (ca. 0.8 mM concentration); (inset) photographs: solutions of **3** and **5** in chloroform.

4. X-ray crystallographic studies

Table S1. Selected crystallographic and refinement data for X-ray crystal structures.

	**2**·C_6H_6	**4**·0.5(C_7H_8)	**6**·C_5H_{12}	**7**·2($CHCl_3$)
Formula	$C_{56}H_{37}B_2F_{24}N_1$	$C_{109}H_{70}B_4F_{48}N_2O_2$	$C_{41}H_{28}B_2F_{15}N_1O_1$	$C_{60}H_{43}B_2Cl_6F_{24}N_3O_1$
Fw	1201.48	2394.91	857.26	1512.29
Cryst. System	Monoclinic	Monoclinic	Triclinic	Triclinic
Space Group	P 2_1/c	P 2_1/n	P -1	P -1
Wavelength/Å	1.54184	1.54184	1.54184	1.54184
a/Å	16.4401(1)	12.83350(10)	12.2689(5)	12.5944(5)
b/Å	13.1049(1)	23.3049(2)	13.2274(6)	15.0257(7)
c/Å	25.4034(1)	17.50850(10)	13.2354(8)	19.6564(6)
α/°	90	90	105.177(5)	106.987(3)
β/°	103.870(1)	92.4850(10)	113.841(5)	92.159(3)
γ/°	90	90	93.386(4)	112.386(4)
Volume/$Å^3$	5313.48(6)	5231.58(7)	1862.75(17)	3242.4(2)
Z	4	2	2	2
Temp./K	150(2)	150(2)	150(2)	150(2)
Refls. Collect.	11087	10981	7706	13444
$2θ_{max}$	152.39	153.41	152.268	152.262
R_{int}	0.0381	0.0491	0.0402	0.0370
Goodness of fit	1.046	1.062	1.022	1.058
$R[F^2>2σ]$, F	0.0547	0.0613	0.0480	0.0738
R_w (all data), F^2	0.1481	0.1712	0.1379	0.2291
CCDC ref	2082378	2082377	2082380	2082379

Table S1 contd. Selected crystallographic and refinement data for X-ray crystal structures.

	**8**·C_6H_6	**9**·0.33(C_7H_8)	**3-ONNPtBu$_3$**
Formula	$C_{69}H_{64}B_2F_{24}N_1O_1P_1$	$C_{172}H_{146}B_6F_{60}N_3O_3P_3$	$C_{54}H_{46}B_2F_{20}N_3OP$
Fw	1431.80	3600.68	1185.53
Crystal System	Orthorhombic	Monoclinic	Monoclinic
Space Group	P n a 2_1	C 2/c	P 2_1/n
Wavelength/Å	1.54184	1.54184	1.54184
a/Å	23.8866(3)	12.8728(2)	16.4318(2)
b/Å	13.0541(2)	21.0733(4)	18.4549(2)
c/Å	21.7644(3)	59.1848(10)	17.0490(2)
α/°	90	90	90
β/°	90	94.540(2)	93.443(1)
γ/°	90	90	90
Volume/Å^3	6786.53(16)	16004.8(5)	5160.73(10)
Z	4	4	4
Temp/K	150(2)	150(2)	150(2)
Refls collected	11421	16875	10659
R_{int}	0.0385	0.0504	0.0418
Goodness of fit	1.045	1.099	1.043
R [F^2>2σ], F	0.0562	0.0976	0.0433
R_w (all data), F^2	0.1563	0.2401	0.1192
CCDC ref	2082381	2082382	2082376

5. Computational studies

The computational work was performed using DFT within the Gaussian09 (Revision D.01) program package.[5] Geometry optimizations of the monoanionic ligand systems were performed with the PBE1PBE hybrid exchange-correlation functional[6] using a TZVP basis set.[7] Grimme's empirical dispersion correction (DFT-D3) was included in all geometry optimizations.[8] Unless otherwise stated, geometry optimizations were carried out for the full system, and frequency calculations were performed to confirm the nature of the stationary points found (minimum).

xyz coordinates for compound **4**

110

O	-0.195400	0.489400	-0.573900
F	6.409100	4.766500	0.492200
F	6.885500	2.754500	-0.136600
F	-4.452400	2.454100	3.344500
N	0.715200	-1.072800	-2.098800
F	6.290700	4.226900	-1.593600
F	3.629700	-4.676800	0.852000
F	1.576100	-5.319700	0.760600
F	1.090200	0.792900	4.056200
F	-3.200100	2.030900	5.049500
F	2.572000	-5.159200	2.671300
F	-3.602700	4.930100	-3.367800
F	0.666000	-1.092500	5.019400
F	2.707400	-0.462700	4.743200
F	-3.354200	-3.666800	3.411200
F	-2.405300	3.127500	3.366900
C	-0.316200	-2.100800	-1.971600
C	2.042800	-1.544800	-2.442200
C	1.249800	-1.158100	0.434000
C	-2.030600	-0.340900	1.058100
C	3.038000	-0.675300	-1.989500
C	0.911400	-0.252500	-0.768500
C	-2.590800	0.700300	-1.336100
C	-1.424500	-1.728700	-1.209100
C	2.862100	1.747500	-0.528900
C	2.241400	-2.713100	-3.139900
C	-0.149900	-3.308200	-2.638200
C	1.668700	-2.480100	0.401000
H	1.739100	-3.019400	-0.532800
C	1.194000	-0.512400	1.673100
H	0.854600	0.513400	1.723100
C	4.195700	2.151000	-0.647200
H	4.923900	1.494900	-1.106400
C	4.345800	-0.987900	-2.348600
H	5.175700	-0.370300	-2.024400
C	1.991500	-3.149400	1.576500
C	-3.887500	0.288800	-1.661700
H	-4.223900	-0.703400	-1.386200
C	-1.208700	-4.208700	-2.530700
H	-1.152900	-5.167600	-3.033000
C	-2.424100	-2.694800	-1.125700
H	-3.302900	-2.496800	-0.519800
C	4.617200	3.383000	-0.170100
F	-1.232800	-3.493300	3.739500
C	-2.423700	0.824500	1.722800
H	-2.450000	1.764400	1.180000
C	0.279500	-0.101200	-3.162400
H	0.309500	-0.627100	-4.114000
H	0.972200	0.740200	-3.178400
H	-0.726900	0.237700	-2.939400
C	-2.234900	1.989900	-1.725400
H	-1.240500	2.354300	-1.492000
C	-2.344700	-3.920300	-1.785600
C	4.621200	-2.125100	-3.107900
C	-2.027600	-1.513500	1.811500
H	-1.717200	-2.445800	1.351200
C	-4.766100	1.111400	-2.348500
C	1.539200	-1.176600	2.834100
C	1.957100	2.641200	0.068100
H	0.914500	2.357700	0.153500
C	1.936500	-2.507400	2.798400
H	2.193800	-3.033000	3.709700
C	3.573000	-2.967900	-3.477600
H	3.815400	-3.861200	-4.043200
C	-4.388400	2.392900	-2.733100
H	-5.071900	3.034700	-3.274300
C	1.091200	-3.657000	-3.468100
C	-2.776100	0.820100	3.065400
C	3.711900	4.250900	0.419100
H	4.041800	5.212900	0.794000
C	-3.114400	2.822100	-2.413500
C	2.379200	3.873400	0.533200
C	-2.776700	-0.361000	3.793600
H	-3.054500	-0.367700	4.839300
C	-2.399000	-1.528100	3.151800
C	6.056200	3.788100	-0.346000
C	2.440500	-4.581300	1.476600
C	0.751800	-3.553700	-4.966300
H	0.430400	-2.548000	-5.243300
H	-0.057800	-4.242700	-5.215500
H	1.622400	-3.811700	-5.574500
C	6.033300	-2.474400	-3.474300
H	6.658100	-1.582700	-3.543900
H	6.079400	-2.997800	-4.430800
H	6.477800	-3.129800	-2.719100
C	-3.471100	-4.905500	-1.686200
H	-4.368100	-4.522700	-2.181100
H	-3.734100	-5.096300	-0.642800
H	-3.213000	-5.858700	-2.150200
C	1.493700	-0.475300	4.166400
F	-6.252800	-0.695500	-2.673300
F	-2.617700	-2.699400	5.194600
C	1.530300	-5.097700	-3.162700
H	2.408600	-5.371100	-3.749000
H	0.745100	-5.806100	-3.425700
H	1.765500	-5.229600	-2.104800
B	-1.554000	-0.253100	-0.503000
B	2.361000	0.391900	-1.070000
C	-3.206100	2.107500	3.711500
C	-6.161400	0.643100	-2.652100
C	1.410700	4.844600	1.159400
F	0.203100	4.307500	1.333600
F	-6.588300	1.087600	-3.845200
F	-1.655800	4.084200	-3.764100

C	-2.631300	4.179500	-2.836100
C	-2.401600	-2.839600	3.882400
F	-7.045200	1.079200	-1.738900
F	-2.109600	4.867000	-1.808800
F	1.851100	5.260100	2.355800
F	1.264400	5.938000	0.395900

xyzordinates for compound **Int-CF$_3$**

110

C	-2.746700	1.029400	4.363900
C	-2.801900	0.882800	2.984300
C	-1.732000	0.377100	2.250100
C	-0.578500	0.071000	2.962100
C	-0.432100	0.251800	4.339500
C	-1.556600	0.718000	5.012700
N	0.536800	-0.559000	2.223300
C	1.863400	-0.249100	2.792700
C	2.036100	-0.080600	4.152100
C	0.858300	-0.032300	5.111600
C	2.852600	-0.134100	1.827500
C	4.143000	0.087300	2.286900
C	4.415000	0.167700	3.653000
C	3.357600	0.103500	4.558100
C	0.735900	-1.362600	5.878300
C	1.097800	1.086900	6.137800
C	5.822400	0.342500	4.139700
C	-3.938200	1.507800	5.137100
C	0.336100	-2.058100	2.170500
B	-1.863600	0.191900	0.662400
B	2.254400	-0.163100	0.331200
C	-2.558400	-1.183700	0.183900
C	-2.451900	1.495500	-0.075200
C	2.595400	1.177600	-0.521800
C	2.519400	-1.556600	-0.471400
C	2.684400	2.412400	0.129300
C	2.964900	3.581700	-0.561300
C	3.168800	3.558800	-1.934000
C	3.089400	2.345000	-2.593000
C	2.810000	1.172900	-1.897200
C	3.579200	-2.388900	-0.115300
C	3.854900	-3.555800	-0.818500
C	3.068700	-3.936800	-1.892700
C	2.002700	-3.126300	-2.253700
C	1.730500	-1.958800	-1.552500
C	-3.804700	1.572200	-0.402500
C	-4.350500	2.717300	-0.971400
C	-3.553900	3.814300	-1.251300
C	-2.202800	3.749500	-0.942600
C	-1.663900	2.613300	-0.356200
C	-3.151900	-2.065800	1.080900
C	-3.784600	-3.228700	0.647600
C	-3.847700	-3.533400	-0.698500
C	-3.268500	-2.657600	-1.610800
C	-2.637000	-1.504100	-1.177900
C	0.690400	-0.146600	0.753900
O	-0.324500	0.104900	0.130800
C	3.117700	4.873700	0.192700
F	4.382700	5.040000	0.625400
F	2.822700	5.937700	-0.561600
F	2.332600	4.917200	1.279200
C	3.318800	2.261300	-4.077200
F	4.409800	1.530100	-4.363700
F	2.287100	1.673900	-4.702800
F	3.491600	3.465200	-4.634400
C	5.009800	-4.407800	-0.369800
F	4.791500	-4.919400	0.856000
F	5.236200	-5.436500	-1.192600
F	6.146300	-3.698200	-0.290900
C	1.092600	-3.536100	-3.379900
F	-0.019800	-4.131300	-2.911900
F	0.696500	-2.482800	-4.104300
F	1.680400	-4.402700	-4.212400
C	-5.826400	2.747900	-1.255900
F	-6.543300	2.769800	-0.116400
F	-6.186800	3.821300	-1.967700
F	-6.222100	1.662100	-1.936500
C	-1.297700	4.901500	-1.283200
F	-1.972800	6.049900	-1.418600
F	-0.364400	5.093600	-0.339400
F	-0.643900	4.690200	-2.438100
C	-4.386600	-4.142100	1.677900
F	-5.310500	-3.509700	2.419100
F	-4.972200	-5.212900	1.135600
F	-3.450700	-4.591500	2.537100
C	-3.403900	-2.955500	-3.080900
F	-3.216100	-4.256800	-3.340100
F	-4.634400	-2.644900	-3.523900
F	-2.531900	-2.263400	-3.818800
H	-3.708500	1.168000	2.459400
H	-1.509100	0.859500	6.085500
H	4.952600	0.222000	1.575300
H	3.580100	0.229000	5.611100
H	0.582300	-2.215900	5.215700
H	-0.106200	-1.322500	6.572700
H	1.646900	-1.548100	6.451900
H	1.176200	2.060300	5.650700
H	2.014900	0.905500	6.697900
H	0.292300	1.128900	6.870400
H	6.396100	0.983600	3.467800
H	6.336100	-0.622700	4.186400
H	5.851000	0.780700	5.138600
H	-4.561300	2.170000	4.533800
H	-3.640900	2.044300	6.039900
H	-4.561900	0.663100	5.445900
H	1.180200	-2.498700	1.642800
H	0.295300	-2.412600	3.196200
H	-0.602200	-2.265100	1.658700
H	2.540900	2.465400	1.203700
H	3.379200	4.470700	-2.477600
H	2.767600	0.240000	-2.448700
H	4.209900	-2.122600	0.726500
H	3.283800	-4.843900	-2.442300
H	0.889600	-1.348400	-1.867900
H	-4.451600	0.719600	-0.220800
H	-3.975600	4.703400	-1.701700
H	-0.604500	2.606000	-0.121800
H	-3.133000	-1.843500	2.143700
H	-4.339100	-4.436700	-1.038100
H	-2.207800	-0.832400	-1.913700

6. References

[s1] E. W. Y. Wong, D. J. H. Emslie, *Dalton Transactions* **2015**, *44*, 11601-11612.

[s2] P. J. Bailey, R. A. Coxall, C. M. Dick, S. Fabre, L. C. Henderson, C. Herber, S. T. Liddle, D. Loroño-González, A. Parkin, S. Parsons, *Chemistry – A European Journal* **2003**, *9*, 4820-4828.

[s3] K. Samigullin, M. Bolte, H. W. Lerner, M. Wagner, *Organometallics* **2014**, *33*, 3564-3569.

[s4] D. J. Parks, W. E. Piers, G. P. A. Yap, *Organometallics* **1998**, *17*, 5492-5503.

[s5] M. J. Frisch, G. W. Trucks, H. B. Schlegel, G. E. Scuseria, M. A. Robb, J. R. Cheeseman, G. Scalmani, V. Barone, G. A. Petersson, H. Nakatsuji, X. Li, M. Caricato, A. V. Marenich, J. Bloino, B. G. Janesko, R. Gomperts, B. Mennucci, H. P. Hratchian, J. V. Ortiz, A. F. Izmaylov, J. L. Sonnenberg, Williams, F. Ding, F. Lipparini, F. Egidi, J. Goings, B. Peng, A. Petrone, T. Henderson, D. Ranasinghe, V. G. Zakrzewski, J. Gao, N. Rega, G. Zheng, W. Liang, M. Hada, M. Ehara, K. Toyota, R. Fukuda, J. Hasegawa, M. Ishida, T. Nakajima, Y. Honda, O. Kitao, H. Nakai, T. Vreven, K. Throssell, J. A. Montgomery Jr., J. E. Peralta, F. Ogliaro, M. J. Bearpark, J. J. Heyd, E. N. Brothers, K. N. Kudin, V. N. Staroverov, T. A. Keith, R. Kobayashi, J. Normand, K. Raghavachari, A. P. Rendell, J. C. Burant, S. S. Iyengar, J. Tomasi, M. Cossi, J. M. Millam, M. Klene, C. Adamo, R. Cammi, J. W. Ochterski, R. L. Martin, K. Morokuma, O. Farkas, J. B. Foresman, D. J. Fox, Wallingford, CT, **2016**.

[s6] aC. Adamo, V. Barone, *The Journal of Chemical Physics* **1999**, *110*, 6158-6170; bM. Ernzerhof, G. E. Scuseria, *The Journal of Chemical Physics* **1999**, *110*, 5029-5036; cJ. P. Perdew, K. Burke, M. Ernzerhof, *Physical Review Letters* **1996**, *77*, 3865-3868; dJ. P. Perdew, M. Ernzerhof, K. Burke, *The Journal of Chemical Physics* **1996**, *105*, 9982-9985; eJ. P. Perdew, K. Burke, M. Ernzerhof, *Physical Review Letters* **1997**, *78*, 1396-1396.

[s7] A. Schäfer, C. Huber, R. Ahlrichs, *The Journal of Chemical Physics* **1994**, *100*, 5829-5835.

[s8] (a) S. Grimme, J. Antony, S. Ehrlich, H. Krieg, *The Journal of Chemical Physics* **2010**, *132*, 154104; (b) S. Grimme, S. Ehrlich, L. Goerigk, *Journal of Computational Chemistry* **2011**, *32*, 1456-1465.
